# Deep sequencing of small RNA facilitates tissue and sex associated microRNA discovery in zebrafish

**DOI:** 10.1186/s12864-015-2135-7

**Published:** 2015-11-16

**Authors:** Candida Vaz, Choon Wei Wee, Gek Ping Serene Lee, Philip W. Ingham, Vivek Tanavde, Sinnakaruppan Mathavan

**Affiliations:** Lee Kong Chian School of Medicine, Nanyang Technological University, 11 Mandalay Road, Singapore, 308232 Singapore; Genome Institute of Singapore, Agency for Science Technology and Research, 60 Biopolis Street, #02-01 Genome, Singapore, 138672 Singapore; Bioinformatics Institute, Agency for Science Technology and Research, 30 Biopolis Street, #07-01 Matrix, Singapore, 138671 Singapore; Molecular Genomics (P) Ltd, 51 Science Park Road, #04-16 The ARIES, Singapore, 117586 Singapore; Institute of Medical Biology, Agency for Science Technology and Research, 8A Biomedical Grove, #06-06 Immunos, Singapore, 138648 Singapore; Institute of Molecular and Cell Biology, Agency for Science Technology and Research, 61 Biopolis Drive, Singapore, 138673 Singapore

**Keywords:** Zebrafish, Tissue associated miRNA, Sex associated miRNA, Novel miRNA prediction

## Abstract

**Background:**

The role of microRNAs in gene regulation has been well established. The extent of miRNA regulation also increases with increasing genome complexity. Though the number of genes appear to be equal between human and zebrafish, substantially less microRNAs have been discovered in zebrafish compared to human (miRBase Release 19). It appears that most of the miRNAs in zebrafish are yet to be discovered.

**Results:**

We sequenced small RNAs from brain, gut, liver, ovary, testis, eye, heart and embryo of zebrafish. In brain, gut and liver sequencing was done sex specifically. Majority of the sequenced reads (16–62 %) mapped to known miRNAs, with the exception of ovary (5.7 %) and testis (7.8 %). Using the miRNA discovery tool (miRDeep2), we discovered novel miRNAs from the unannotated reads that ranged from 7.6 to 23.0 %, with exceptions of ovary (51.4 %) and testis (55.2 %). The prediction tool identified a total of 459 novel pre-miRNAs. We compared expression of miRNAs between different tissues and between males and females to identify tissue associated and sex associated miRNAs respectively. These miRNAs could serve as putative biomarkers for these tissues. The brain and liver had highest number of tissue associated (22) and sex associated (34) miRNAs, respectively.

**Conclusions:**

This study comprehensively identifies tissue and sex associated miRNAs in zebrafish. Further, we have discovered 459 novel pre-miRNAs (~30 % seed homology to human miRNA) as a genomic resource which can facilitate further investigations to understand miRNA-mRNA gene regulatory networks in zebrafish which will have implications in understanding the function of human homologs.

**Electronic supplementary material:**

The online version of this article (doi:10.1186/s12864-015-2135-7) contains supplementary material, which is available to authorized users.

## Background

The zebrafish has acquired scientific importance over the years and has become a powerful tool for unravelling the role of human genes. There are several reasons that make zebrafish as one of the most sought out organisms for biomedical research. High fecundity, fast growth rate, short generation time, ease of maintenance and an abundant supply of research material are some of the reasons for considering this model. Further, the availability of a number of genomic tools and the transparent embryos facilitate the non invasive strategy to observe experimental effects [1]. Most importantly, comparison of human reference genome shows that at least 70 % of the genes have one zebrafish orthologue [2]. Of the human genes identified with morbidity descriptions in Mendelian Inheritance in Man (OMIM) database, about 82 % can be related to at least one zebrafish orthologue [2]. This high genomic similarity between zebrafish and human has resulted in important discoveries in the areas of human diseases, drug screens and therapeutic measures [3–5].

MicroRNAs (miRNAs) are a major class of non-coding RNA molecules that have steadily gained impetus over the past decade. They regulate several genes post-transcriptionally and serve as an added layer of control, being termed as “Micromanagers of gene expression” [6]. The expression patterns of miRNAs represent the physiological state of the cell and thus have an essential prognostic capacity [7]. Previous studies have revealed that comprehensive expression profiling of miRNA is useful in determining their specific expression patterns in cells [8], disease conditions such as cancer [9], and during cell differentiation [10, 11].

There are several experimental methods for profiling miRNAs such as northern blotting [12], RT-PCR [13], microarrays [14] and RAKE assay [15]. Each of these methods has their own limitations and advantages. Some of these methods are either cumbersome for scaling up and some are not sensitive enough to detect the low expressing miRNAs. The short length of these miRNAs also creates technical difficulties in the detection of mature miRNAs; further the above techniques are not suited to discover new miRNAs from the system.

With the advent of Next Generation sequencing (NGS) technology, there has been an increase in the efficiency of miRNA discovery. It not only overcomes the limitations of other methods but also provides an absolute and quantitative expression measurement [16, 17]. NGS technology facilitates profiling of known miRNA and discovery of new transcripts. Owing to its high sensitivity, this technology can identify low abundant miRNA transcripts, which may not be detected by other methods.

In zebrafish, miRNA expression profiling and discovery was initially carried out through small RNA cloning and microarray analysis. MicroRNA expression was detected through large scale sequencing of sRNA libraries prepared from different developmental stages of zebrafish and two adult cell lines [18] and this study identified about 154 mature miRNAs. Further this study revealed that the early zygotic stage (0 h) stage is nearly devoid of miRNAs. The expression of miR-430 family peaks at the blastula stage (4 h) and dominates the miRNA profile up to the pharyngula stage (24 h) and then decreases. Role of miR-430 in maternal RNA clearance during maternal to zygotic transition has been well documented [19]. Kloosterman and co-workers identified 139 known and 66 new miRNA from 5-day old zebrafish larvae and adult zebrafish brain; using in situ hybridization and northern blotting they identified developmental stage specific and tissue specific expression patterns for some of these miRNAs [20].

Using pyrosequencing technology to discover miRNAs, Soares et al. retrieved 90 % of the known miRNAs and predicted 25 novel miRNAs from different developmental stages and fully developed organs of zebrafish [21].

One of the recent publications used NGS technology to determine the temporal expression patterns for miRNAs and piRNAs during early embryonic development of zebrafish [22]. In this study, they identified a number of known miRNA, 8 novel miRNAs and a diverse set of piRNAs [22]. Based on all the above mentioned studies, a total number of 247 mature and 344 pre-miRNAs were identified and submitted as genomic resource for zebrafish (miRBase Release 19). However, no attempt has been made to identify tissue associated or sex associated miRNAs from the known miRNA resource. MicroRNA database (miRBase) [23–27] has a total of ~2000 mature miRNAs for human and only about 247 miRNAs for zebrafish (miRBase Release 19). It is known that number of coding genes in human and zebrafish are almost same. However, zebrafish miRBase has substantially low number of miRNA compared to human. Thus it appears that most of the miRNA in zebrafish are yet to be discovered.

Using NGS technology we undertook a systematic and comprehensive approach to determine the tissue/sex associated expression patterns of known miRNAs and to discover novel miRNAs from different tissues of zebrafish such as the brain, gut, liver, ovary, testis, eye, heart and embryo. For tissues such as brain, gut and liver, smallRNA from both the male and female counterparts were sequenced separately. The aim of our study was to discover new miRNAs from different tissues and to identify tissue associated and sex associated expression of known and novel miRNAs. In this work we generated about 20 M reads per library (and 3 biological replicates) which is significant enough to determine expression profiling and to discover novel miRNAs. The expression pattern of miRNAs associated to each tissue and gender can serve as a useful resource for further research and for understanding the miRNA-mRNA regulatory mechanisms in zebrafish. We have also discovered 459 putative novel pre-miRNAs, of which about 30 % had human seed homologs. This study adds a significant genomic resource to the zebrafish miRNA landscape and has potential to provide better understanding of miRNA based gene regulation in zebrafish.

## Results

### MicroRNA frequency distribution in the sRNA datasets

Zebrafish small RNA deep sequencing data from different tissues and embryo was generated using Illumina HiSeq 2000 platform. On the whole we sequenced 33 small RNAseq libraries comprising of 11 samples (embryo, male brain, female brain, male gut, female gut, male liver, female liver, ovary, testis, eye and heart) each with three biological replicates and approximately 20 million reads/library (for details see "Methods"). The details of the number of reads for each library are shown in Table 1.

**Table 1:** Details of the zebrafish sRNA deep sequencing datasets

Library	Replicates	Total number of reads	Total number of reads (in millions)	Percentage of reads mapped
Embryo	Embryo1	7,565,225	7.6	94.4
	Embryo2	10,202,969	10.2	94.8
	Embryo3	32,247,131	32.2	94.8
Male Brain	Male Brain1	5,101,418	5.1	97.8
	Male Brain2	6,533,205	6.5	97.3
	Male Brain3	10,518,347	10.5	97.0
Female Brain	Female Brain1	2,338,429	2.3	96.7
	Female Brain2	3,278,013	3.3	97.5
	Female Brain3	12,648,618	12.6	97.8
Male Gut	Male Gut1	16,232,210	16.2	97.1
	Male Gut2	8,864,108	8.9	97.7
	Male Gut3	3,117,877	3.1	95.8
Female Gut	Female Gut1	13,939,922	13.9	97.4
	Female Gut2	8,260,495	8.3	97.8
	Female Gut3	7,493,835	7.5	97.9
Male Liver	Male Liver1	18,404,267	18.4	97.8
	Male Liver2	9,369,428	9.4	97.4
	Male Liver3	4,639,550	4.6	97.1
Female Liver	Female Liver1	5,060,891	5.1	97.0
	Female Liver2	8,162,716	8.2	97.2
	Female Liver3	1,646,108	1.6	96.4
Ovary	Ovary1	8,824,267	8.8	95.8
	Ovary2	14,310,754	14.3	95.9
	Ovary3	25,898,221	25.9	95.9
Testis	Testis1	17,427,858	17.4	92.5
	Testis2	20,235,869	20.2	92.2
	Testis3	14,203,535	14.2	92.5
Eye	Eye1	9,398,588	9.4	96.0
	Eye2	5,722,393	5.7	95.5
	Eye3	2,854,316	2.9	95.9
Heart	Heart1	38,070,247	38.1	95.2
	Heart2	34,828,065	34.8	96.2

Each tissue library comprised of three biological replicates. One of the replicate of the heart did not cluster well with the other replicates and was not considered for further analysis. The table shows the number of processed (adaptor trimmed, > = 18nt) reads for each biological replicate. Around 92–98% of reads mapped to the zebrafish genome

**Fig. 1:**
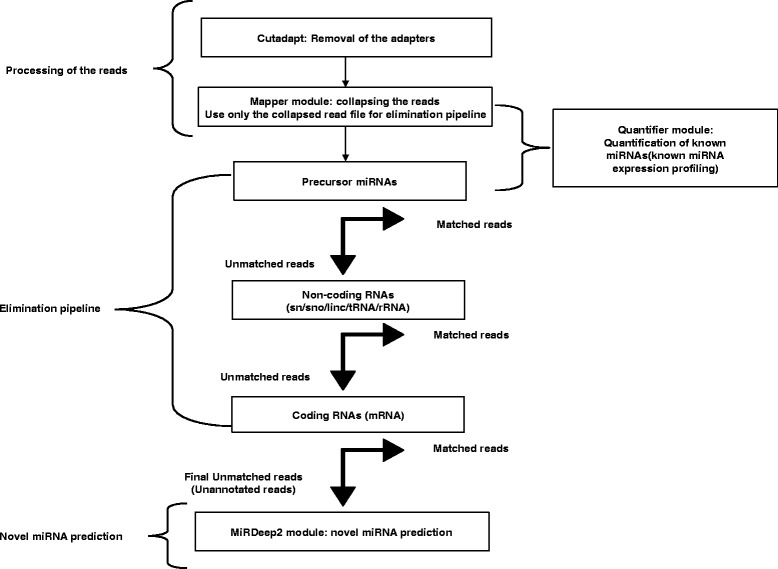
Flowchart depicting the workflow for known miRNA profiling and novel miRNA prediction. The workflow comprises of four parts namely: Processing of the reads, Quantification of known miRNAs, the Elimination pipeline and the Novel miRNA prediction pipeline. For the elimination pipeline, the reads were matched with the known annotated genomic sequences. Alignment with maximum of two mismatches was considered as matched reads. All the matched reads were removed before the next round of elimination. The quantification of known miRNAs and prediction of novel miRNAs was done using miRDeep2 modules

Datasets from each miRNA library (three biological replicates) generated in this work were matched to different annotated databases to classify the sRNA sequenced reads into different RNA categories (details in the “Methods” section) to get an overview of the frequency of different classes of RNA present in the samples as well as to obtain the unannotated pool of sequenced reads for novel miRNA prediction. The flowchart depicting the entire analysis pipeline is shown in Fig. 1.

**Fig. 2:**
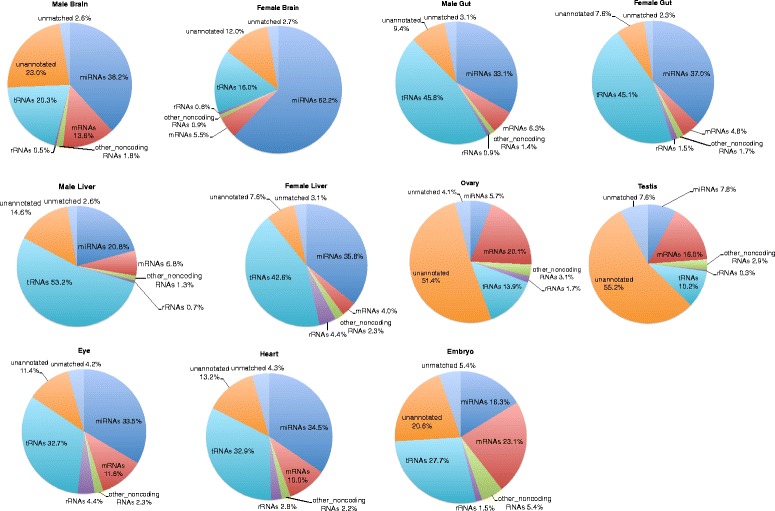
Pie charts depicting the frequency of different classes of RNA species present in the sRNA datasets. The pie charts represent an overview of the distribution of small RNAs in the different tissue libraries. For the male brain and female brain, the miRNAs constitute the most abundant sRNA, for the male gut, female gut, male liver and female liver, the tRNAs constitute the most abundant sRNA, and for the eye and heart, the miRNAs and tRNAs equally constitute the most abundant sRNAs. The ovary, testis and embryo had comparatively lesser amount of miRNAs. The ovary and testis had nearly half of their sRNA reads unannotated

In general, the sequenced reads of small RNA that mapped to the known miRNA database was abundant in female tissues compared to male tissues (female: brain 62.2; gut 37.0; liver 35.8; male: brain 38.2; gut 33.1; liver 20.8 %). Expression for eye and heart was tested only in male tissues and it amounted to 33.5 and 34.5 %, respectively. The sequenced reads mapping to known miRNA was low in embryo (16.3), ovary (5.7) and testis (7.8 %) and these samples had large number of unannotated reads. Small RNA seq data contained significant representation of tRNAs in most of the tissues, with the maximum amount in the gut (male 45.8; female 45.1 %) and liver (male 53.2; female 42.6 %) (Fig. 2). The mRNAs (small size/degraded/fragmented coding RNA) were more abundant in smallRNA seq data of the embryo and reproductive tissues (embryo 23.1; ovary 20.1 and testis 16.0 %) than in other tissues. rRNAs and other non-coding RNAs were the minimal RNA species in all the datasets ranging from 0.3 to 4.5 and 1–5.4 % respectively (Fig. 2). The percentage of unannotated mapped reads ranged from 7.6 to 23.0 % with exception of the ovary (51.4) and testis (55.2 %). The sequences that did not map to the genome ranged from 2.3 to 7.6 % (Fig. 2).

### Expression pattern of known miRNAs

**Fig. 3:**
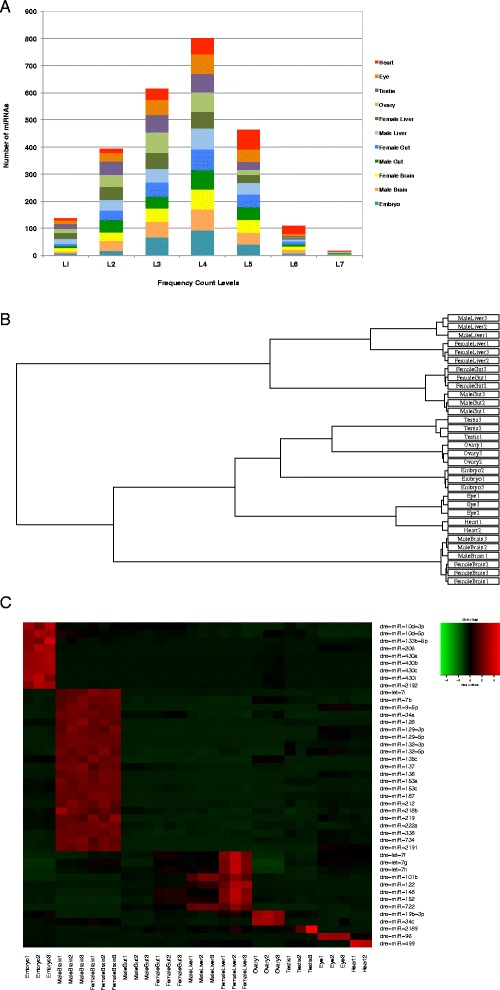
Tissue associated expression pattern of the known miRNAs. **a** Distribution of known miRNA expression levels with respect to number of miRNAs. Numbers of reads are taken as miRNA expression levels and their values are represented in the form of ranges: Level 1: 1–10, Level 2: 10–10^2^, Level 3: 10^2^–10^3^, Level 4: 10^3^–10^4^, Level 5: 10^4^–10^5^, Level 6: 10^5^–10^6^, Level 7:10^6^–10^7^. **b** Hierarchical clustering plot showing the similarity and differences among the samples. The known mature miRNA profiles of all the tissues were first subjected to TMM normalization and their clustering pattern was determined. There are two major groups seen:(i) The gut and the liver samples (ii) The brain, heart, eye, embryo, ovary and testis samples. The second major group is further divided into three subgroups:(a) The ovary and testis samples were closely placed, followed by the embryo (b) The eye and heart samples (c) The brain samples. **c** Expression profile of the tissue associated known miRNAs. Differential expression of tissue associated known mature miRNAs. The up-regulated miRNAs are depicted in red colour whereas the downregulated miRNAs are depicted in green colour

Out of the 247 known mature miRNAs, 86–96 % were detected by the quantifier module of miRdeep2 [28, 29] as they were found to be expressed in one or the other tissue sample (See “Methods”) (Additional file 1). The raw expression data/raw read counts of the mature miRNAs of replicates of each sample were pooled and binned. The known mature miRNAs showed a wide range of expression values spanning up to 7 levels of magnitude (Level 1: 1–10, 2: 10–10^2^, 3: 10^2^–10^3^, 4: 10^3^–10^4^, 5: 10^4^–10^5^, 6: 10^5^–10^6^, 7:10^6^–10^7^) (Fig. 3a). The expression pattern of the known mature miRNAs of all tissue samples showed almost normal distribution with the peak at Level 4. Maximum miRNAs had expression values within Level 4 (10^3^–10^4^), followed by Level 3 (10^2^–10^3^), Level 5 (10^4^–10^5^) and Level 2 (10–10^2^) ranges, respectively. Few miRNAs occurred in the lowest level, Level 1 (1–10) and second highest level, Level 6 (10^5^–10^6^), with the minimum at the highest level, Level 7 (10^6^–10^7^) (Fig. 3a).

### Clustering of the sRNA datasets

The known mature miRNA profiles of all the tissues were first subjected to Trimmed Mean of M-values (TMM) normalization (Additional file 1) using the Bioconductor package edgeR [30] as mentioned in the “Methods” section and their clustering pattern was determined using the hierarchical clustering plot (Fig. 3b). The data sets were also subjected to PCA analysis and clustering pattern obtained is presented in Additional file 2. The three biological replicates of all the tissue samples including embryo clustered closely indicating less experimental variation or noise among the replicates. However, one of the replicates of the heart sample did not cluster with the other replicates and hence was removed from all further analysis.

The tissue samples having male and female counterparts like the brain, gut and liver showed proximity among their male and female counterparts, indicating that the variations caused due to sex was less compared to the variations caused by the tissue. The samples were grouped as described in the hierarchical clustering plot (Fig. 3b) and the PCA plot (Additional file 2).

### Tissue associated known miRNAs

To determine the tissue associated known miRNAs, the TMM normalized expression profiles of the known mature miRNAs of each tissue was compared pairwise to the other tissue samples as well as to the embryo using Generalised linear model (GLM) edgeR analysis [30] (see “Methods”). Thus for each tissue a total of ten pairwise comparisons was conducted.

For samples such as the brain, gut and liver, the pairwise comparison was done with all the other tissues and embryo except with its male/female counterpart (a total of nine comparisons). The miRNAs that were found to be significantly up-regulated in a particular tissue and commonly down-regulated in the rest were called its tissue associated miRNAs (see “Methods”). The union of the tissue associated miRNAs for the male and female counterparts of brain, gut and liver were taken as their respective tissue associated miRNAs.

Similarly, to obtain embryo associated known miRNAs, the embryo was compared pairwise to all the tissues (ten comparisons). The miRNAs that were found to be significantly up-regulated in embryo and commonly down-regulated in all the other tissues were called “embryo associated”.

The brain had the highest number of known tissue associated miRNAs (22), followed by the liver (8), ovary (2), testis (1), eye (1), and heart (1). There were no significant gut associated miRNAs detected. 9 embryo associated miRNAs were identified. (Table 2, Fig. 3c).

**Table 2:** List of the tissue associated known miRNAs

Embryo	Brain	Liver	Ovary	Testis	Eye	Heart
dre-miR-10d-3p	dre-let-7i	dre-let-7f	dre-miR-19b-3p	dre-miR-2189	dre-miR-96	dre-miR-499
dre-miR-10d-5p	dre-miR-7b	dre-let-7g	dre-miR-34c			
dre-miR-133b-5p	dre-miR-9-5p	dre-let-7h				
dre-miR-206	dre-miR-34a	dre-miR-101b				
dre-miR-430a	dre-miR-128	dre-miR-122				
dre-miR-430b	dre-miR-129-3p	dre-miR-148				
dre-miR-430c	dre-miR-129-5p	dre-miR-152				
dre-miR-430i	dre-miR-132-3p	dre-miR-722				
dre-miR-2192	dre-miR-132-5p					
	dre-miR-135c					
	dre-miR-137					
	dre-miR-138					
	dre-miR-153a					
	dre-miR-153c					
	dre-miR-187					
	dre-miR-212					
	dre-miR-218b					
	dre-miR-219					
	dre-miR-222a					
	dre-miR-338					
	dre-miR-734					
	dre-miR-2191					

The brain had the highest number of known tissue associated miRNAs (22). There were no significant gut associated miRNAs

A number of tissue associated miRNAs reported by earlier studies involving in situ hybridisation and Northern Blots [20] were also detected in our analysis in the respective tissues. For example, miRNAs such as : dre-miR-135c and dre-miR-734 were found to be highly expressed in brain, dre-miR-122 was found to be associated to liver, and dre-miR-499 was found to be highly enriched in heart. The dre-miR-459-3p found to be specific to anterior part of the gut, was also detected in our analysis as enriched in gut, but since it was also present in the liver samples and was not significantly down-regulated in liver in comparison to the gut, we did not refer to it as gut associated. The dre-miR-430 miRNA family was highly enriched in embryo [20] was also detected by us.

### Sex associated known miRNAs

**Fig. 4:**
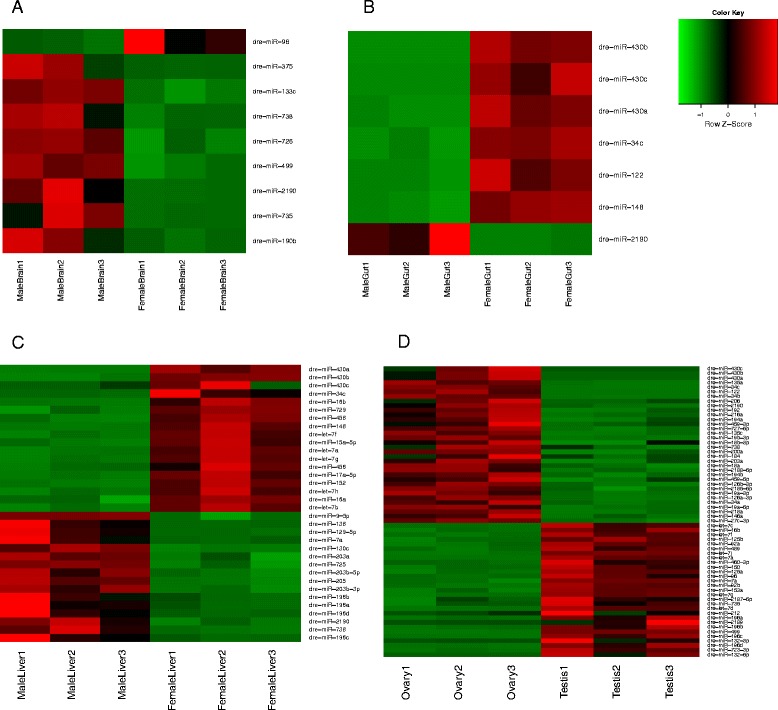
Expression profile of the sex associated known miRNAs. Differential expression of sex associated known mature miRNAs for (**a**) brain (**b**) gut (**c**) liver (**d**) ovary vs testis. The up-regulated miRNAs are depicted in red colour whereas the down-regulated miRNAs are depicted in green colour

To determine the sex associated known miRNAs, the TMM normalized expression profiles of known mature miRNAs of the male and female counterparts of the brain, gut and liver were compared using the GLM edgeR analysis [30] to obtain differentially expressed (DE) mature miRNAs. The DE mature miRNAs among the male and female counterparts were deemed as the sex associated miRNAs. The liver had the highest number of sex associated miRNAs (34), followed by the brain (9) and gut (7) (Table 3 and Fig. 4a-c). Details of the sex associated miRNAs including their log Fold Change, logCount Per Million, P-value and False Discovery Rate (FDR) are given in Additional file 3. The miRNA dre-miR-2190 was found to be commonly sex associated for brain, gut and liver, enriched in the male counterparts. The miRNAs dre-miR-34c, dre-miR-148, dre-miR-430a, dre-miR-430b, dre-miR-430c, were found to be commonly sex associated for gut and liver, enriched in the female counterparts. On comparison of the ovary and testis samples, a total of 63 miRNAs were found to be differentially expressed among them (Fig. 4d). Details of the DE mature miRNAs among ovary and testis are given in Additional file 3. The miRNAs dre-miR-34c, dre-miR-430a, dre-miR-430b, dre-miR-430c were also enriched in the ovary as compared to the testis as seen for the female counterparts of the gut and liver.

**Table 3:** List of the sex associated known miRNAs

Male Brain	Female Brain	Male Gut	Female Gut	Male Liver	Female Liver	Testis	Ovary
dre-miR-133c	dre-miR-96	**dre-miR-2190**	**dre-miR-34c**	dre-miR-7a	dre-let-7a	dre-let-7a	dre-miR-18a
dre-miR-190b			dre-miR-122	dre-miR-9-5p	dre-let-7b	dre-let-7c	dre-miR-18b-3p
dre-miR-375			dre-miR-148	dre-miR-129-5p	dre-let-7f	dre-let-7d	dre-miR-19a-3p
dre-miR-499			**dre-miR-430a**	dre-miR-130c	dre-let-7g	dre-let-7f	dre-miR-19a-5p
dre-miR-726			**dre-miR-430b**	dre-miR-138	dre-let-7h	dre-let-7g	dre-miR-19b-3p
dre-miR-735			**dre-miR-430c**	dre-miR-196a	dre-miR-15a-5p	dre-let-7j	dre-miR-27c-3p
dre-miR-738				dre-miR-196b	dre-miR-16a	dre-miR-7a	dre-miR-34a
**dre-miR-2190**				dre-miR-196c	dre-miR-16b	dre-miR-16b	dre-miR-34b
				dre-miR-196d	dre-miR-17a-5p	dre-miR-92a	**dre-miR-34c**
				dre-miR-203a	**dre-miR-34c**	dre-miR-92b	dre-miR-122
				dre-miR-203b-3p	dre-miR-148	dre-miR-96	dre-miR-126a-3p
				dre-miR-203b-5p	dre-miR-152	dre-miR-125a	dre-miR-126b-3p
				dre-miR-205	**dre-miR-430a**	dre-miR-125b	dre-miR-135a
				dre-miR-725	**dre-miR-430b**	dre-miR-132-3p	dre-miR-135c
				dre-miR-738	**dre-miR-430c**	dre-miR-132-5p	dre-miR-146a
				**dre-miR-2190**	dre-miR-456	dre-miR-150	dre-miR-184
					dre-miR-458	dre-miR-153a	dre-miR-192
					dre-miR-729	dre-miR-196a	dre-miR-194a
						dre-miR-196b	dre-miR-194b
						dre-miR-196c	dre-miR-200a
						dre-miR-196d	dre-miR-203a
						dre-miR-212	dre-miR-206
						dre-miR-460-3p	dre-miR-216a
						dre-miR-489	dre-miR-218a
						dre-miR-499	**dre-miR-430a**
						dre-miR-723-3p	**dre-miR-430b**
						dre-miR-735	**dre-miR-430c**
						dre-miR-2187-5p	dre-miR-459-3p
						dre-miR-2189	dre-miR-459-5p
							dre-miR-727-5p
							dre-miR-738
							dre-miR-2185-5p
							dre-miR-2188-5p
							dre-miR-2190

The liver had the highest number of sex associated miRNAs (34). The sex associated miRNAs are placed into two separate columns for the male and female counterparts, representing the male/female tissue in which it is up-regulated. Highlighted in bold font are the common sex associated miRNAs in at least three comparisons

The miRNAs dre-miR-430a, dre-miR-430b and dre-miR-430c were definitely most enriched in the embryo samples as compared to all the tissues and hence came up as embryo associated when the tissues were compared to embryo (Table 2).

However, these miRNA were more enriched in the female gut and liver as compared to their male counterparts. Similarly, the ovary was more enriched in these miRNAs as compared to the testis. For sex associated comparison, a pairwise comparison was made only between the male and female counterparts of the same tissue and hence these three miRNAs showed up as sexassociated as well. This phenomenon is overshadowed by the significantly higher abundance of these miRNAs in embryo (Additional file 4) compared to adult tissues. Since these miRNA are involved in maternal RNA degradation during early embryogenesis (19), it is possible that traces of these miRNA may still exist in the female tissues.

### Novel miRNA discovery

The reads that did not map to any of the known annotated data bases (unannotated reads) were identified through the elimination pipeline and these unannotated reads were used for novel miRNA prediction. By “novel” miRNA prediction we mean those miRNA that are seen for the first time in zebrafish and not existing in miRBase Release 19.

The unannotated sequences of the three replicates of a tissue were first combined together and then subjected to the miRDeep2.pl module [28, 29] for prediction of novel miRNAs. Since the three replicates were of different sequencing depths, it was essential to combine their unannotated reads before running the miRDeep2 module to obtain consistent predictions for each tissue.

To test the sensitivity of the miRDeep2, each sample was run through the miRDeep2 before being sieved through the elimination pipeline. The number of known miRNAs detected by miRDeep2 (at its default cut-off) in comparison to the total number of miRNAs in the particular sample was indicative of the sensitivity of miRDeep2. The sensitivity of miRDeep2 ranged from 89 to 95 % with the exception of one female liver sample, for which the sensitivity was 81 % (Additional file 5).

The Table 4 lists the number of novel pre-miRNAs predicted for each sample; and the details of the predicted novel pre-miRNAs including the information regarding the miRdeep score, mature miRNA read count, miRNAs with same seed region, consensus mature miRNA sequence, consensus precursor miRNA sequence, precursor miRNA genomic coordinates, for each tissue sample is given in Additional file 6. The Table 4 also shows the number of human seed homologs for the predicted novel miRNAs. The structure of a predicted novel pre-miRNA along with the reads aligned to it is shown in Additional file 7.

**Table 4:** Details of the novel pre-miRNAs predicted from different tissues

Tissue Samples	Number of novel miRNAs (unique)	Number of human seed homologs	Number of tissue associated novel miRNAs	Number of human seed homologs
Embryo	81 (78)	28 (36 %)	19	09
Male Brain	148 (147)	39 (27 %)	125	34
Female Brain	205 (193)	49 (25 %)		
Male Gut	73 (72)	23 (32 %)	15	08
Female Gut	89 (88)	34 (39 %)		
Male Liver	84 (75)	20 (27 %)	17	05
Female Liver	47 (46)	18 (39 %)		
Ovary	54 (50)	14 (28 %)	21	06
Testis	35 (34)	08 (24 %)	13	04
Eye	91 (89)	21 (24 %)	14	02
Heart	202 (197)	53 (27 %)	65	14

The table shows the total number of predicted novel pre-miRNAs as well as those that were specific for each tissue. The value in brackets is the unique number of novel pre-miRNAs predicted for each tissue. The miRDeep2.pl module also gives the seed homologs if present for each predicted novel miRNA. The table shows the number of predicted total and tissue associated novel miRNAs having human seed homologs. On an average 30% of the predicted novel pre-miRNAs had human seed homologs

### Expression pattern of novel predicted miRNAs

The novel pre-miRNAs detected from all the tissues including the embryo sample were grouped together and collapsed to obtain a non redundant set of novel pre-miRNAs. The analyses predicted a final set of 459 unique novel pre-miRNAs and were given an identifier prefix: “gis-dre-mir”. The Additional file 8 comprises of the precursor sequences of the 459 predicted novel miRNAs with their mature sequences in fasta format.

**Fig. 5:**
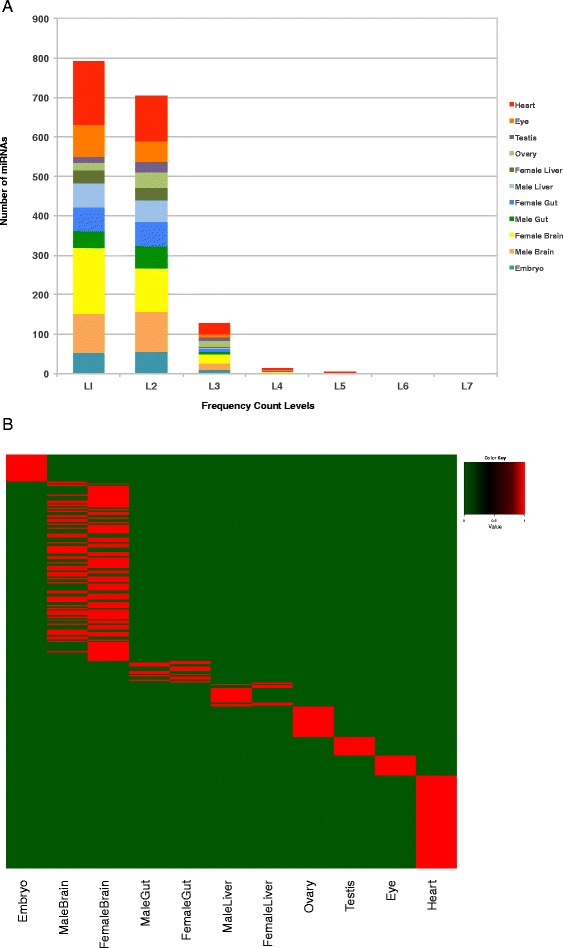
Tissue associated expression pattern of the novel miRNAs. **a** Distribution of novel miRNA expression levels with respect to number of miRNAs. Numbers of reads are taken as miRNA expression levels and their values are represented in the form of ranges. Level 1: 1–10, Level 2: 10–10^2^, Level 3: 10^2^–10^3^, Level 4: 10^3^–10^4^, Level 5: 10^4^–10^5^, Level 6: 10^5^–10^6^, Level 7:10^6^–10^7^. **b** Expression profile of the tissue associated novel pre-miRNAs. Differential expression of tissue associated novel pre-miRNAs. The presence of a novel pre-miRNA is depicted by red colour whereas the absence of it is depicted by green colour

The predicted novel mature miRNAs showed a skewed distribution of expression unlike the known mature miRNAs with the peak at the lowest level Level 1: 1–10 and a decreasing trend toward the higher levels. There were almost no miRNAs at the highest levels Level 6: 10^5^–10^6^ and Level 7:10^6^–10^7^ (Fig. 5a). This verifies the previous findings that the most abundant miRNAs are already known and the novel miRNAs tend to be rare and low in expression [20].

Further ,we carried out a “BLAST” search of our predicted novel zebrafish miRNA with all the miRNAs in miRBase (Current Release 21). If our novel miRNA sequence matched with any miRNA sequence in miRBase Release 21, with 100 % identity in more than 3/4th of its length, it was considered as a match. Out of our 459 predicted novel precursor sequences, 5 matched with the precursor miRNAs in miRBase Release 21. Out of these 5 precursor miRNA matches, 2 were the newly added zebrafish miRNAs (dre-miR-7147, dre-miR-7148). The other 3 precursor miRNA matches were from its closely related species *Ictalurus punctatus.* We also carried out similar “BLAST” search, but with a shorter word size (7) of the mature sequences of our predicted novel miRNA with the mature miRNAs in miRBase Release 21. Around 13 mature miRNA sequences were found to have exact matches; out of these 4 were with the newly added zebrafish mature miRNAs (dre-miR-7146-5p, dre-miR-7147, dre-miR-7148-5p, dre-miR-7148-3p). The remaining 9 mature miRNA matches were with mature miRNAs from its closely related species *Ictalurus punctatus, Salmo salar and Cyprinus carpio* (Additional file 9).

### Tissue associated novel pre-miRNAs

The novel pre-miRNA sequences were compared among all the samples including the embryo sample to obtain tissue associated predicted novel pre-miRNAs as well as embryo associated predicted novel pre-miRNAs. Additional file 10 gives the schematic representation of the method to obtain these tissue associated novel pre-miRNAs. The number of predicted tissue associated novel pre-miRNAs for each sample is shown in Table 4 and Fig. 5b. The brain showed the highest number of predicted tissue associated novel pre-miRNAs (125) followed by the heart (65). The details of these predicted tissue associated novel pre-miRNAs are given in Additional file 11.

### qPCR validations

**Fig. 6:**
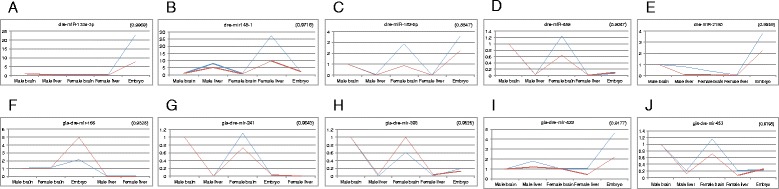
miRNA expression levels using qPCR and miRNA sequencing show a good correlation. (**a**–**e**) Known miRNAs (**f**–**j**) Novel miRNAs. Blue line denotes expression values based on qPCR and red line denotes expression values based on miRNA sequencing. A correlation comparison was done between the expression profiles from both platforms and the values are shown in brackets

Some of the known and novel predicted miRNAs were validated through qPCR and their expression patterns were compared with the expression patterns obtained through miRNAseq. The expression pattern obtained by RNA seq and qPCR was compared for selected miRNAs as a validation strategy. A total of 8 known miRNAs and 13 novel miRNA were tested by qPCR, out of which 5/8 known and 12/13 novel miRNAs showed similar patterns with correlation values above 0.5. Mostly, those which have low tag counts did not show significant correlation between miRNA seq and qPCR (see “Methods”). All the 5 known miRNAs having correlation above 0.5 and the top 5 among the 12 novel miRNAs are shown in Fig. 6.

## Discussion and conclusions

This study involves an exhaustive small RNA deep sequencing from several tissues and the embryo of zebrafish and systematic and comprehensive analysis of the data. The major focus was first to profile and identify tissue associated and sex associated known miRNAs and second to discover novel miRNAs.

Generally, RNAseq tag density of 1–2 M reads is good enough for miRNA expression profiling and, a tag density of 2–5 M reads is sufficient for discovery applications. We have generated about 20 M reads for each library in the current project, which is 4 to 10 times more than the required range. Further, we sequenced 3 biological replicates for most of the samples. Hence, our sRNA deep sequencing data had a high sequencing depth, deep enough for expression profiling and sensitive enough to discover lowly expressed novel miRNAs.

Around 92–98 % of the reads mapped to the zebrafish genome (Zv9) indicating minimal amount of degradation in the samples. The known miRNAs (344 precursors, 247 mature miRBase Release 19) comprised a wide range of the mapped reads (16–62 %), with the brain samples having the maximum amount (38–62 %) and with the ovary and testis having the minimum amount (6–8 %). The final unannotated pool of sequences that serves as a source of novel miRNA prediction was 7.6–23.0 % with exceptions of ovary (51.4 %) and testis (55.2 %) that had almost half of the sRNA sequences unannotated. The presence of such a high amount of unannotated sequences in ovary and testis suggests the presence of some unknown small non-coding RNAs such as piRNA that needs to be further analysed. It has been shown that piRNA are most abundant in reproductive tissues and early embryos [31, 32] .

The known mature miRNAs in each sample showed a wide range of expression values spanning 7 levels of magnitude. The expression values of the known miRNAs of all tissue samples showed an almost normal distribution.

To obtain tissue associated known miRNAs, the mature miRNA expression profile of each tissue sample was compared pairwise with the others to obtain the miRNAs significantly up-regulated in itself and commonly down-regulated in all the others. The miR-430 family was found to be very highly expressed in embryo with very low to none expression in adult tissues as seen in previous studies [18]. The miR-430 family has been shown to be involved in maternal RNA degradation during gastrulation [19]. Since our samples contained gastrulation stage embryo as well, it is logical to expect abundant presence of miR-430 in the sequence data. This miRNA family was found to be commonly down-regulated in all the adult tissues. The brain showed the highest number of tissue associated known miRNAs (22) pointing out to the fact that these miRNAs may have significant roles in brain development and functioning. Among the tissue associated miRNAs detected, some of them were reported in previous studies [20, 21] .

The sex associated miRNAs were obtained by comparing the mature miRNA expression profiles of the male and female counterparts of brain, gut and liver. The liver showed the highest number of sex associated known miRNAs (34), revealing major differences in the functioning of male and female livers. The miRNA dre-miR-2190 was found to be commonly sex associated for brain, gut and liver, enriched in the male counterparts. The miRNAs dre-miR-34c, dre-miR-430a, dre-miR-430b, dre-miR-430c, were found to be commonly sex associated for gut and liver, enriched in the female counterparts as well as ovary (on comparison with testis). For sex associated comparison, a pairwise comparison was made only between the male and female counterparts of the same tissue and hence the miRNAs 430a,b,c showed up as sexassociated as well. This phenomenon is overshadowed by the highest abundance of these miRNAs in embryo. Their specific expression in female liver and gut indicate that these miRNAs may be involved in functions in addition to maternal RNA degradation.

One of the most important applications of Next Generation Sequencing is the ability to detect novel miRNAs. The prediction of novel miRNAs was done by miRDeep2 software [28, 29] using the unannotated pool of sequences, left after the removal of the annotated sequences through the elimination pipeline. The sieving out of the known and annotated sequences through the elimination pipeline cuts down on the number of false predictions and increases the specificity of novel miRNA prediction.

Before using miRDeep2 for novel miRNA prediction, we ran the software on the unsieved and entire samples comprising of the annotated and unannotated reads. The number of known miRNA picked up by miRDeep2 in comparison to the total number of miRNAs in the samples at the similar cut-off used for novel miRNA prediction was used as an indicator of sensitivity. The sensitivity of miRDeep2 ranged from 89 to 95 % with the exception of one female liver sample, for which the sensitivity was 81 %.

A total of 459 novel pre-miRNAs were predicted in this study based on the sequence data. Majority of these novel miRNAs were less abundant and showed a skewed frequency distribution, with large number of miRNAs falling within the low levels of expression and very few within the high levels of expression. This is perhaps the reason they have not been discovered so far and highlights the merit of our really deep sequencing approach. These predicted novel miRNAs were also found to be having less seed conservation to human miRNAs (30 % on an average). These findings were in accordance to an earlier study that also reported less abundance and less conservation of the novel miRNAs [20].

The sequences of the predicted novel pre-miRNAs were compared with each other to obtain the ones associated to each tissue. Here again, the brain showed maximum number (125) tissue associated novel pre-miRNAs. It would be further interesting to correlate these tissue and sex associated miRNAs to their targets to understand the gene regulatory network and pathways regulating zebrafish development. The combination of the miRNA and their targets would certainly reveal the intricacies of regulation going on in each tissue.

In conclusion, even though ~247 mature miRNAs have been identified for zebrafish (miRBase19) we have categorized sex associated and tissues associated candidates and this attempt paves the path for tissue biomarker discovery. Addition of 459 novel pre-miRNAs will serve as a good resource for future research to understand miRNA-mRNA regulation in zebrafish. The high degree of similarity in the protein coding genes between zebrafish and humans promotes the application of discoveries in zebrafish to humans; functional analaysis of the novel zebrafish miRNAs will have implications in understanding their role in humans.

## Methods

### RNA Isolation and Libraries Construction

Total RNA were extracted using mirVana™ miRNA Isolation Kit (Life Technologies). For small RNA sequencing, total RNA was extracted from tissues (brain, gut, liver, ovary, testis, eye and heart) of adult fishes. For brain, gut and liver, the RNA was extracted from male and female fishes separately. For each tissue sample, 3 biological replicates were prepared; each biological replicate comprised of RNA extracted from tissues collected from 4 to 5 fishes. For the embryo RNA, early embryonic stages ( 2.5, 6, 12 and 24 hpf stage embryos; about 30 embryos per stage) and 15 day juveniles were pooled and RNA was extracted. Three replicates were prepared for each sample. On the whole, 33 small RNAseq libraries were prepared from 11 samples (embryo, male brain, female brain, male gut, female gut, male liver, female liver, ovary, testis, eye and heart) with three biological replicates for each sample. Wild type zebrafish were used for tissue/embryo collection for this study. (Animals used for this project maintained as per the Animal Care and Use Committee (IACUC) rules. The project was approved under Industrial Alignment program (ref No. IAF111050; project code: GIS/11-IAR210-1). Concentration and quality of the RNA was analyzed on Nanodrop (A_260_/A_280_ and A_260_/A_230_ ratios) and the integrity of the RNA was verified using RNA6000 chips on a Bioanalyzer (Agilent Technologies). Sequencing libraries for miRNA was constructed usingTruSeq® Small RNA Sample Prep Kit, Illumina. Sequencing library was prepared as per the instruction given in the manual.

### Sequencing

Sequencing was done on an Illumina HiSeq 2000 machine using TruSeq v3 cBot and SBS kits in Genome Institute of Singapore sequencing facility.

### Quantitative PCR

Quantitative PCR was performed to verify the miRNA sequencing using miRCURY LNA™ Universal RT microRNA PCR (Exiqon). 20 ng of total RNA from each pool was used as starting material and qPCR was setup according to manufacturer instruction. Cycling and data acquisition were performed on an ABI, 7900HT machine. The cycling conditions consist of a 10 min initial denaturation at 95 °C followed by 40 cycles of 95 °C for 10 s, 60 °C for 1 min. Data was acquired at the end of each annealing/extension step. The data was analysed with comparative Ct method (2–[delta][delta]Ct) using two miRNA housekeeping genes (dre-let-7a and dre-miR-10c).

### Processing of the reads

The reads were first subjected to adapter removal through the cutadapt program [33]. The trimmed reads were then collapsed to remove redundancy and to obtain a unique sequence fasta file through the mapper module of miRDeep2 package [28, 29]. The headers of the unique fasta file comprise of a running number and the frequency of the particular trimmed read (For details see Additional file 12).

### Annotation of the reads and the elimination pipeline

The unique reads fasta file was then put through an elimination pipeline module [34] comprising of a series of sequence similarity searches with the annotated databases. At each step the reads were matched to an annotated database with a maximum of two mismatches. The matched reads were removed and the unmatched ones were further matched to the next annotated database, finally culminating into an unannotated pool of reads that served as a source of novel miRNAs and novel sRNAs. The annotated databases used in the elimination pipeline were as follows:

i. Precursor miRNAs were obtained from miRBase, Release 19 comprising of 344 precursor miRNAs.
ii. Non coding RNA file ‘Danio_rerio.Zv9.71.ncrna.fa’ consisting of RNAs such as the miRNAs, rRNAs, sn/snoRNAs, lincRNAs and miscRNAs was downloaded from Ensembl FTP site.
iii. tRNAs (Zv9) were downloaded from http://gtrnadb2009.ucsc.edu/Dreri/ site.
iv. The‘rna.fa’ file comprising of majorly mRNAs was downloaded from NCBI FTP site.

The reads that matched to the annotated databases were assigned to that particular class of RNA and pie-charts were constructed indicative of the distribution of the annotated sRNA classes as well as the unannotated reads in a sample.

### Known miRNA expression profile generation, normalization and clustering

The known mature miRNA expression profile was generated by using the quantifier module of the miRDeep2 package [28, 29] that gives the read counts for the known miRNAs (For details see Additional file 12). The raw reads expression profile generated for all the replicates of the samples were subjected to Trimmed Mean of M-values (TMM) normalisation using the bioconductor package edgeR [30]. The normalized expression profiles for all the replicates of all the samples were then subjected to hierarchical clustering as well as PCA clustering for quality control purpose as well as to look at the similarity among the samples.

### Detection of tissue and sex associated known miRNAs

For determining the tissue associated known miRNAs, the TMM normalized expression profiles of the known mature miRNAs of each tissue was compared pairwise to the other tissue samples as well as to the embryo. The GLM framework in edgeR [30] was used and the treatDGE function was applied. A log Fold Change (logFC) change cut-off of 1, representing the size of the change and a p-value with FDR cut-off of < = 0.05 representing the significance of the change was used to get the differentially expressed miRNAs for each comparison. A total of ten pairwise comparisons were conducted for each tissue except for those with male/female counterparts. For tissues with male and female samples only 9 pairwise comparisons were conducted to exclude the male or female counterpart of the same tissue. In this case, a separate comparison was made for male and female brain, liver and gut and the union set of miRNAs in both these comparisons was considered specific to the particular tissue. The significantly up-regulated miRNAs in a particular tissue and commonly down-regulated miRNAs in all the other tissues that were compared with it, were taken as its respective tissue associated miRNAs.

For tissues that had male and female counterparts (brain, gut, liver), a union of the two sets was taken to get the complete number of tissue associated miRNAs. To obtain embryo associated known miRNAs, all the tissues were compared to embryo (ten comparisons). The miRNAs that were found to be significantly up-regulated in embryo and commonly down-regulated in all the other tissues were called “embryo associated”.

For determining the sex associated known miRNAs, the TMM normalized mature miRNA expression profiles of the female counterpart of the tissue was compared to the male counterpart (female vs male) using the the GLM framework in edgeR [30] and the treatDGE function. A log Fold Change (logFC) change cut-off of 1, representing the size of the change and a p-value with FDR cut-off of < = 0.05 representing the significance of the change was used to get the differentially expressed miRNAs. These differentially expressed miRNAs among the male and female were called as “Sex associated miRNAs”.

### Novel miRNA prediction pipeline

The unannotated sequences left after the elimination pipeline, were used for the novel miRNA prediction. For this purpose, the unannotated sequence files of the replicates of a particular tissue were combined and first mapped to the zebrafish genome using mapper.pl module and then subjected to the miRDeep2.pl module [28, 29] to obtain the novel miRNAs (For details see Additional file 12).

### Detection of tissue associated novel pre-miRNAs

The novel pre-miRNA sequences of all the tissues predicted by miRDeep2 were compared with each other including the embryo sample using an in-house sequence similarity search perl program that looks for equal sequence matches as well as substrings and overlaps to obtain the total matched set of novel pre-miRNA sequences. The total matched set was then subtracted from the total predicted set of novel pre-miRNA sequences to obtain the unmatched set of novel pre-miRNA sequences. The unmatched set of novel pre-miRNA sequences were deemed as tissue associated novel pre-miRNAs for each tissue and embryo associated novel pre-miRNAs for the embryo (Additional file 10).

The entire protocol comprising of processing of the reads, quantification of known miRNAs, elimination pipeline and novel miRNA prediction has been depicted in Fig. 1.

### Availability of supporting data

All the sRNA deep sequencing data sets generated in this project have been deposited in NCBI's Gene Expression Omnibus and are accessible through GEO Series accession number GSE57169. (http://www.ncbi.nlm.nih.gov/geo/query/acc.cgi?acc=GSE57169). The data sets are now publicly available and can be downloaded from this site. Novel miRNA sequence data sets have been submitted to to microRNA database (miRBase; http://www.mirbase.org/) and accessible from miRBase site.

## Additional files

Additional file 1:
**Comprises of the “Raw” and “TMM Normalized” known mature miRNA expression profiles of all the samples.** Sheet1: Raw, Sheet2: TMM Normalized. (XLSX 162 kb) Additional file 2:
**Principal Component Analysis (PCA) plot depicting the clustering of the samples.** The three biological replicates of all the tissue samples including embryo clustered closely indicating less experimental variation among the replicates. The tissue samples having male and female counterparts like the brain, gut and liver showed proximity among their male and female counterparts, indicating that the variations caused due to sex was less compared to the variations caused by the tissue. (PDF 238 kb) Additional file 3:
**Comprises of the details of the sex associated known miRNAs including the logFC, logCPM,** **P-value** **and FDR values.** Sheet1: brain, Sheet2: gut, Sheet3: liver, Sheet4: ovary vs. testis. (XLSX 20 kb) Additional file 4:
**Comprises of barcharts for validation.** Slide1: dre-miR-21 is found to be abundant and ubiquitously expressed as compared to some let-7 family members. The dre-let-7f, g, h appears to be enriched in female liver and dre-let7i is enriched in brain as compared to the embryo and other tissue samples. Slides 2 and 3 : shows the expression of the miRNAs 430 a,b,c in the embryo and the female tissues of gut, liver and ovary. The slides depict the overshadowing effect of high expression of these miRNAs in embryo over the female tissues. The high expression of these miRNAs in the female tissue is visible only when not compared to embryo. (PDF 227 kb) Additional file 5:
**Is a plot showing the sensitivity of MiRDeep2 for identification of known miRNAs from zebrafish.** The number of known miRNAs picked up by miRDeep2 in comparison to the total number of miRNAs in the sample; at the similar cut-off used for novel miRNA prediction was used as an indicator of its sensitivity. The sensitivity of miRDeep2 ranged from 89 to 95 % with the exception of one female liver sample, for which the sensitivity was 81 %. (PDF 26 kb) Additional file 6:
**Comprises of the details of the predicted novel miRNAs of all the samples including the IDs, miRDeep2 scores, read counts, precursor and mature sequences and precursor coordinates.** Sheet1: embryo predicted novel miRNAs, Sheet2: male brain predicted novel miRNAs, Sheet3: female brain predicted novel miRNAs, Sheet4: male gut predicted novel miRNAs, Sheet5: female gut predicted novel miRNAs, Sheet6: male liver predicted novel miRNAs, Sheet7: female liver predicted novel miRNAs, Sheet8: ovary predicted novel miRNAs, Sheet9: testis predicted novel miRNAs, Sheet10: eye predicted novel miRNAs, Sheet11: heart predicted novel miRNAs). (XLSX 159 kb) Additional file 7:
**Figure showing the structure of a predicted novel miRNA from miRdeep2 with its aligned read sequences.** MiRdeep2 gives as output the pdfs of the structure of the predicted novel miRNA along with the reads mapping to its mature, star and loop sequences. The top left corner has the score distribution for the predicted novel miRNA, the top right corner has the predicted hairpin structure for the novel pre-miRNA and the major part comprises of the alignment of the reads to the mature (red), loop (yellow) and the star regions (purple) of the precursor miRNA. For each read mapped, its frequency value, the number of mismatches with which it maps, and the sample it belongs to is given at the bottom right. (PDF 221 kb) Additional file 8:
**Comprises of the precursor sequences of the 459 predicted novel miRNAs along with their 5p and 3p mature miRNAs.** The novel predicted miRNAs are given the prefix : “gis-dre-mir”. (PDF 92 kb) Additional file 9:
**Comprises of BLAST matches of the predicted novel miRNAs to all the miRNAs in the current release 21 of miRBase.** (XLSX 52 kb) Additional file 10:
**Figure showing the schematic representation of obtaining specific novel** **pre-miRNAs** **for embryo.** The coloured circles represent the set of predicted novel pre-miRNAs for each tissue sample. The 78 predicted novel pre-miRNAs of embryo were compared with the set of predicted novel pre-miRNAs of other tissues to find the ones that matched. The total set of matched novel pre-miRNAs were 59. Therefore the unmatched set of 19 was considered as specific novel pre-miRNAs for embryo. This procedure was followed for the other tissue samples to obtain the novel pre-miRNAs specific to them. (PDF 30 kb) Additional file 11:
**Comprises of the details of the tissue associated predicted novel** **pre-miRNAs** **including the IDs, miRDeep2 scores, read counts, precursor and mature sequences and precursor coordinates.** Sheet1: embryo associated novel miRNAs, Sheet2: brain associated novel miRNAs, Sheet3: gut associated novel miRNAs, Sheet4: liver associated novel miRNAs, Sheet5: ovary associated novel miRNAs, Sheet6: testis associated novel miRNAs, Sheet7: eye associated novel miRNAs, Sheet8: heart associated novel miRNAs, Sheet9: summary). For tables containing information about the brain, gut and liver tissue associated novel miRNAs, there exists an additional column indicating whether a tissue associated novel miRNA is present in male/female or both. Some tissue associated novel miRNAs are present in both male and female and some in only one sex. The summary (Sheet9) gives the unique number of tissue associated novel miRNAs that reflects the union of the two. (XLSX 62 kb) Additional file 12:
**Comprises of additional details of the methods, including the options and parameters of the tools used for processing of the reads, known miRNA expression profile generation, normalization and clustering and novel miRNA prediction pipeline.** (PDF 1468 kb)

## Abbreviations

miRNA: microRNA
NGS: Next generation sequencing
DE: Differentially expressed
TMM: Trimmed mean of M-values
GLM: Generalised linear model
