# Supplementary material for: Deep sequencing of small RNA facilitates tissue and sex associated microRNA discovery in zebrafish
Source: BMC Genomics. 2015 Nov 16;16:950. doi: 10.1186/s12864-015-2135-7 (PMC4647824; doi:10.1186/s12864-015-2135-7)
Supplement: Additional file 2: — Principal Component Analysis (PCA) plot depicting the clustering of the samples. The three biological replicates of all the tissue samples including embryo clustered closely indicating less experimental variation among the replicates. The tissue samples having male and female counterparts like the brain, gut and liver showed proximity among their male and female counterparts, indicating that the variations caused due to sex was less compared to the variations caused by the tissue. (PDF 238 kb) [file 12864_2015_2135_MOESM2_ESM.pdf]

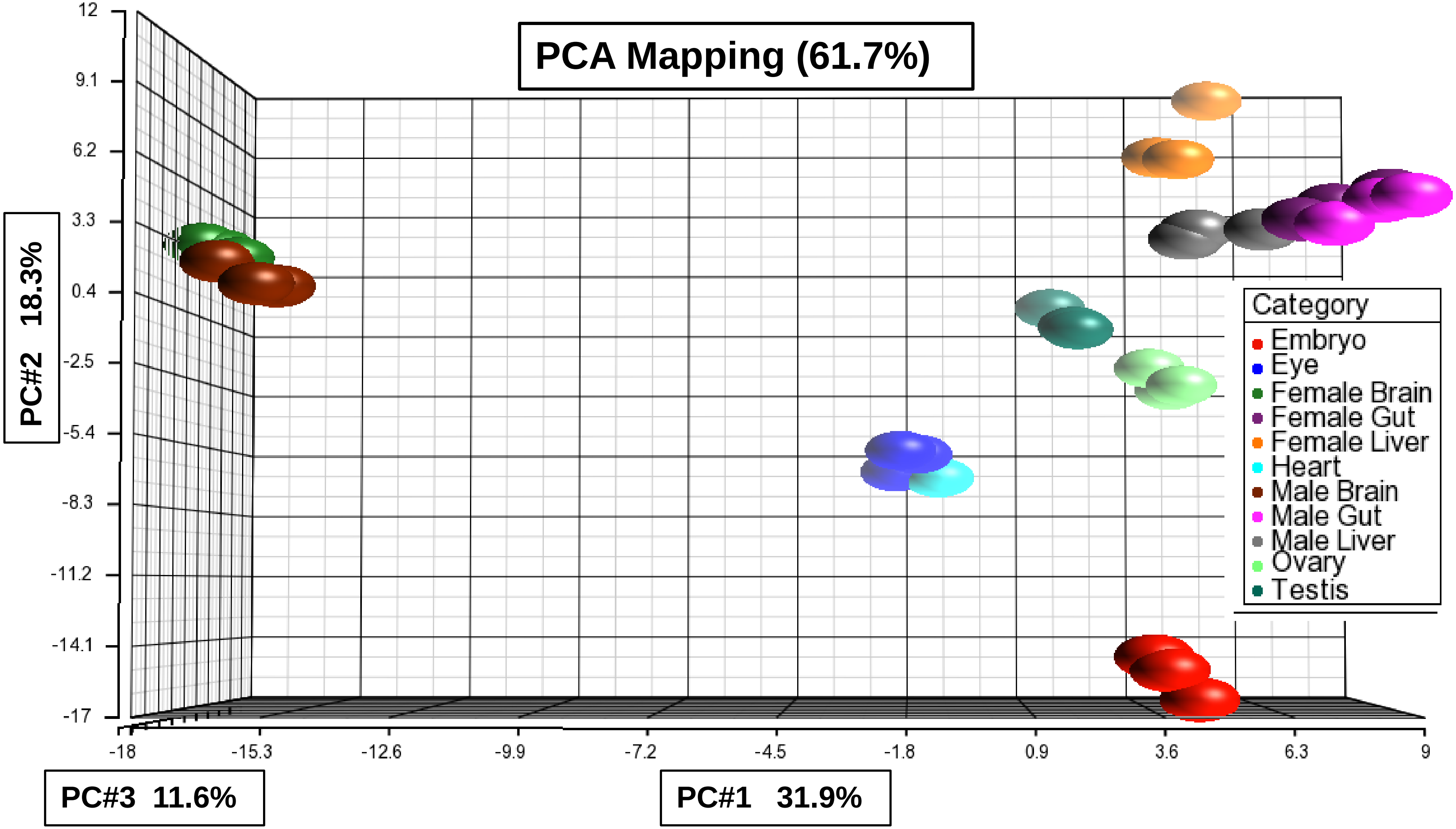
PCA Mapping (61.7%)
PC#2 18.3%
PC#1 31.9%
PC#3 11.6%
12
9.1
6.2
3.3
0.4
-2.5
-5.4
-8.3
-11.2
-14.1
-17
-18
-15.3
-12.6
-9.9
-7.2
-4.5
-1.8
0.9
3.6
6.3
9
Category
Embryo
Eye
Female Brain
Female Gut
Female Liver
Heart
Male Brain
Male Gut
Male Liver
Ovary
Testis
