# Supplementary material for: Deep sequencing of small RNA facilitates tissue and sex associated microRNA discovery in zebrafish
Source: BMC Genomics. 2015 Nov 16;16:950. doi: 10.1186/s12864-015-2135-7 (PMC4647824; doi:10.1186/s12864-015-2135-7)

Comparison of the expression of dre-miR-21 and dre-let-7 family members:

dre-miR-21 is highly abundant and ubiquitously expressed

dre-let7i appears to be enriched in brain samples as compared to other tissues

dre-let-7f, g, h appears to be enriched in all the three replicates of the female liver as compared to the embryo and other tissue samples

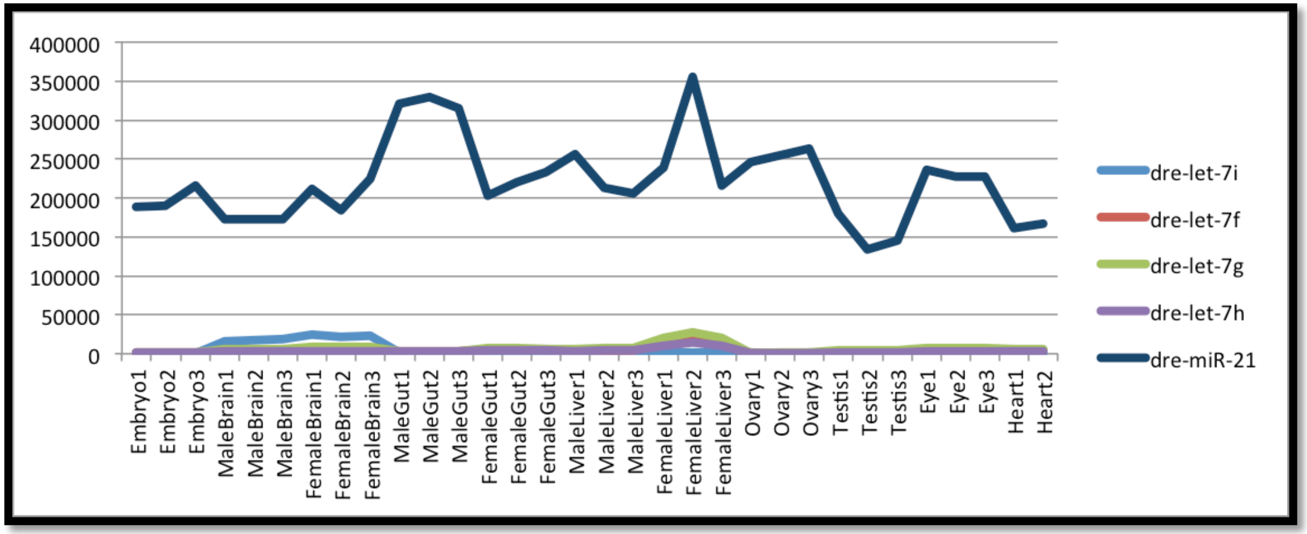

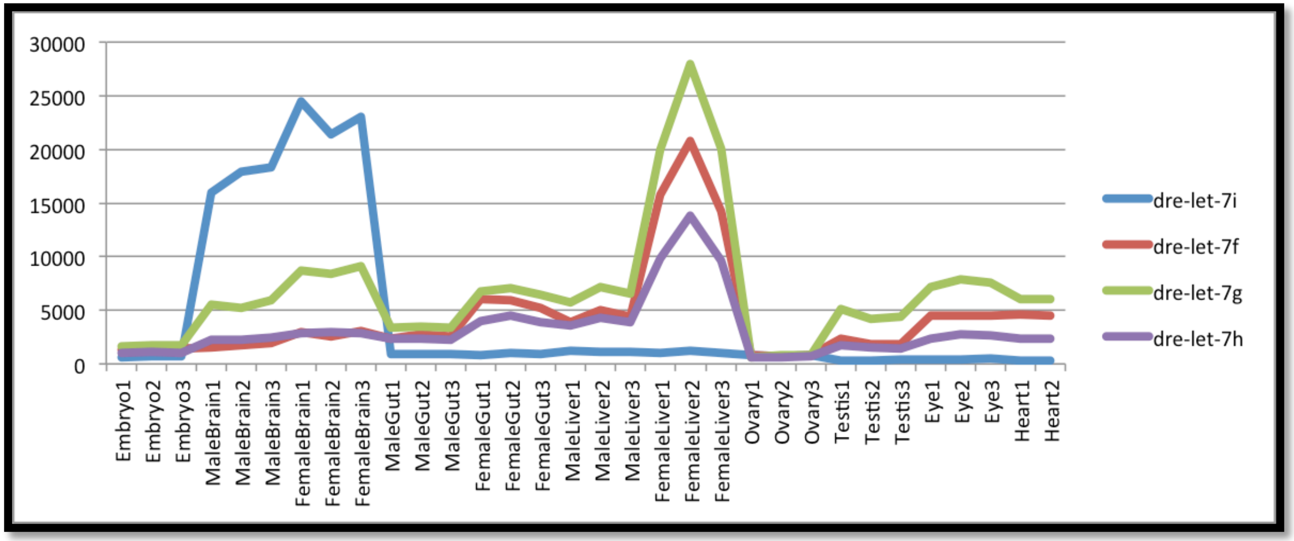

Embryo associated dre-miR-430a,b,c :

It is high in Embryo that it overshadows the expression of rest of the tissues. It is therefore Embryo specific.

RNA seq tag density is quite high in embryo compared to ovary

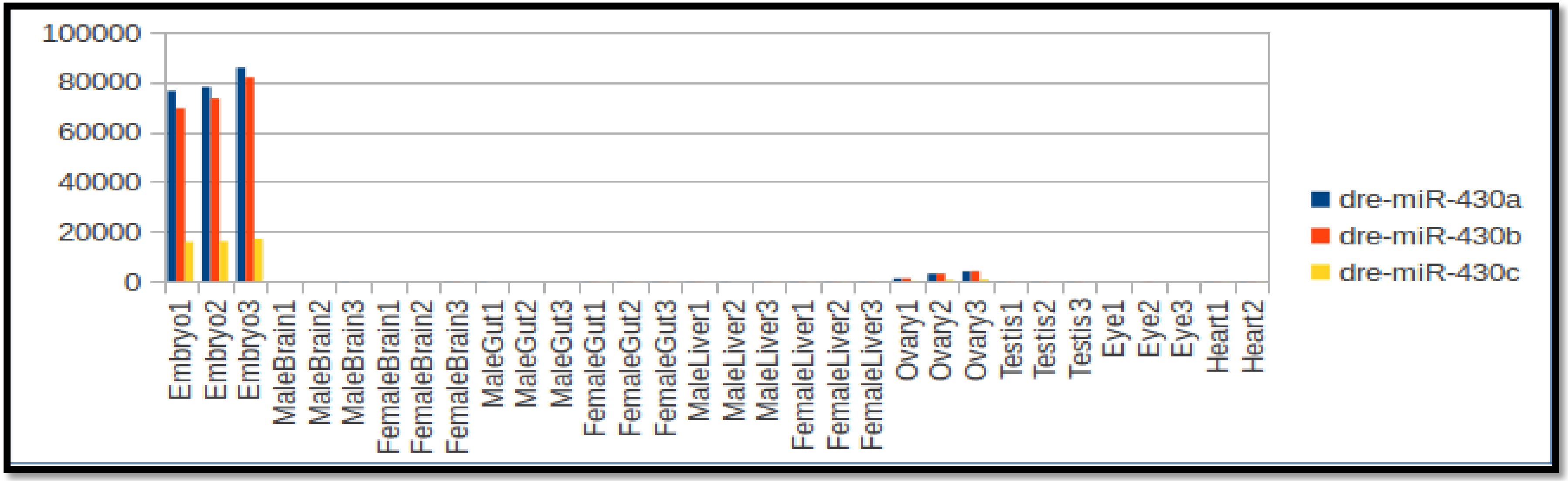

Ovary sex associated dre-miR-430a,b,c vs. Testis :

Clearly enriched in ovary compared to testis samples

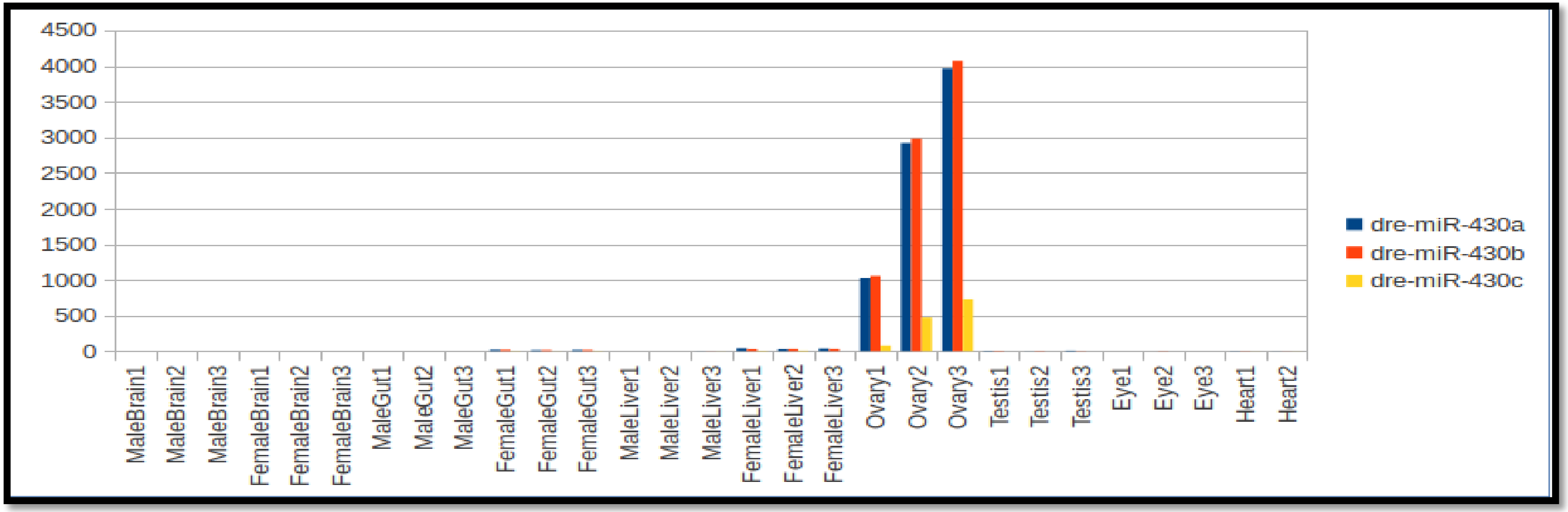

Female Gut and Female Liver sex associated dre-miR-430a,b,c vs. Male counterparts : Clearly enriched in female gut and liver compared to other tissues (ignoring Embryo, and Ovary samples; RNA seq tag density is low compared to embryo /ovary)

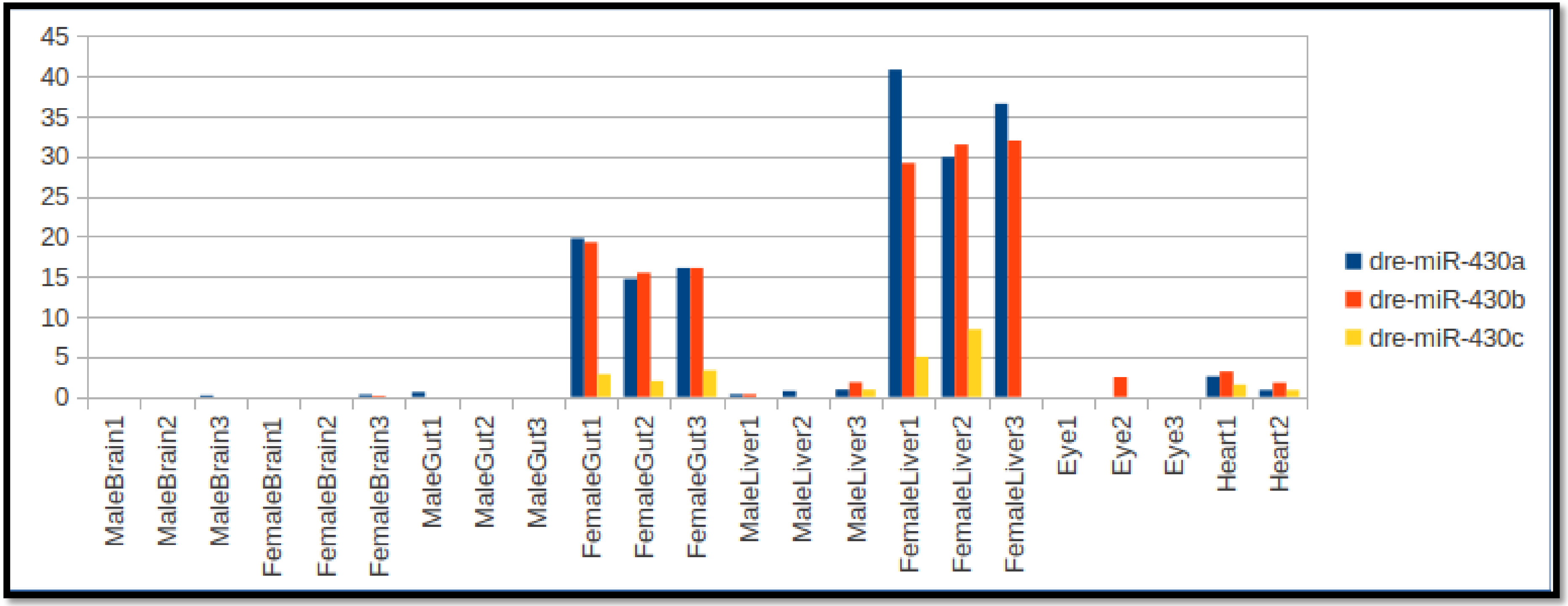

Supplement: Additional file 4: — Comprises of barcharts for validation. Slide1: dre-miR-21 is found to be abundant and ubiquitously expressed as compared to some let-7 family members. The dre-let-7f, g, h appears to be enriched in female liver and dre-let7i is enriched in brain as compared to the embryo and other tissue samples. Slides 2 and 3 : shows the expression of the miRNAs 430 a,b,c in the embryo and the female tissues of gut, liver and ovary. The slides depict the overshadowing effect of high expression of these miRNAs in embryo over the female tissues. The high expression of these miRNAs in the female tissue is visible only when not compared to embryo. (PDF 227 kb) [file 12864_2015_2135_MOESM4_ESM.pdf]
