# Supplementary figures and images for: Deep sequencing of small RNA facilitates tissue and sex associated microRNA discovery in zebrafish
Source: BMC Genomics. 2015 Nov 16;16:950. doi: 10.1186/s12864-015-2135-7 (PMC4647824; doi:10.1186/s12864-015-2135-7)

Sensitivity of detection  
of known miRNAs

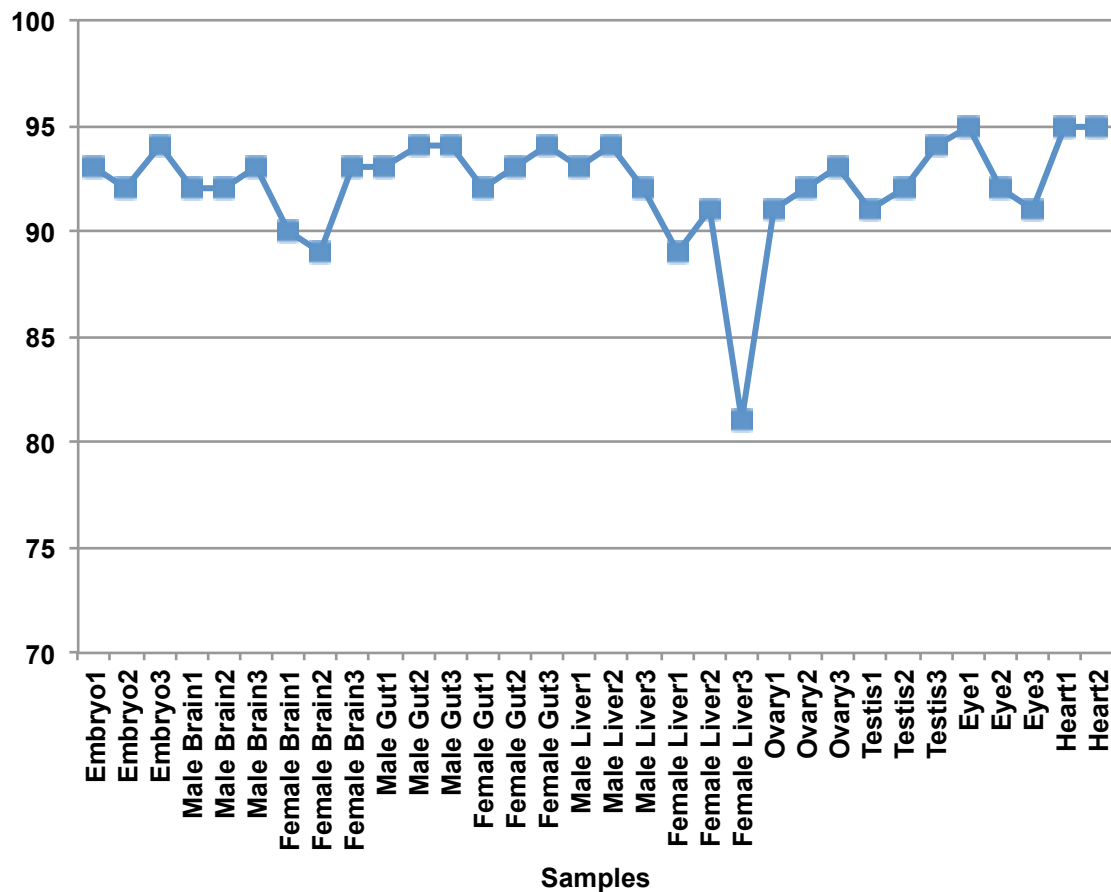

Supplement: Additional file 5: — Is a plot showing the sensitivity of MiRDeep2 for identification of known miRNAs from zebrafish. The number of known miRNAs picked up by miRDeep2 in comparison to the total number of miRNAs in the sample; at the similar cut-off used for novel miRNA prediction was used as an indicator of its sensitivity. The sensitivity of miRDeep2 ranged from 89 to 95 % with the exception of one female liver sample, for which the sensitivity was 81 %. (PDF 26 kb) [file 12864_2015_2135_MOESM5_ESM.pdf]
