# Supplementary material for: Deep sequencing of small RNA facilitates tissue and sex associated microRNA discovery in zebrafish
Source: BMC Genomics. 2015 Nov 16;16:950. doi: 10.1186/s12864-015-2135-7 (PMC4647824; doi:10.1186/s12864-015-2135-7)
Supplement: Additional file 7: — Figure showing the structure of a predicted novel miRNA from miRdeep2 with its aligned read sequences. MiRdeep2 gives as output the pdfs of the structure of the predicted novel miRNA along with the reads mapping to its mature, star and loop sequences. The top left corner has the score distribution for the predicted novel miRNA, the top right corner has the predicted hairpin structure for the novel pre-miRNA and the major part comprises of the alignment of the reads to the mature (red), loop (yellow) and the star regions (purple) of the precursor miRNA. For each read mapped, its frequency value, the number of mismatches with which it maps, and the sample it belongs to is given at the bottom right. (PDF 221 kb) [file 12864_2015_2135_MOESM7_ESM.pdf]

Provisional ID : gi_312144711_ref_NC_007130.5_DanioreriostrainTuebingenchromosome19,Zv9_6819
Score total : 423.2
Score for star read(s) : 3.9
Score for read counts : 418.2
Score for mfe : 1.7
Score for randfold : 0
Score for cons. seed : -0.6
Total read count : 832
Mature read count : 831
Loop read count : 0
Star read count : 1

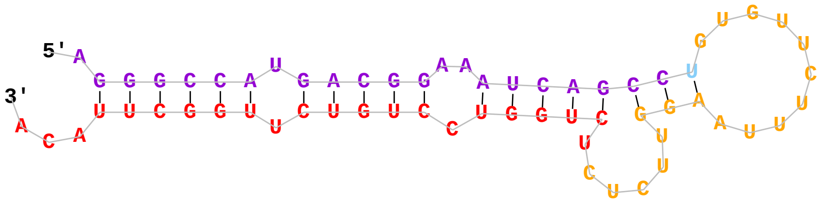

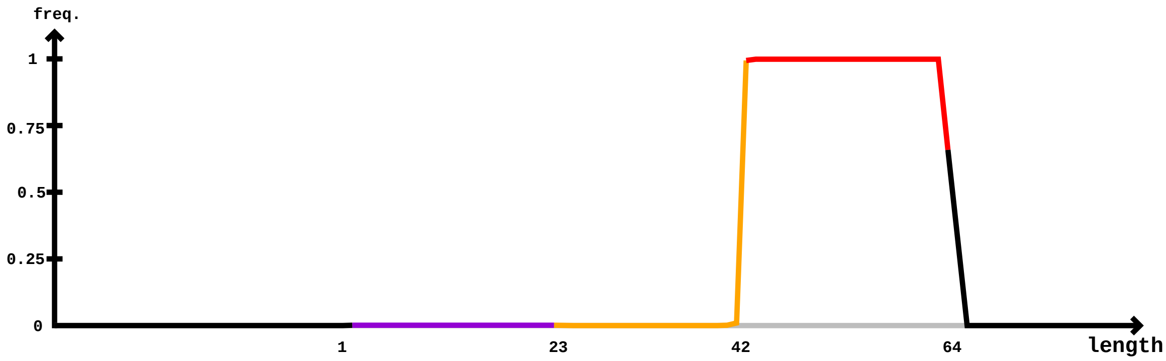

Star Mature

```
5'- cuucagucuauucuggcacucgaugucugagggccaugacggaaaucagccuguguucuuuaagguucucucugguccugucuuggcuuacaggcuugaguugcacugaca   -3'    obs
    cuucagucuauucuggcacucgaugucugagggccaugacggaaaucagccuguguucuuuaagguucucucugguccugucuuggcuuacaggcuugaguugcacugaca          exp
    ..(((((........((((((((.(((((..(((((.(((((..((((((((..........)))......))))).))))).)))))..))))))))))).)))))))..   reads    mm    sample
    .....................................................................cucugguccugucuuggcuuaca...................   1        0     EMF
    ......................................................................ucugguccugucuuggcuuaU....................   1        1     EMF
    ......................................................................ucugAuccugucuuggcuuac....................   14       1     EMF
    ......................................................................ucugguccugucCuggcuuac....................   1        1     EMF
    ......................................................................ucugguccugucuuggcuuac....................   37       0     EMF
    ......................................................................ucugAuccugucuuggcuuaca...................   22       1     EMF
    ......................................................................ucugguccugucuuggcuuacU...................   3        1     EMF
    ......................................................................ucugguccugucuuggcuuaca...................   62       0     EMF
    ......................................................................ucugguccugucuuggcuuacaA..................   1        1     EMF

    .............................agggccaugacggaaaucagcc............................................................   1        0     EMT
    ....................................................................ucucugguccugucuuggcuuac....................   1        0     EMT
    .....................................................................cucugguccugucuuggcuuac....................   3        0     EMT
    .....................................................................cucugguccugucuuggcuuacU...................   1        1     EMT
    .....................................................................cucugAuccugucuuggcuuaca...................   1        1     EMT
    ......................................................................ucugguccugucuugCcuuac....................   1        1     EMT
    ......................................................................ucuggCccugucuuggcuuac....................   1        1     EMT
    ......................................................................ucugguccugucuuggcuuac....................   101      0     EMT
    ......................................................................ucugAuccugucuuggcuuac....................   46       1     EMT
    ......................................................................ucuggCccugucuuggcuuaca...................   1        1     EMT
    ......................................................................ucugguccugucuuggcuuacU...................   11       1     EMT
    ......................................................................ucugguccugucuGggcuuaca...................   1        1     EMT
    ......................................................................ucugAuccugucuuggcuuaca...................   100      1     EMT
    ......................................................................ucugguccugucuuggcuuaca...................   188      0     EMT
    ......................................................................ucugguccugucCuggcuuaca...................   1        1     EMT
    ......................................................................ucugguccugucuuggcuuaUa...................   1        1     EMT
    ......................................................................ucugguccugucuuUgcuuaca...................   1        1     EMT
    ......................................................................ucugguccugucuuggcuuacaU..................   4        1     EMT
    ......................................................................ucugguccugucuuggcuuacaA..................   1        1     EMT
    .......................................................................cugAuccugucuuggcuuaca...................   2        1     EMT

    .....................................................................cucugAuccugucuuggcuuac....................   1        1     EMS
    ......................................................................ucuggCccugucuuggcuuac....................   1        1     EMS
```

Star Mature

```
cuucagucuauucuggcacucgaugucugagggccaugacggaaaucagccuguguucuuuaagguucucucugguccugucuuggcuuacaggcuugaguugcacugaca
......................................................................ucugguccugucuuggcuuac....................    54    0    EMS
......................................................................ucugguccugucuugNcuuac....................    1     1    EMS
......................................................................ucugAuccugucuuggcuuac....................    20    1    EMS
......................................................................ucugguccugucuuggcuuacU...................    7     1    EMS
......................................................................ucugguccAgucuuggcuuaca...................    1     1    EMS
......................................................................ucugAuccugucuuggcuuaca...................    28    1    EMS
......................................................................ucugguccugucuuggcuuaca...................    107   0    EMS
......................................................................ucugguccugucuuggcuuacaU..................    1     1    EMS
.......................................................................cugguccugucuuggcuuaca...................    1     0    EMS
.......................................................................cugAuccugucuuggcuuaca...................    1     1    EMS
```
