# Supplementary material for: Deep sequencing of small RNA facilitates tissue and sex associated microRNA discovery in zebrafish
Source: BMC Genomics. 2015 Nov 16;16:950. doi: 10.1186/s12864-015-2135-7 (PMC4647824; doi:10.1186/s12864-015-2135-7)
Supplement: Additional file 8: — Comprises of the precursor sequences of the 459 predicted novel miRNAs along with their 5p and 3p mature miRNAs. The novel predicted miRNAs are given the prefix : “gis-dre-mir”. (PDF 92 kb) [file 12864_2015_2135_MOESM8_ESM.pdf]

```
>gis-dre-mir-1
aaaauagcccuuguugaauuaaacguaaauggaagcuuaagcuauauguuaauucaacuagagcuguu
uag
>5p-matureseq
aaaauagcccuuguugaauua
>3p-matureseq
uaauucaacuagagcuguuuag
~~~~~~~~~~~~~~~~~~~~~~~~~~~~~~~~~~~~~~~~~~~~~~~~~~~
>gis-dre-mir-2
aaagcagccauugacauccagugggaacuaaaaauacuaugggaguugauggcuguuuuga
>5p-matureseq
aaagcagccauugacauccagugg
>3p-matureseq
augggaguugauggcuguuuuga
~~~~~~~~~~~~~~~~~~~~~~~~~~~~~~~~~~~~~~~~~~~~~~~~~~~
>gis-dre-mir-3
aaagcugauuacugugacugcagcuuuuacagugugcacugcagucacaguaaucagcuuu
>5p-matureseq
aaagcugauuacugugacugc
>3p-matureseq
ugcagucacaguaaucagcuuu
~~~~~~~~~~~~~~~~~~~~~~~~~~~~~~~~~~~~~~~~~~~~~~~~~~~
>gis-dre-mir-4
aaaguaacaagugucaagugacgauggguguccagucacuugacacuuguuacuuuuc
>5p-matureseq
aaaguaacaagugucaagugac
>3p-matureseq
cacuugacacuuguuacuuuuc
>3p-matureseq
ucacuugacacuuguuacuuuu
~~~~~~~~~~~~~~~~~~~~~~~~~~~~~~~~~~~~~~~~~~~~~~~~~~~
>gis-dre-mir-5
aacaacaauggaggacaugcagcagauccuguuccugauuucugcaugccgucauuguuuuau
>5p-matureseq
aacaacaauggaggacaugcagc
>3p-matureseq
cugcaugccgucauuguuuuau
~~~~~~~~~~~~~~~~~~~~~~~~~~~~~~~~~~~~~~~~~~~~~~~~~~~
>gis-dre-mir-6
aacaacaugucacucuaggcuagagcgcuaucugcaguucaaaacuagucuagagcaacauguuguua
a
>5p-matureseq
aacaacaugucacucuaggcu
>5p-matureseq
aacaacaugucacucuaggcua
>3p-matureseq
gucuagagcaacauguuguuaa
>3p-matureseq
ucuagagcaacauguuguuaa
~~~~~~~~~~~~~~~~~~~~~~~~~~~~~~~~~~~~~~~~~~~~~~~~~~~
>gis-dre-mir-7
aacacaccugucuucaaugacugaguucuccuucagguugguuaguuguguuug
>5p-matureseq
aacacaccugucuucaaugacug
```

>3p-matureseq
gguuggguuaguuguguuug
~~~~~~
>gis-dre-mir-8
aacacagggugcacaauagaguucgcuucacuaaugcccucucuaaugccccgugcag
>5p-matureseq
aacacagggugcacaauagagu
>3p-matureseq
ccucucuaaugccccgugcag
~~~~~~
>gis-dre-mir-9
aacauuacucucuuugacacuguauuucauauucacugugucagaucgaguauguuug
>5p-matureseq
aacauuacucucuuugacacugu
>3p-matureseq
ugugucagaucgaguauguuug
~~~~~~
>gis-dre-mir-10
aaccagcagacguugacauccauaguaggaagaaaaaaauacuauggaagucaauggcugcugcuucc
>5p-matureseq
aaccagcagacguugacaucc
>3p-matureseq
aagucaauggcugcugcuucc
~~~~~~
>gis-dre-mir-11
aacgcagagcucuaaaacaucugcgucguggacgcgcugcagcgcauguuuugaagcucugcgcuu
>5p-matureseq
aacgcagagcucuaaaacaucu
>3p-matureseq
gcauguuuugaagcucugcgcuu
~~~~~~
>gis-dre-mir-12
aacuuguccuggaagcuggagguguauagacaaucccuuagcucucaugacaugauuu
>5p-matureseq
aacuuguccuggaagcuggaggu
>3p-matureseq
uuagcucucaugacaugauuu
~~~~~~
>gis-dre-mir-13
aagcaggaacuguugacauccauaguaaacagaggauggaagucaacaguuccugcuaucc
>5p-matureseq
aagcaggaacuguugacaucc
>3p-matureseq
gaagucaacaguuccugcuaucc
~~~~~~
>gis-dre-mir-14
aaggcuuuucaccauuuuggaugaaguaaugguugccccauccaucuggauugaaagguuaug
>5p-matureseq
aaggcuuuucaccauuuuggau
>3p-matureseq
uccaucuggauugaaagguuaug
~~~~~~
>gis-dre-mir-15
aaggcuuuucaccauuuuggaugcaguaaugauugccccauccaucuggauugacaaguuaug

```
>5p-matureseq
aaggcuuuucaccauuuuggau
>3p-matureseq
uccaucuggauugacaaguuaug
~~~~~~~~~~~~~~~~~~~~~~~~~~~~~~~~~~~~~~~~~~~~~~~~~~~
>gis-dre-mir-16
aaggcuuuucaccauuuuggaugcaguaaugguugccccauccaacuggauugaaaaguuaug
>5p-matureseq
aaggcuuuucaccauuuuggau
>3p-matureseq
uccaacuggauugaaaaguuaug
~~~~~~~~~~~~~~~~~~~~~~~~~~~~~~~~~~~~~~~~~~~~~~~~~~~
>gis-dre-mir-17
aaggcuuuucaccauuuuggaugcaguaaugguugccccauccaucuggauugacaaguuaug
>5p-matureseq
aaggcuuuucaccauuuuggau
>3p-matureseq
uccaucuggauugacaaguuaug
~~~~~~~~~~~~~~~~~~~~~~~~~~~~~~~~~~~~~~~~~~~~~~~~~~~
>gis-dre-mir-18
aaggcuuuucaccauuuuggaugcaguaaugguugccccguccaucuggauugaacaguuaug
>5p-matureseq
aaggcuuuucaccauuuuggau
>3p-matureseq
uccaucuggauugaacaguuaug
~~~~~~~~~~~~~~~~~~~~~~~~~~~~~~~~~~~~~~~~~~~~~~~~~~~
>gis-dre-mir-19
aaguuuucugguucuugagucguucguucaucacgugacagaaugacucgaacccgaggacucg
>5p-matureseq
aaguuuucugguucuugagucg
>5p-matureseq
aaguuuucugguucuugagucgu
>5p-matureseq
aaguuuucugguucuugagucguucg
>3p-matureseq
augacucgaacccgaggacucg
~~~~~~~~~~~~~~~~~~~~~~~~~~~~~~~~~~~~~~~~~~~~~~~~~~~
>gis-dre-mir-20
aaguuuucugguucuugagucguuuguucaucacaugacagaaugacucaaacccaaggacucg
>5p-matureseq
aaguuuucugguucuugagucg
>5p-matureseq
aaguuuucugguucuugagucgu
>3p-matureseq
augacucaaacccaaggacucg
~~~~~~~~~~~~~~~~~~~~~~~~~~~~~~~~~~~~~~~~~~~~~~~~~~~
>gis-dre-mir-21
aaugcugaaugccauugaugucuauaguauuuguuuuuccuacuauggaaguuaaugguguuuagcau
ucu
>3p-matureseq
aaguuaaugguguuuagcauucu
>5p-matureseq
aaugcugaaugccauugaugucu
>3p-matureseq
```

aguuaaugguguuuagcauucu
~~~~~~
>gis-dre-mir-22
aaugcugauuuuggaucuugugcccugauaauuaagaaacgcuuugaauaacaugggucugauccucu
gucagcagucu
>5p-matureseq
aaugcugauuuuggaucuugugc
>5p-matureseq
aaugcugauuuuggaucuugugccc
>3p-matureseq
ucugauccucugucagcagucu
~~~~~~
>gis-dre-mir-23
aauguucugugaccauugaaaucgguuuucacagauuucgauagucacagaauauggu
>5p-matureseq
aauguucugugaccauugaaau
>3p-matureseq
uucgauagucacagaauauggu
~~~~~~
>gis-dre-mir-24
aauuauugauaugugggccagauguaaaauguaguauuuggcccagauguuaauaauuga
>5p-matureseq
aauuauugauaugugggccaga
>3p-matureseq
uggcccagauguuaauaauuga
~~~~~~
>gis-dre-mir-25
acacgcccacuaaaugcagagcaacuguacuuucagugggcguaccucacaguggcucugcauuuagu
gggcguguuu
>5p-matureseq
acacgcccacuaaaugcagagc
>3p-matureseq
ucugcauuuagugggcguguuu
~~~~~~
>gis-dre-mir-26
acacugaaaggguauguaaaucaccaacaguuacacaaaaaugaagcucauagugaauaugccuguug
uguag
>5p-matureseq
acacugaaaggguauguaaaucaccaaca
>3p-matureseq
auagugaauaugccuguuguguag
~~~~~~
>gis-dre-mir-27
acacugaaaggguauguaaaucaccaacaguuacacacaaaugaagcucauagugaauaugccuguug
uguag
>5p-matureseq
acacugaaaggguauguaaaucaccaaca
>3p-matureseq
auagugaauaugccuguuguguag
~~~~~~
>gis-dre-mir-28
acagagagucagugauuaggcugcaaaacaaagcugauuagccuagccucugucugucugugu
>5p-matureseq
acagagagucagugauuaggcu
~~~~~~

```
>3p-matureseq
ccuagccucugucugucugugu
~~~~~~~~~~~~~~~~~~~~~~~~~~~~~~~~~~~~~~~~~~~~~~~~~~~
>gis-dre-mir-29
acagccagugacuuccauagcauuuuguuuuuucgaccauggaggucaauggcugcau
>5p-matureseq
acagccagugacuuccauagc
>3p-matureseq
cauggaggucaauggcugcau
~~~~~~~~~~~~~~~~~~~~~~~~~~~~~~~~~~~~~~~~~~~~~~~~~~~
>gis-dre-mir-30
acagcuacuguuaacucucaaaguaaagggcauguaagagugacaguugcuacgg
>5p-matureseq
acagcuacuguuaacucucaaagu
>3p-matureseq
uaagagugacaguugcuacgg
~~~~~~~~~~~~~~~~~~~~~~~~~~~~~~~~~~~~~~~~~~~~~~~~~~~
>gis-dre-mir-31
accgguaaccaucgacuuccauaguaggaaaaacaaaugcuaugaaaguuaaugguuaucgguuu
>5p-matureseq
accgguaaccaucgacuuccau
>3p-matureseq
gaaaguuaaugguuaucgguuu
~~~~~~~~~~~~~~~~~~~~~~~~~~~~~~~~~~~~~~~~~~~~~~~~~~~
>gis-dre-mir-32
acggauagaaucagcggagcugggagcgcucucucuccccgcuccgcugauuuugucagug
>5p-matureseq
acggauagaaucagcggagcugg
>3p-matureseq
cgcuccgcugauuuugucagug
~~~~~~~~~~~~~~~~~~~~~~~~~~~~~~~~~~~~~~~~~~~~~~~~~~~
>gis-dre-mir-33
acuggcacucauugacuucuguaguauuuuuuuguuucuaugaaagucuauugguggcagcca
>5p-matureseq
acuggcacucauugacuucugu
>3p-matureseq
ugaaagucuauugguggcagcca
~~~~~~~~~~~~~~~~~~~~~~~~~~~~~~~~~~~~~~~~~~~~~~~~~~~
>gis-dre-mir-34
acuugaggcaggugugcaugagcggcgccgaggaaacgcugguucugaaggagguggcucgugcagag
gugucgugg
>5p-matureseq
acuugaggcaggugugcaugagcggcgc
>3p-matureseq
ggcucgugcagaggugucgugg
~~~~~~~~~~~~~~~~~~~~~~~~~~~~~~~~~~~~~~~~~~~~~~~~~~~
>gis-dre-mir-35
acuuugcucacgugacuuggguguuucaaauagcuuaagucaugugaucuaggggaa
>5p-matureseq
acuuugcucacgugacuugggu
>3p-matureseq
uaagucaugugaucuaggggaa
~~~~~~~~~~~~~~~~~~~~~~~~~~~~~~~~~~~~~~~~~~~~~~~~~~~
>gis-dre-mir-36
```

agaaaucagauucuccacauacacuuugacuaaaugcuucuguguggagaaucugauuucuag
>5p-matureseq
agaaaucagauucuccacauaca
>3p-matureseq
uguguggagaaucugauuucuag
~~~~~~
>gis-dre-mir-37
agaaugcacaccugaggugacgagauguggcuguuacaccucaggugugcauuauuu
>5p-matureseq
agaaugcacaccugaggugacg
>3p-matureseq
uuacaccucaggugugcauuauuu
~~~~~~
>gis-dre-mir-38
agacaccucuguuaucaaacuaaacaaaacaugagauucauuugcugcucgcuuuguugauaacagag
guguccccu
>5p-matureseq
agacaccucuguuaucaaacu
>3p-matureseq
uguugauaacagagguguccccu
~~~~~~
>gis-dre-mir-39
agacgcugacucugauggguuagucagugagaugaaccugucgggcggcgaaca
>5p-matureseq
agacgcugacucugauggguua
>3p-matureseq
ugaaccugucgggcggcgaaca
~~~~~~
>gis-dre-mir-40
agacuaguagccauugagaucuuuuuuuucuauuauggauguuaguggcuacugguuuca
>5p-matureseq
agacuaguagccauugagaucu
>3p-matureseq
auguuaguggcuacugguuuca
~~~~~~
>gis-dre-mir-41
agacuccaucauggugcgguucagcugauaugaccugggguuucugaacucacacagaaggagcucc
>3p-matureseq
acucacacagaaggagcucc
>5p-matureseq
agacuccaucauggugcgguuc
~~~~~~
>gis-dre-mir-42
agagagaaaagucaauggcuuaaguggcuggagucgcugaugauucucuuggc
>5p-matureseq
agagagaaaagucaauggcuua
>3p-matureseq
agucgcugaugauucucuuggc
~~~~~~
>gis-dre-mir-43
agagagaaaagucaauggcuugaguggcuggaguuacugauaauucuuuuggc
>5p-matureseq
agagagaaaagucaauggcuug
>3p-matureseq
~~~~~~

aguuacugauaauucuuuuggc
~~~~~~
>gis-dre-mir-44
agagagaaaagucaauggcuuggguggcaggagucgcugauaauucucuuggc
>5p-matureseq
agagagaaaagucaauggcuug
>3p-matureseq
agucgcugauaauucucuuggc
>3p-matureseq
gagucgcugauaauucucuuggc
~~~~~~
>gis-dre-mir-45
agagagaaaagucuauggcuuggguggcuggagucacugauaauucucuuggcu
>5p-matureseq
agagagaaaagucuauggcuug
>3p-matureseq
agucacugauaauucucuuggc
>3p-matureseq
gucacugauaauucucuuggcu
>3p-matureseq
ucacugauaauucucuuggcu
~~~~~~
>gis-dre-mir-46
agagagaaaagucuguggcuugggugacuggagucgcugauaauucucuuggc
>5p-matureseq
agagagaaaagucuguggcuug
>3p-matureseq
agucgcugauaauucucuuggc
>3p-matureseq
gagucgcugauaauucucuuggc
~~~~~~
>gis-dre-mir-47
agagagaaaagucuguggcuuggguggcuggagucacuaauauuucucuuggc
>5p-matureseq
agagagaaaagucuguggcuug
>3p-matureseq
agucacuaauauuucucuuggc
~~~~~~
>gis-dre-mir-48
agagagaaaagucuuuggcuugggugacuggagucauugauaauucucuuggc
>5p-matureseq
agagagaaaagucuuuggcuug
>3p-matureseq
agucauugauaauucucuuggc
>3p-matureseq
gucauugauaauucucuuggc
~~~~~~
>gis-dre-mir-49
agagagaaaugucuauggcuuggguggcuggagucgcugaugauuaucuuggc
>5p-matureseq
agagagaaaugucuauggcuug
>3p-matureseq
agucgcugaugauuaucuuggc
~~~~~~
~~~~~~

>gis-dre-mir-50
agagaggaaagucuaugucuugggguggcuggagucauugauaauucucuuggc
>5p-matureseq
agagaggaaagucuaugucuug
>3p-matureseq
agucauugauaauucucuuggc
>3p-matureseq
gucauugauaauucucuuggc
~~~~~~
>gis-dre-mir-51
agagcguaggcuggggccguggagaggcgucugugcacugccuccagcugacagcucccu
>5p-matureseq
agagcguaggcuggggccgugga
>3p-matureseq
cugccuccagcugacagcucccu
~~~~~~
>gis-dre-mir-52
agaggcuguccgagugcugauauuacauuaaugucacgacuaucagcacucggacagccucuuc
>5p-matureseq
agaggcuguccgagugcugaua
>5p-matureseq
gaggcuguccgagugcugaua
>3p-matureseq
ucagcacucggacagccucuuc
~~~~~~
>gis-dre-mir-53
agagggaaaagucuauggcuugggguggcuggagucgcugaugauucucuuggcu
>5p-matureseq
agagggaaaagucuauggcuug
>3p-matureseq
agucgcugaugauucucuuggc
>3p-matureseq
ucgcugaugauucucuuggcu
~~~~~~
>gis-dre-mir-54
agauagaaucagcggagcggggagaacucucucucccagcuccgcugauucugucagu
>5p-matureseq
agauagaaucagcggagcgggg
>3p-matureseq
cagcuccgcugauucugucagu
~~~~~~
>gis-dre-mir-55
agaucuacuuugauuguucagaugacucugcucugcucugauuggucaaauaggucuaca
>5p-matureseq
agaucuacuuugauuguucaga
>3p-matureseq
ugauuggucaaauaggucuac
>3p-matureseq
ugauuggucaaauaggucuaca
~~~~~~
>gis-dre-mir-56
agauucaucacgacugacgguguacgacggaggaguagaucgacggggacuugacccuu
>5p-matureseq
agauucaucacgacugacgguguacga

>3p-matureseq
guagaucgacggggacuugacccuu
~~~~~~
>gis-dre-mir-57
agauuuagcacacuucuggcauauauuaucauguuguuaguagugugcuaagucaug
>5p-matureseq
agauuuagcacacuucuggca
>3p-matureseq
uuaguagugugcuaagucaug
~~~~~~
>gis-dre-mir-58
agcaaucuagccugugcagauguguaguucuccagcgaucugcccaggccagacuacuagu
>5p-matureseq
agcaaucuagccugugcagaug
>3p-matureseq
ucugcccaggccagacuacuagu
~~~~~~
>gis-dre-mir-59
agcagcacaaaguacuauggagagcgucaugauaaaggcucgguacacuuguguuugacc
>5p-matureseq
agcagcacaaaguacuauggagagc
>3p-matureseq
ucgguacacuuguguuugacc
~~~~~~
>gis-dre-mir-60
agccaaccaggcacgugcaguggaguuuacugguucagugggcaugggcucauccgccggacucggga
ccgccggucugcugacugg
>5p-matureseq
agccaaccaggcacgugcaguggagu
>3p-matureseq
ucgggaccgccggucugcugacugg
~~~~~~
>gis-dre-mir-61
agccauuuccagccuggucuuagcuguucagacuggaaaaugaccagccaaauccagcuaaaaccagc
uugaccagccuggacauagcugg
>5p-matureseq
agccauuuccagccuggucuuagc
>3p-matureseq
uugaccagccuggacauagcugg
~~~~~~
>gis-dre-mir-62
agccauuugcugaguccaagauucaguuccuggacucagcaaauggcuca
>5p-matureseq
agccauuugcugaguccaaga
>3p-matureseq
cuggacucagcaaauggcuca
~~~~~~
>gis-dre-mir-63
agccgacucgcagcucucugucuccaaaauggcgcuugugagcgaggagagagaccgugagucgguag
c
>3p-matureseq
agagagaccgugagucgguagc
>5p-matureseq
agccgacucgcagcucucugucu
~~~~~~

>5p-matureseq
agccgacucgcagcucucugucucc
~~~~~~
>gis-dre-mir-64
agcggaagcccuauauugaccauauagucauaaaugaucaauauguggcuuccucu
>5p-matureseq
agcggaagcccuauauugaccau
>3p-matureseq
ugaucaauauguggcuuccucu
~~~~~~
>gis-dre-mir-65
agcggcgucagaagcgauggccgugucucuauccaauaggucgggucgcuaaugacgucacc
>5p-matureseq
agcggcgucagaagcgauggcc
>5p-matureseq
agcggcgucagaagcgauggccg
>3p-matureseq
gucgggucgcuaaugacgucacc
~~~~~~
>gis-dre-mir-66
agcuguauccuauuuagacuuuaccgugcugcaggagagugugaagucuagaugcgauacagaug
>5p-matureseq
agcuguauccuauuuagacuuu
>3p-matureseq
gaagucuagaugcgauacagaug
~~~~~~
>gis-dre-mir-67
aggaugcaggacuccgcucugaauaaugaggaaaagaccaaucagagaggagaaugucugcagccccg
c
>5p-matureseq
aggaugcaggacuccgcucug
>3p-matureseq
gagaggagaaugucugcagccccgc
~~~~~~
>gis-dre-mir-68
aggcuguccgagugcugauagucgugacauuaauguaauaucagcacucggacagccucuuc
>5p-matureseq
aggcuguccgagugcugauagu
>3p-matureseq
ucagcacucggacagccucuuc
~~~~~~
>gis-dre-mir-69
agguuuguacugagacugacuuuaaaguaaagugaugcugaggucuuucucuguaccaaucaag
>5p-matureseq
agguuuguacugagacugacu
>3p-matureseq
ucuuucucuguaccaaucaag
~~~~~~
>gis-dre-mir-70
agucuccauguaucuguguuugcacugggugaagagcgaaaaacugagugccaacacagauacacgga
gacugg
>3p-matureseq
aacacagauacacggagacugg
>5p-matureseq
~~~~~~

agucuccauguaucuguguuug
~~~~~~
>gis-dre-mir-71
agucuccguguaucuguguuugcacugggugaagagcgguaaacugagugcgaacacagauacacgga
gacuga
>3p-matureseq
aacacagauacacggagacuga
>5p-matureseq
agucuccguguaucuguguuug
>5p-matureseq
agucuccguguaucuguguuugc
>5p-matureseq
gucuccguguaucuguguuug
~~~~~~
>gis-dre-mir-72
agucuucguucugcaucgggacuuaccuuguaucagccacgauguaaacucaagaccau
>5p-matureseq
agucuucguucugcaucggga
>3p-matureseq
ccacgauguaaacucaagaccau
~~~~~~
>gis-dre-mir-73
agucuucugguucuugagucguucguucaucaugugacagaccgacucaaacccgaggacucg
>5p-matureseq
agucuucugguucuugagucguuc
>3p-matureseq
ccgacucaaacccgaggacucg
~~~~~~
>gis-dre-mir-74
agugcuguucuggaauguauucagguuugguuucuucugaaucucugagcccagcgcuga
>3p-matureseq
aaucucugagcccagcgcuga
>5p-matureseq
agugcuguucuggaauguauuca
~~~~~~
>gis-dre-mir-75
aguucaggagggagacagcucuggggaaaaagcuguuccucagucugcugguuuuuuuccgggggagc
cu
>5p-matureseq
aguucaggagggagacagcucu
>3p-matureseq
ugcugguuuuuuuccgggggagccu
~~~~~~
>gis-dre-mir-76
aguucuauagcaguguuugguuaucauucagcggaggaaccacacacaacuauagcacugc
>5p-matureseq
aguucuauagcaguguuugguu
>3p-matureseq
ccacacacaacuauagcacugc
~~~~~~
>gis-dre-mir-77
aguuuauuguauggagcgcgucugucagucagcgcuuauacauacuggu
>5p-matureseq
aguuuauuguauggagcgcgucugu
~~~~~~

>3p-matureseq
gucagcgcuuauacauacuggu
~~~~~~
>gis-dre-mir-78
aguuucgguuagugacucaugccuggaauucaggcuggcuuacucucacguugu
>5p-matureseq
aguuucgguuagugacucaugcc
>3p-matureseq
cuggcuuacucucacguugu
~~~~~~
>gis-dre-mir-79
aucgguuugaugaucuucagacgugacaauauauaucaguuugaccaucuucagacgugacuauuggu
uugacgaucuucagacgugacu
>5p-matureseq
aucgguuugaugaucuucagac
>3p-matureseq
uugaccaucuucagacgugacua
>3p-matureseq
uugacgaucuucagacgugacu
>3p-matureseq
uuugaccaucuucagacguga
>3p-matureseq
uuugacgaucuucagacgugacu
~~~~~~
>gis-dre-mir-80
aucuaucagaugauccaucugucaacagauuuaugagagauccacugauggaacagcugauagacgu
>5p-matureseq
aucuaucagaugauccaucuguc
>3p-matureseq
ugauggaacagcugauagacgu
~~~~~~
>gis-dre-mir-81
aucuuuguucuggcuauauugaaggaguauucagaauguagccugaacaaaaccugauug
>5p-matureseq
aucuuuguucuggcuaua
>3p-matureseq
guagccugaacaaaaccugauu
>3p-matureseq
uagccugaacaaaaccugauug
~~~~~~
>gis-dre-mir-82
augaagucaaugcaaagauuggauuaggcaucccauuucaaucuuugcauugccuucaugu
>3p-matureseq
aaucuuugcauugccuucaugu
>3p-matureseq
aucuuugcauugccuucaugu
>5p-matureseq
augaagucaaugcaaagauugg
>5p-matureseq
augaagucaaugcaaagauugga
~~~~~~
>gis-dre-mir-83
augaguguacuguuggcugcugggaacacuucgugacgacugaaaccaaaggaaaagaggaacuggcu
uuguuuc

>3p-matureseq
aggaaaagaggaacuggcuuuguuuc
>5p-matureseq
augaguguacuguuggcugcu
~~~~~~
>gis-dre-mir-84
augauacacagcagcaucauggguaauguaguuuugcaccaugaaugcugcuguauaucauu
>3p-matureseq
augaaugcugcuguauaucauu
>5p-matureseq
augauacacagcagcaucauggg
~~~~~~
>gis-dre-mir-85
augauguccauuucugcugcgcuugugguugucaggagugcaagaauaggaccuucaggc
>5p-matureseq
augauguccauuucugcugcg
>3p-matureseq
caagaauaggaccuucaggc
~~~~~~
>gis-dre-mir-86
auucacuauugacugucccuuguuccaaaaucuuuacgagggagagucaauagugaacaca
>3p-matureseq
agggagagucaauagugaacaca
>5p-matureseq
auucacuauugacugucccuug
~~~~~~
>gis-dre-mir-87
auuccggcgcggaugcggcagcgucggcucgcuuacggccgccgcaucuggccggaaucg
>5p-matureseq
auuccggcgcggaugcggcag
>5p-matureseq
auuccggcgcggaugcggcagcg
>3p-matureseq
cgccgcaucuggccggaaucg
~~~~~~
>gis-dre-mir-88
auuccggcgcggaugcggcagcgucggcucgcuuacggcugccgcaucuggcccgaaucg
>5p-matureseq
auuccggcgcggaugcggcag
>5p-matureseq
auuccggcgcggaugcggcagcg
>3p-matureseq
gccgcaucuggcccgaaucg
~~~~~~
>gis-dre-mir-89
auuccggcgcggaugcggcagcguuggcucgcuuacggccgccgcaucuggcccggaauuga
>5p-matureseq
auuccggcgcggaugcggcag
>5p-matureseq
auuccggcgcggaugcggcagcg
>3p-matureseq
cgccgcaucuggcccggaauuga
~~~~~~
>gis-dre-mir-90
~~~~~~

caaucagcugaucugucugcaagcugagauaugagugauccgcugauggauugacugauuggc
>5p-matureseq
caaucagcugaucugucugca
>3p-matureseq
cugauggauugacugauuggc
~~~~~~
>gis-dre-mir-91
cacauuugggcgaguucuuugaucaucuugaauucaugguacuccuuggauguugac
>3p-matureseq
augguacuccuuggauguugac
>5p-matureseq
cacauuugggcgaguucuuuga
~~~~~~
>gis-dre-mir-92
cacgaccagcccgagccgcgguggaauggagcagcgcagcccggacucggucaagg
>5p-matureseq
cacgaccagcccgagccgcggugga
>3p-matureseq
cgcagcccggacucggucaagg
~~~~~~
>gis-dre-mir-93
cacgcccacuaaaugcagagccacugugagguacgcccacugaaaguacaguugcucugcauuuagug
ggcguguuu
>5p-matureseq
cacgcccacuaaaugcagagcca
>3p-matureseq
ucugcauuuagugggcguguuu
~~~~~~
>gis-dre-mir-94
cagaggaagaccgacaggugaagcacacaccgcaccugugcugauugauugaucaccugugcugaugu
cugcuucuccu
>5p-matureseq
agaggaagaccgacaggug
>5p-matureseq
cagaggaagaccgacaggug
>3p-matureseq
ccugugcugaugucugcuucucc
>3p-matureseq
ccugugcugaugucugcuucuccu
~~~~~~
>gis-dre-mir-95
cagcagauucuuggucuucagagucugaauuccuucauucugagucuugagaaucugcaga
>5p-matureseq
cagcagauucuuggucuucaga
>3p-matureseq
ucugagucuugagaaucugcaga
~~~~~~
>gis-dre-mir-96
cagccccggucuugaaucuggucccagacuaaacauuugggcugaucagugacugcggcugcu
>5p-matureseq
cagccccggucuugaaucuggucccc
>3p-matureseq
gcugaucagugacugcggcugcu
~~~~~~
~~~~~~

>gis-dre-mir-97
cagcguccucuccaagccugacugacaauaauuggacagaggcugaagacggggcgcugc
>3p-matureseq
agaggcugaagacggggcgcugc
>5p-matureseq
cagcguccucuccaagccugacu
~~~~~~
>gis-dre-mir-98
caggacauccaauccgacagaccuugaguuagauucugaucagugggauuuggaggccaggc
>5p-matureseq
caggacauccaauccgacagacc
>3p-matureseq
ucagugggauuuggaggccaggc
~~~~~~
>gis-dre-mir-99
cagucucucuggaucagugguuuucuuacagcagcgucucagaucugugacagacagga
>5p-matureseq
cagucucucuggaucaguggu
>3p-matureseq
ucagaucugugacagacagga
~~~~~~
>gis-dre-mir-100
caguuccccguccuguugguguuguaauuuuagucuuaacaaagugggucggagagcuguu
>3p-matureseq
caaagugggucggagagcuguu
>5p-matureseq
caguuccccguccuguugguguu
~~~~~~
>gis-dre-mir-101
caguuugguucgguaggccuuuugacaguggaaacagccauaaaagcuuaccaaaccguaccgua
>3p-matureseq
agcuuaccaaaccguaccgua
>5p-matureseq
caguuugguucgguaggccuuu
~~~~~~
>gis-dre-mir-102
caguuugguucgguaggccuuuugacaguggaaacggccauaaaagcguaccaaaccgaaccaua
>3p-matureseq
aaaagcguaccaaaccgaacc
>3p-matureseq
agcguaccaaaccgaaccaua
>3p-matureseq
auaaaagcguaccaaaccgaacc
>5p-matureseq
caguuugguucgguaggccuuu
>3p-matureseq
uaaaagcguaccaaaccgaacc
~~~~~~
>gis-dre-mir-103
caucaaaugucaucgcgaucaucauauguagaaagugaucgcuaugacauuugaugacg
>3p-matureseq
aucgcuaugacauuugaugacg
>5p-matureseq
caucaaaugucaucgcgaucau

~~~~~~
>gis-dre-mir-104
caucuggucuaguugcaggaguucaaauauuucaacggacccgcagcucaaaucagaucu
>3p-matureseq
acccgcagcucaaaucagaucu
>5p-matureseq
caucuggucuaguugcaggaguu
~~~~~~
>gis-dre-mir-105
caugaugaaugccuugaucagaugcugacugugacauuaagugacggcuugaucaggauucaucaucg
cc
>5p-matureseq
caugaugaaugccuugaucaga
>3p-matureseq
cuugaucaggauucaucaucgcc
~~~~~~
>gis-dre-mir-106
caugcacugucaacccugacaccuuauauagacagguguaugcguuuucaggucauguc
>5p-matureseq
caugcacugucaacccugacac
>3p-matureseq
uguaugcguuuucaggucauguc
~~~~~~
>gis-dre-mir-107
cauuagaggucauuuuuacuguuuguaucacugcuuuaaaaguguccuccagagc
>5p-matureseq
cauuagaggucauuuuuacugu
>3p-matureseq
uuuaaaaguguccuccagagc
~~~~~~
>gis-dre-mir-108
ccaaaucguauguucacugugaauacuguaaaggccuguuuguucgcaaggagcuguggcguc
>5p-matureseq
ccaaaucguauguucacugugaauacugu
>3p-matureseq
uuuguucgcaaggagcuguggcguc
~~~~~~
>gis-dre-mir-109
ccaaucacuugagucuggguuuuugaucucagcugcauccagcagguguuuucggagcagacuucgac
uugauugggu
>5p-matureseq
ccaaucacuugagucuggguuuu
>3p-matureseq
gcagacuucgacuugauugggu
~~~~~~
>gis-dre-mir-110
ccaccacugcaauuccaucaugaugaaaaagaagucugagaaaugcuguaauuggugua
>5p-matureseq
ccaccacugcaauuccaucaugaugaaaaa
>3p-matureseq
ucugagaaaugcuguaauuggugua
~~~~~~
>gis-dre-mir-111
ccaguuccacaauuugccauauucugugcauacuugaaacauaaaauauggcaaaauguggaacuagc

>5p-matureseq
ccaguuccacaauuugccauau
>3p-matureseq
uauggcaaaauguggaacuagc
~~~~~~
>gis-dre-mir-112
ccauccacaucaagguucgacaugugcacaacaugucgaaccuugauguggaugggcu
>5p-matureseq
ccauccacaucaagguucgaca
>3p-matureseq
ucgaaccuugauguggaugggcu
~~~~~~
>gis-dre-mir-113
ccauccacaucaagguucgacauguugugcacaugucgaaccuugauguggaugggcu
>5p-matureseq
ccauccacaucaagguucgaca
>3p-matureseq
ucgaaccuugauguggaugggcu
~~~~~~
>gis-dre-mir-114
ccauuugggcagagcugagcuccagugaggggcucaugcucccccuggcuggagcgcagcucugcuca
aauggcu
>3p-matureseq
agcgcagcucugcucaaauggcu
>5p-matureseq
ccauuugggcagagcugagcu
>5p-matureseq
ccauuugggcagagcugagcucc
>3p-matureseq
cgcagcucugcucaaauggcu
~~~~~~
>gis-dre-mir-115
cccagaauucaaggaccaaccaucuucucaacgcuugauguggucccugccuucuggacg
>5p-matureseq
cccagaauucaaggaccaaccau
>3p-matureseq
ugugguccc ugccuucuggacg
~~~~~~
>gis-dre-mir-116
cccccgcauguccuuugagcccccgcaucugucugcugugcucagagugguugugcgggugu
>5p-matureseq
cccccgcauguccuuugagcccc
>3p-matureseq
gcucagagugguugugcgggugu
~~~~~~
>gis-dre-mir-117
ccgagcuugugcagauaguggaaugguaccaacaagagauaguugggcucaccuccacacacagcuug
ggc
>5p-matureseq
ccgagcuugugcagauaguggaauggua
>3p-matureseq
ucaccuccacacacagcuugggc
~~~~~~
>gis-dre-mir-118
~~~~~~

ccgagcuugugcgggagguggaacgauacugacuagggcucaccuccacacauagcuuggg
>5p-matureseq
ccgagcuugugcgggaggugga
>3p-matureseq
ucaccuccacacauagcuuggg
~~~~~~
>gis-dre-mir-119
ccgccccgucucugcuaccucauaauaaagucauaaugugagucggcggaggcagggugacg
>5p-matureseq
ccgccccgucucugcuaccu
>5p-matureseq
ccgccccgucucugcuaccuca
>3p-matureseq
gucggcggaggcagggugacg
~~~~~~
>gis-dre-mir-120
ccggaauauagaguuggauugggcucucagaguuggguaauccauggcaucaguagagaagccacaac
cgauuguuuaauuuuugug
>3p-matureseq
ccacaaccgauuguuuaauuuuugug
>5p-matureseq
ccggaauauagaguuggauugggcu
~~~~~~
>gis-dre-mir-121
ccgugccugauaaacagggacucugcuuuauagcgaucuaaaaagcgccugauaucagcgcacuggca
g
>5p-matureseq
ccgugccugauaaacagggacu
>3p-matureseq
cgccugauaucagcgcacuggcag
~~~~~~
>gis-dre-mir-122
ccguggagugaucaaggaggacgagauuuacacucucccuccuucucccaccaugagg
>3p-matureseq
cccuccuucucccaccaugagg
>5p-matureseq
ccguggagugaucaaggaggacg
~~~~~~
>gis-dre-mir-123
ccuagaaaaucuggaaaagugcuggaguuuugacaaggcauuuuccaggcuuucuaggac
>3p-matureseq
cauuuuccaggcuuucuaggac
>5p-matureseq
ccuagaaaaucuggaaaagugcu
~~~~~~
>gis-dre-mir-124
ccucgagcccagugcuuccuccuguuggcagagcgaguggggagccugagcucgguggau
>5p-matureseq
ccucgagcccagugcuuccuccu
>3p-matureseq
uggggagccugagcucgguggau
~~~~~~
>gis-dre-mir-125
cgaaccgacagaaaacacaugugaaacacaacauacguguguuuucugucgguucguu
~~~~~~

>5p-matureseq
cgaaccgacagaaaacacaugu
>3p-matureseq
guguguuuucugucgguucguu
~~~~~~
>gis-dre-mir-126
cggccgauuggccggccgcccggucgaucguucagucguucggccggccagucggacaga
>5p-matureseq
cggccgauuggccggccgcccg
>3p-matureseq
uucggccggccagucggacaga
~~~~~~
>gis-dre-mir-127
cggcugugcauggaacucuggagacggcacuggccauuuagggaacucuggacaccuga
>5p-matureseq
cggcugugcauggaacucugga
>3p-matureseq
uagggaacucuggacaccuga
~~~~~~
>gis-dre-mir-128
cgguuugguacgcuucuguggccguuuccacugucaauaaguguaccaaaccaaac
>3p-matureseq
caauaaguguaccaaaccaaac
>5p-matureseq
cgguuugguacgcuucugug
~~~~~~
>gis-dre-mir-129
cgucuacagauacugcuggagagauagaguaugaaaacuccuccacugucucugucggcguc
>5p-matureseq
cgucuacagauacugcuggagag
>3p-matureseq
cuccacugucucugucggcguc
~~~~~~
>gis-dre-mir-130
cgucuggcauuggguuugcagcaaacaaaugcaauggcggacucc
>3p-matureseq
aacaaaugcaauggcggacucc
>5p-matureseq
cgucuggcauuggguuugcagc
~~~~~~
>gis-dre-mir-131
cgugggauugaggguagaagagaacugauuauuaacucuccucacauacaaucccugccu
>5p-matureseq
cgugggauugaggguagaaga
>5p-matureseq
cgugggauugaggguagaagaga
>3p-matureseq
uccucacauacaaucccugccu
~~~~~~
>gis-dre-mir-132
cuacuggccuccauuacuccaguauugaugucuucuggagacguggaggucgguggau
>5p-matureseq
cuacuggccuccauuacucca
>3p-matureseq
~~~~~~

ggagacguggaggucgguggau
~~~~~~
>gis-dre-mir-133
cuccacugucucugucggcgucucuucuuucucuccgcucgacggaguguguauuu
>3p-matureseq
cgcucgacggaguguguauuu
>5p-matureseq
cuccacugucucugucggcguc
~~~~~~
>gis-dre-mir-134
cucgagcccagaguucccucccguugcaaggugggaggggaguuugagcucaggu
>5p-matureseq
cucgagcccagaguucccucccg
>3p-matureseq
ggaggggaguuugagcucaggu
~~~~~~
>gis-dre-mir-135
cucggaguagauccuugagagaagucagcugagaugacucagagaucuguuucgaauu
>3p-matureseq
acucagagaucuguuucgaauu
>5p-matureseq
cucggaguagauccuugagaga
~~~~~~
>gis-dre-mir-136
cucugacagaaucagcggagcgggggagagagagcgcuccccgcaccgcugauucuauccguac
>3p-matureseq
caccgcugauucuauccguac
>5p-matureseq
cucugacagaaucagcggagc
>5p-matureseq
cucugacagaaucagcggagcg
~~~~~~
>gis-dre-mir-137
cucuuccuucuacgucauuaaaccgcggagaagcaccacauugucgaaaguuuaaugauguacugaac
ggaaugccu
>5p-matureseq
cucuuccuucuacgucauuaaaccgcg
>3p-matureseq
uaaugauguacugaacggaaugccu
~~~~~~
>gis-dre-mir-138
cugaagcaaaaacucgggccuggauagaguucaggaaggccagacugcauuguugacgcg
>3p-matureseq
aggccagacugcauuguugacgcg
>5p-matureseq
cugaagcaaaaacucgggccugg
~~~~~~
>gis-dre-mir-139
cugaagcccuugugaauugggcauuauaagguaaagcccaauucacacugggcuucagc
>3p-matureseq
ccaauucacacugggcuucagc
>5p-matureseq
cugaagcccuugugaauugggc
~~~~~~

>gis-dre-mir-140
cugaggccucggcugcaggucugauugcucuguuaacucuucaccaugcagccgagaccaaga
>5p-matureseq
cugaggccucggcugcaggucuga
>3p-matureseq
ucaccaugcagccgagaccaaga
~~~~~~
>gis-dre-mir-141
cugaugaagucacucuaugacccgcgggcucugugucucgggauagguugauuucauugcau
>5p-matureseq
cugaugaagucacucuaugacc
>5p-matureseq
cugaugaagucacucuaugaccc
>5p-matureseq
cugaugaagucacucuaugacccg
>3p-matureseq
ggauagguugauuucauugcau
~~~~~~
>gis-dre-mir-142
cugccauccagcgcucugaaccugcggcuuucuggcgggcagagcgcaggugggcgaac
>5p-matureseq
cugccauccagcgcucugaaccu
>3p-matureseq
gcagagcgcaggugggcgaac
~~~~~~
>gis-dre-mir-143
cuggaacacaugcguuagcacagcaacacaaugcuaaucgucaacagucuagu
>3p-matureseq
acaaugcuaaucgucaacagucuagu
>5p-matureseq
cuggaacacaugcguuagcacagca
~~~~~~
>gis-dre-mir-144
cuggagaucagcagaauggauucaguauugguaaaccucaacauucagcugaucucugggu
>3p-matureseq
caacauucagcugaucucugggu
>5p-matureseq
cuggagaucagcagaauggauuc
~~~~~~
>gis-dre-mir-145
cuggagauccgcugaauugauucgauaaugguaaaacuuaacauucagcugaucucugggu
>5p-matureseq
cuggagauccgcugaauugauuc
>3p-matureseq
uaacauucagcugaucucugggu
~~~~~~
>gis-dre-mir-146
cugguuuugcuggucuaggugcuggucaugcugguuuaaaugcauugaccagcuagaccagcauuaaa
accagcaagaccagcu
>3p-matureseq
auuaaaaccagcaagaccagcu
>5p-matureseq
cugguuuugcuggucuaggugc
~~~~~~
~~~~~~

>gis-dre-mir-147
cuguacgguucaguuugguaugcauuuauggccguuuccacugucaaaagacauaccgaaccguacug
uaccgua
>5p-matureseq
cuguacgguucaguuugguaug
>3p-matureseq
uaccgaaccguacuguaccgua
~~~~~~
>gis-dre-mir-148
cuguacgguucaguuugguauguuuccacugucaaaaggcguaccaaaccgaaccguaucaua
>5p-matureseq
cuguacgguucaguuugguaug
>3p-matureseq
uaccaaaccgaaccguaucaua
~~~~~~
>gis-dre-mir-149
cugugcuugagucaaaaguuuguacauccagcuaaggaugaaagcaggggc
>5p-matureseq
cugugcuugagucaaaaguuu
>3p-matureseq
gcuaaggaugaaagcaggggc
~~~~~~
>gis-dre-mir-150
cuguggucagcgcugggucguugcagugugaagauauagccgacgacacuguucugauuggcugu
>3p-matureseq
acgacacuguucugauuggcugu
>5p-matureseq
cuguggucagcgcugggucgu
~~~~~~
>gis-dre-mir-151
cuuacaauuaaaggauauuucuugagccgaaacuagcagaaauaucucuuaauuguuuggu
>3p-matureseq
aaauaucucuuaauuguuuggu
>5p-matureseq
cuuacaauuaaaggauauuucu
>5p-matureseq
uuacaauuaaaggauauuucuu
~~~~~~
>gis-dre-mir-152
cuucacucgucuguguucagcucuggaugaugugaugcugaacacggcccugagugaggga
>5p-matureseq
cuucacucgucuguguucagc
>3p-matureseq
ugaacacggcccugagugaggga
~~~~~~
>gis-dre-mir-153
cuugggaugaucuggcucgcuggaguacaaaguguuguggaagacuuaggugucagagggccauucau
uccuuacc
>3p-matureseq
cagagggccauucauuccuuacc
>5p-matureseq
cuugggaugaucuggcucgcugga
~~~~~~
>gis-dre-mir-154
~~~~~~

cuuguaaccauugaguucuauaguauuuguuuguccuacuaugaaaaucacugguuacaggcu
>5p-matureseq
cuuguaaccauugaguucuaua
>3p-matureseq
ugaaaaucacugguuacaggcu
~~~~~~
>gis-dre-mir-155
gaaaccaagcaugggugugccugaauccuccaauggcgacccauccuugguuucuga
>5p-matureseq
gaaaccaagcauggguguggcc
>5p-matureseq
gaaaccaagcauggguguggccu
>3p-matureseq
gcgacccauccuugguuucuga
~~~~~~
>gis-dre-mir-156
gaaagcugauuacugugacugcagugcacacuguaaaagcugcagucacaguaaucagcuuuu
>5p-matureseq
aaagcugauuacugugacugc
>5p-matureseq
gaaagcugauuacugugacugc
>3p-matureseq
gcagucacaguaaucagcuuuu
>3p-matureseq
ugcagucacaguaaucagcuuu
~~~~~~
>gis-dre-mir-157
gaaagggugccgaaaaagugguacgguacgauuugguucgguacgccuuuuga
>5p-matureseq
gaaagggugccgaaaaagug
>3p-matureseq
uuugguucgguacgccuuuuga
~~~~~~
>gis-dre-mir-158
gaaaguggacaaaagaaacacauucaaaugccuggugucaaccguaaugugauucauuuguccacuug
uga
>5p-matureseq
gaaaguggacaaaagaaacaca
>3p-matureseq
ugauucauuuguccacuuguga
~~~~~~
>gis-dre-mir-159
gaaaguggacaaaagaaacacauuuaauugcuagguuugaaugguaaugugugucuuuuguccacuug
uga
>5p-matureseq
gaaaguggacaaaagaaacaca
>3p-matureseq
ugugucuuuuguccacuuguga
~~~~~~
>gis-dre-mir-160
gaaauuagcuauugacauaaaaaaaauucuacuauggauaucaguggcuaguuucca
>5p-matureseq
gaaauuagcuauugacauaaa
>3p-matureseq

gauaucaguggcuaguuucca
~~~~~~
>gis-dre-mir-161
gaagcauccuuuguccucugcccccgaagcccgagcccuaaggcagagcggccggaugugccu
>5p-matureseq
gaagcauccuuuguccucugccccc
>3p-matureseq
uaaggcagagcggccggaugugccu
~~~~~~
>gis-dre-mir-162
gaagcauccuuuguccucugcccccggagcccgagcccuaaggcagagcggccggaugugccu
>5p-matureseq
gaagcauccuuuguccucugccccc
>5p-matureseq
gaagcauccuuuguccucugcccccgg
>3p-matureseq
uaaggcagagcggccggaugugccu
~~~~~~
>gis-dre-mir-163
gaagguagugagcugugccuuaaaacuggacuggguucuucaauagagccgggagaaguguccgaggu
gaugccacgccggacu
>5p-matureseq
gaagguagugagcugugccuuaaaac
>3p-matureseq
uccgaggugaugccacgccggacu
~~~~~~
>gis-dre-mir-164
gacaucugcacacugcuguguuauugauucagcacagaaguacacagacgucug
>3p-matureseq
cacagaaguacacagacgucug
>5p-matureseq
gacaucugcacacugcugugu
~~~~~~
>gis-dre-mir-165
gaccguccggcugcccgaacgaacaaacgaccgaccgggcggccggcagaucgc
>3p-matureseq
accgggcggccggcagaucgc
>5p-matureseq
gaccguccggcugcccgaacg
~~~~~~
>gis-dre-mir-166
gacucugcaugugugugcguguuuauuuuagcccacacugcaaccauaugcaggaucugc
>3p-matureseq
cugcaaccauaugcaggaucu
>5p-matureseq
gacucugcaugugugugcgugu
>3p-matureseq
ugcaaccauaugcaggaucugc
~~~~~~
>gis-dre-mir-167
gagaagcagacaucagcacaggugaucaaucaaucagcacaggugcggugugugcuucacc
>3p-matureseq
caggugcggugugugcuucacc
>5p-matureseq
~~~~~~

gagaagcagacaucagcacaggu
~~~~~~
>gis-dre-mir-168
gagaauaaugcacacaugagcaacuuaaccaacucaggugugcauuauuuccu
>3p-matureseq
acucaggugugcauuauuuccu
>5p-matureseq
gagaauaaugcacacaugagca
~~~~~~
>gis-dre-mir-169
gagaaucaucagacuaggcaauguuuuuccaaugaucuguugucuaauuuuggugagccuguucug
>5p-matureseq
gagaaucaucagacuaggcaau
>3p-matureseq
ucuaauuuuggugagccuguucug
~~~~~~
>gis-dre-mir-170
gaggcgcggguggcgcggguaguuguggggacgccgcucucuauauucugccacccuagacgcggaua
uaauug
>5p-matureseq
gaggcgcggguggcgcggguag
>3p-matureseq
gccacccuagacgcggauauaauug
~~~~~~
>gis-dre-mir-171
gaguucugagguuuuuguauuugucaucauuaaucacauacgcaagugcaaauccaucagaucugu
>3p-matureseq
agugcaaauccaucagaucugu
>5p-matureseq
gaguucugagguuuuuguauuu
>5p-matureseq
gaguucugagguuuuuguauuug
>5p-matureseq
gaguucugagguuuuuguauuugu
~~~~~~
>gis-dre-mir-172
gaugaggcugaaggccacugagcaugugcaguaagucuccugcagcuacuucaucuu
>5p-matureseq
gaugaggcugaaggccacu
>3p-matureseq
ucuccugcagcuacuucaucuu
~~~~~~
>gis-dre-mir-173
gcacaauacaggcauuuggaugcuggcugcucucaaagcuucccaugcucguagaaugug
>5p-matureseq
gcacaauacaggcauuuggaug
>3p-matureseq
uucccaugcucguagaaugug
~~~~~~
>gis-dre-mir-174
gcacccaucgacuuucauaguauuuguuuucccuacuauugaagucgaugggugcca
>3p-matureseq
cuauugaagucgaugggugcca
>5p-matureseq
~~~~~~

gcacccaucgacuuucauagua
~~~~~~
>gis-dre-mir-175
gcacucugugguucuccaguggcgcauuguuaccuccccaugggccuggcgaucacagggcgaga
>5p-matureseq
gcacucugugguucuccaguggc
>3p-matureseq
ggccuggcgaucacagggcgaga
~~~~~~
>gis-dre-mir-176
gcagacuucgacuugauugggucuuuugauugaggauggagacccaguggguaagucugaga
>3p-matureseq
ccaguggguaagucugaga
>5p-matureseq
gcagacuucgacuugauugggu
~~~~~~
>gis-dre-mir-177
gcaguagugucugauucugucauuucauaagcaugucugaaucugauguuauugcau
>5p-matureseq
gcaguagugucugauucuguca
>3p-matureseq
ucugaaucugauguuauugcau
~~~~~~
>gis-dre-mir-178
gccagcuccgggccgcagcgcacauuacucucgggcugauuccggcgcggaugcggcag
>3p-matureseq
auuccggcgcggaugcggcag
>5p-matureseq
gccagcuccgggccgcagcgc
~~~~~~
>gis-dre-mir-179
gccagggaccucuuagguggagggcaaauggcccucuggggcccagacugcaaguccaaggagaguuu
ggccc
>5p-matureseq
gccagggaccucuuagguggagggcaaa
>3p-matureseq
ugcaaguccaaggagaguuuggccc
~~~~~~
>gis-dre-mir-180
gccaucugacagguccaguucagugucacuguugaauuauagagcgguggaaaaaaacguagauauac
cagaucggcug
>3p-matureseq
aacguagauauaccagaucggcug
>5p-matureseq
gccaucugacagguccaguuca
~~~~~~
>gis-dre-mir-181
gccugccucucugugucucaaacacuucuaacugugucugagacacucagagggugg
>5p-matureseq
gccugccucucugugucucaaaca
>3p-matureseq
ucugagacacucagagggugg
~~~~~~
>gis-dre-mir-182

gcgucaaaaagcaauuuucagaaauucccuguaaucgauuucugacaugucuuuugaugcca
>5p-matureseq
gcgucaaaaagcaauuuucagaaa
>3p-matureseq
ucugacaugucuuuugaugcca
~~~~~~
>gis-dre-mir-183
gcgugauaacucuuggcgacagagugucugcuucuguccaaugguuguacgcuga
>5p-matureseq
gcgugauaacucuuggcgacagagu
>3p-matureseq
uguccaaugguuguacgcuga
~~~~~~
>gis-dre-mir-184
gcguuggaccgguggggguuacauuaacaauuaccaauguaacgccgccgguccuacgcca
>5p-matureseq
cguuggaccgguggggguuaca
>5p-matureseq
gcguuggaccgguggggguuaca
>3p-matureseq
uaacgccgccgguccuacgcc
>3p-matureseq
uaacgccgccgguccuacgcca
~~~~~~
>gis-dre-mir-185
gcuaauguuaugagaccaucugaaugcuguggcauaugguccca ugauguuugcua
>5p-matureseq
gcuaauguuaugagaccaucu
>3p-matureseq
uaugguccca ugauguuugcua
~~~~~~
>gis-dre-mir-186
gcuagugugaauaaggcauaagaaucucgucuaacaaacgucgcuuucuaaccucauucacacuacga
>3p-matureseq
cuaaccucauucacacuacga
>5p-matureseq
gcuagugugaauaaggcauaag
~~~~~~
>gis-dre-mir-187
gcugagacuugggccagacaugguccauguuuagucaaugucuggcaucaagacuugguccc
>5p-matureseq
gcugagacuugggccagaca
>3p-matureseq
ucuggcaucaagacuugguccc
~~~~~~
>gis-dre-mir-188
gcugccacaugggccacaggagugugccauuccacgcccuucgggacaucucggugggggaggagcua
uugugggauuaugguguca gacgg
>5p-matureseq
gcugccacaugggccacagg
>3p-matureseq
ugugggauuaugguguca gacgg
~~~~~~
>gis-dre-mir-189
~~~~~~

gcugcuggacuccggaugcuuguggacucacugcccgauuuccggccagaugcggcggccg
>5p-matureseq
gcugcuggacuccggaugcu
>3p-matureseq
uuuccggccagaugcggcggccg
~~~~~~
>gis-dre-mir-190
gcuuuucaccauguuggaugcaguaagguugccccauccaucuggauugaaaaguu
>3p-matureseq
auccaucuggauugaaaaguu
>5p-matureseq
gcuuuucaccauguuggaugc
~~~~~~
>gis-dre-mir-191
ggaagaagaggcguuguuagugcugagaaaaagacaacaacaauuccucuucuuucuc
>3p-matureseq
acaacaauuccucuucuuucuc
>5p-matureseq
ggaagaagaggcguuguuagug
~~~~~~
>gis-dre-mir-192
ggauagaaucagccgagcggggagcgcucucucuacccgcuccgcugauucugucagu
>3p-matureseq
ccgcuccgcugauucugucagu
>5p-matureseq
ggauagaaucagccgagcggg
>5p-matureseq
ggauagaaucagccgagcgggg
~~~~~~
>gis-dre-mir-193
ggauagaaucagccgagcggggagcgcucucucuccccgcuccgcugauucugucaga
>3p-matureseq
ccgcuccgcugauucugucaga
>5p-matureseq
ggauagaaucagccgagcggg
>5p-matureseq
ggauagaaucagccgagcgggg
~~~~~~
>gis-dre-mir-194
ggauagaaucagcggagcggggagcgcucucucucaccgcuccgcugaaucugucagu
>3p-matureseq
ccgcuccgcugaaucugucagu
>5p-matureseq
ggauagaaucagcggagcgggg
~~~~~~
>gis-dre-mir-195
ggauagaaucagcggagcggggagcgcucucucuccccgcuccgcagauucugucagu
>3p-matureseq
ccgcuccgcagauucugucagu
>5p-matureseq
ggauagaaucagcggagcgggg
~~~~~~
>gis-dre-mir-196
ggauagaaucagcggagcggggagcgcucucucuccccucuccgcugauucuguca
~~~~~~

>3p-matureseq
cccucuccgcugauucuguca
>5p-matureseq
ggauagaaucagcggagcgggg
~~~~~~
>gis-dre-mir-197
ggauagaaucagcggagcggggagcgcucuuucuccccgcucugcugauuuugucagu
>3p-matureseq
ccgcucugcugauuuugucagu
>5p-matureseq
ggauagaaucagcggagcgggg
~~~~~~
>gis-dre-mir-198
ggauagaaucagcugagcgggaaacgcucucucuccccgcuccgcugauucugucagu
>3p-matureseq
ccgcuccgcugauucugucagu
>5p-matureseq
ggauagaaucagcugagcggg
>5p-matureseq
ggauagaaucagcugagcggga
~~~~~~
>gis-dre-mir-199
ggcuaaauaacuuguagaagauggacagagagauuauucuacaagugauuuagcugu
>5p-matureseq
ggcuaaauaacuuguagaaga
>3p-matureseq
uucuacaagugauuuagcugu
~~~~~~
>gis-dre-mir-200
ggcucgcaggaaggccugcugcuuugacgcuguuccucgugacuagagaggagaccuugccugcgggc
uuu
>3p-matureseq
ggagaccuugccugcgggcuuu
>5p-matureseq
ggcucgcaggaaggccugcug
~~~~~~
>gis-dre-mir-201
gggagcaguguuaauuucgucaaugcaaauaagaaugagaaauugucauugcucauga
>3p-matureseq
gagaaauugucauugcucauga
>5p-matureseq
gggagcaguguuaauuucguca
~~~~~~
>gis-dre-mir-202
ggggagaacauguaaacucugcauagaaacgacauuuugugcaguuuacuuguucuuuucgu
>3p-matureseq
caguuuacuuguucuuuucgu
>3p-matureseq
gcaguuuacuuguucuuuucgu
>5p-matureseq
ggagaacauguaaacucugcau
>5p-matureseq
ggggagaacauguaaacucugca
>5p-matureseq

ggggagaacauguaaacucugcaua
>3p-matureseq
ugcaguuuacuuguucuuuucgu
~~~~~~
>gis-dre-mir-203
gguaaccauugacuuccauaguuagaaaaacaaauacuauugagaucaaugguugcagg
>5p-matureseq
gguaaccauugacuuccauagu
>3p-matureseq
uauugagaucaaugguugcagg
~~~~~~
>gis-dre-mir-204
gguagcacuguugcuuaggaucagcagguucuuuaaacgcuuccucucagcaacaacugccc
>5p-matureseq
gguagcacuguugcuuaggauc
>3p-matureseq
uuccucucagcaacaacugccc
~~~~~~
>gis-dre-mir-205
ggugcaaaguuaaacgugcaugcugugugugucuaugugcgugaacuuuguagcga
>5p-matureseq
ggugcaaaguuaaacgugcaug
>3p-matureseq
ugugcgugaacuuuguagcga
~~~~~~
>gis-dre-mir-206
ggugcagcagacuccucuccucucagauaucugugcuggagcugaggagcugaugcucugc
>3p-matureseq
agcugaggagcugaugcucugc
>5p-matureseq
ggugcagcagacuccucuccucu
~~~~~~
>gis-dre-mir-207
ggugcgcaacuucacggggacucucugaugcacgagaaagagcucguaaaguagcgcagggc
>3p-matureseq
gagcucguaaaguagcgcagggc
>5p-matureseq
ggugcgcaacuucacggggacu
~~~~~~
>gis-dre-mir-208
gguuugguacgcuuuuauggccguuuccacugucaaaaggccuaccgaaccaa
>5p-matureseq
gguuugguacgcuuuuauggcc
>3p-matureseq
ucaaaaggccuaccgaaccaa
~~~~~~
>gis-dre-mir-209
guaaaagaacaagggaaugccucaguuuuuaauucaagcuaaagagcuuugaaaugaagaguuuuccu
cguucuuccacagg
>5p-matureseq
guaaaagaacaagggaaugccu
>3p-matureseq
guuuuccucguucuuccacagg
~~~~~~

>gis-dre-mir-210
guacaacucaagaugaauuagugcagcuauaagugaaacuaguucuucuugauauguac
>3p-matureseq
cuaguucuucuugauauguac
>5p-matureseq
guacaacucaagaugaauuagu
~~~~~~
>gis-dre-mir-211
guagaagaucagaugguuuuauuauuuguuaaguauuuaauuuccuaauaaaacaucuuuucugucua
gc
>3p-matureseq
auaaaacaucuuuucugucuagc
>5p-matureseq
guagaagaucagaugguuuuauu
~~~~~~
>gis-dre-mir-212
guagcucagacaacacguguggaaaacagguacuguuacuguagucaacacguguagucugagcuacu
g
>3p-matureseq
acacguguagucugagcuacug
>5p-matureseq
guagcucagacaacacgugug
~~~~~~
>gis-dre-mir-213
guaggcugucugcgcaucaaccugaauauucucauuaggcugauuuauaggcgucugccagu
>3p-matureseq
cugauuuauaggcgucugccagu
>5p-matureseq
guaggcugucugcgcaucaaccu
~~~~~~
>gis-dre-mir-214
guaguguccgcugccuggcuuccuuuuucgagaagccgggggaugacauuaccc
>3p-matureseq
agccgggggaugacauuaccc
>5p-matureseq
guaguguccgcugccuggcuuc
~~~~~~
>gis-dre-mir-215
guaucccaucuggacuucaccggccccugcagcauagugaagucuagaugggauacagc
>5p-matureseq
guaucccaucuggacuucacc
>3p-matureseq
ugaagucuagaugggauacagc
~~~~~~
>gis-dre-mir-216
gucacuacaaguuauguugcacaacauuuggggauugguaugacauaacauguagcgaacga
>5p-matureseq
gucacuacaaguuauguugcaca
>3p-matureseq
ugacauaacauguagcgaacga
~~~~~~
>gis-dre-mir-217
gucacugguucgagcucgguccguuggcauuucuguggcauuuuacugguucgagcucgguccauugg
cau
~~~~~~

```
>5p-matureseq
gucacugguucgagcucggucc
>3p-matureseq
uucgagcucgguccauuggcau
~~~~~~~~~~~~~~~~~~~~~~~~~~~~~~~~~~~~~~~~~~~~~~~~~~~
>gis-dre-mir-218
gucaucucugagcgggugugugucgcuucucucccgcagacccgcagauggugaugg
>3p-matureseq
cagacccgcagauggugaugg
>3p-matureseq
gcagacccgcagauggugaugg
>5p-matureseq
gucaucucugagcgggugugug
>5p-matureseq
gucaucucugagcgggugugugu
~~~~~~~~~~~~~~~~~~~~~~~~~~~~~~~~~~~~~~~~~~~~~~~~~~~
>gis-dre-mir-219
gucaugaugagcugauuacucaguugaacucucuuugaggaucaucaucaucaucagccugaucguug
gcgu
>3p-matureseq
caucaucagccugaucguuggcgu
>5p-matureseq
gucaugaugagcugauuacu
>5p-matureseq
gucaugaugagcugauuacuca
>3p-matureseq
ucaucagccugaucguuggcgu
~~~~~~~~~~~~~~~~~~~~~~~~~~~~~~~~~~~~~~~~~~~~~~~~~~~
>gis-dre-mir-220
gucgguggcgccggguuggcggcccugguggugccggugugccaccgcccgaagucgccugcggagg
>3p-matureseq
caccgcccgaagucgccugcggagg
>5p-matureseq
gucgguggcgccggguuggcggcc
~~~~~~~~~~~~~~~~~~~~~~~~~~~~~~~~~~~~~~~~~~~~~~~~~~~
>gis-dre-mir-221
gucuccguguaucuguguuugcacugggugaagagcaguaagcugagugcaaacuaaacacagagaca
cggauacug
>3p-matureseq
aacacagagacacggauacug
>5p-matureseq
gucuccguguaucuguguuug
~~~~~~~~~~~~~~~~~~~~~~~~~~~~~~~~~~~~~~~~~~~~~~~~~~~
>gis-dre-mir-222
gucuccgugugucuguguuugcacugggugaagagcaguaagcugagugcaaaccaaacacagagaca
cggauacug
>3p-matureseq
aacacagagacacggauacug
>5p-matureseq
gucuccgugugucuguguuug
~~~~~~~~~~~~~~~~~~~~~~~~~~~~~~~~~~~~~~~~~~~~~~~~~~~
>gis-dre-mir-223
gucuggaccccagugacuuucaguggaauuacuugaaagucaaugguguccgaacua
>3p-matureseq
```

aaagucaaugguguccgaacua
>5p-matureseq
gucuggaccccagugacuuuca
>5p-matureseq
gucuggaccccagugacuuucag
~~~~~~
>gis-dre-mir-224
gucuugcgcugugcacuugaccuuuugaccucuaaggucgugaccucgcugcaugacugc
>5p-matureseq
gucuugcgcugugcacuugaccu
>3p-matureseq
ucgugaccucgcugcaugacugc
~~~~~~
>gis-dre-mir-225
gugaguguccugagugggaaacgggacagagaaacuguucugcuaacguccugcucagaacaaacaug
c
>3p-matureseq
aacguccugcucagaacaaacaugc
>5p-matureseq
gugaguguccugagugggaaacggg
~~~~~~
>gis-dre-mir-226
gugagugucuccuuggcauuuaucgcuugaguuuguaaauugaccuucguuguuuauuaaugccaugu
ugcauucacagg
>3p-matureseq
aaugccauguugcauucacagg
>5p-matureseq
gugagugucuccuuggcauuua
~~~~~~
>gis-dre-mir-227
gugccuaggaacuguauagaaugccuaaaaugauugcuauuuuauacauugccuaggcguucu
>5p-matureseq
gugccuaggaacuguauagaau
>3p-matureseq
uuauacauugccuaggcguucu
~~~~~~
>gis-dre-mir-228
guggauguuaacgguucuucaugagaauuaucaaagguuccaugaagaacuuuuaacauccaac
>5p-matureseq
guggauguuaacgguucuucaug
>3p-matureseq
ugaagaacuuuuaacauccaac
~~~~~~
>gis-dre-mir-229
guguaacguuucaugcacaccggccuuccucagacugugcucgaaauguugcaug
>3p-matureseq
gacugugcucgaaauguugcaug
>5p-matureseq
guguaacguuucaugcacaccggcc
~~~~~~
>gis-dre-mir-230
guugagacuuuccacccugacuaauuagugcuuuuuaaucgaugcuggauuucucaaccu
>3p-matureseq
aucgaugcuggauuucucaaccu
~~~~~~

>5p-matureseq
guugagacuuuccacccugacu
>3p-matureseq
ucgaugcuggauuucucaaccu
~~~~~~
>gis-dre-mir-231
guuggacaaaagcagcuguaguaggagcaaaaauacuauggcagauuuuucccagcau
>5p-matureseq
guuggacaaaagcagcuguagu
>3p-matureseq
uauggcagauuuuucccagcau
~~~~~~
>gis-dre-mir-232
uaaaguaacuugagaaccccagugggaaacacccacacacuuucucaguuuagcauuauc
>3p-matureseq
acacacuuucucaguuuagcauuauc
>5p-matureseq
uaaaguaacuugagaaccccagugg
~~~~~~
>gis-dre-mir-233
uaaccauugaccuccauaguauuuguuuaugcuacuauugagaucaaugguuaag
>5p-matureseq
uaaccauugaccuccauagu
>3p-matureseq
uauugagaucaaugguuaag
~~~~~~
>gis-dre-mir-234
uaaccuggccggaaaugcuaagccaauaagccccaagcgucuggcuagacucugggccgggagaga
>3p-matureseq
cuagacucugggccgggagaga
>5p-matureseq
uaaccuggccggaaaugcuaagcc
~~~~~~
>gis-dre-mir-235
uaagcacagcuaauuuagaguuugcaccucaguacucuagauuuaguuguucuugga
>5p-matureseq
uaagcacagcuaauuuagagu
>3p-matureseq
ucuagauuuaguuguucuugga
~~~~~~
>gis-dre-mir-236
uaaugcuaagaaagucuuuguaagucccuaagugcugugcaggcaaggugaugguacgggaacaugca
ugugc
>5p-matureseq
uaaugcuaagaaagucuuuguaaguc
>3p-matureseq
ugaugguacgggaacaugcaugugc
~~~~~~
>gis-dre-mir-237
uaaugggcugacaaaacaguauagagauuuagcgcucacuggcugugcugaauuuuuaacuugggaug
aucuggcucgcugga
>3p-matureseq
cuugggaugaucuggcucgcugga
>5p-matureseq
~~~~~~

```
uaaugggcugacaaaacagua
~~~~~~~~~~~~~~~~~~~~~~~~~~~~~~~~~~~~~~~~~~~~~~~~~~~
>gis-dre-mir-238
uaauuuccacauugggucauuucugaacaugacuguuuacauggacaucaguaauggaauuauuugg
>5p-matureseq
uaauuuccacauugggucauuucug
>3p-matureseq
uacauggacaucaguaauggaauuauuugg
~~~~~~~~~~~~~~~~~~~~~~~~~~~~~~~~~~~~~~~~~~~~~~~~~~~
>gis-dre-mir-239
uacacugguuuagauuuucugagguaaauuagaccagauuauaccagacacauua
>5p-matureseq
uacacugguuuagauuuucugag
>3p-matureseq
uuagaccagauuauaccagacacauua
~~~~~~~~~~~~~~~~~~~~~~~~~~~~~~~~~~~~~~~~~~~~~~~~~~~
>gis-dre-mir-240
uacaugaacagucuugacagagccacaggagucaccugcugaaaggagggaugcagacugcucaacgu
caaaaggaggauuauggcu
>3p-matureseq
caacgucaaaaggaggauuauggcu
>5p-matureseq
uacaugaacagucuugacagagccaca
~~~~~~~~~~~~~~~~~~~~~~~~~~~~~~~~~~~~~~~~~~~~~~~~~~~
>gis-dre-mir-241
uacauucauugaugucguugguuucacaugugagcgacucacugaccaaugagugcaaa
>5p-matureseq
uacauucauugaugucguugggu
>3p-matureseq
ucacugaccaaugagugcaaa
~~~~~~~~~~~~~~~~~~~~~~~~~~~~~~~~~~~~~~~~~~~~~~~~~~~
>gis-dre-mir-242
uacgguuugguaagcuuuuauggcuguuuccacugucaaaaggccuaccgaaccaaac
>3p-matureseq
caaaaggccuaccgaaccaaac
>5p-matureseq
uacgguuugguaagcuuuuaugg
>3p-matureseq
ucaaaaggccuaccgaaccaa
~~~~~~~~~~~~~~~~~~~~~~~~~~~~~~~~~~~~~~~~~~~~~~~~~~~
>gis-dre-mir-243
uacucaggaucgcuggucagcagugccucaaaggccuuuaccacuuuuugucuggcuugagcaucccu
gcucagucuccuguuccgu
>5p-matureseq
uacucaggaucgcuggucagcagug
>3p-matureseq
ucccugcucagucuccuguuccgu
~~~~~~~~~~~~~~~~~~~~~~~~~~~~~~~~~~~~~~~~~~~~~~~~~~~
>gis-dre-mir-244
uacuccaagucuguaaccuuugcaaauacaucagaggucccgauuguaaguuauu
>5p-matureseq
uacuccaagucuguaaccuuugcaaauaca
>3p-matureseq
ucagaggucccgauuguaaguuauu
```

~~~~~~
>gis-dre-mir-245
uagaguguaucaugucuacguauguaaugaauauguguagacaugauacacucuaua
>5p-matureseq
uagaguguaucaugucuacgua
>3p-matureseq
uguagacaugauacacucuaua
~~~~~~
>gis-dre-mir-246
uaggaaauaaugcacaccugaguugguuaaguugcucaugugugcauuauucuccaau
>5p-matureseq
uaggaaauaaugcacaccugagu
>3p-matureseq
ucaugugugcauuauucuccaau
~~~~~~
>gis-dre-mir-247
uaggaggcacuagagggcaguagagagcgcgugucgucaucugcgcucucugguacuuccuccugga
>3p-matureseq
gcgcucucugguacuuccuccugga
>5p-matureseq
uaggaggcacuagagggcaguag
~~~~~~
>gis-dre-mir-248
uagguuugguaugcuuuuauggccguuuccacugucaaaaguguaccgaaccaaac
>3p-matureseq
caaaaguguaccgaaccaaac
>5p-matureseq
uagguuugguaugcuuuuauggc
>5p-matureseq
uagguuugguaugcuuuuauggcc
>3p-matureseq
ucaaaaguguaccgaaccaaac
~~~~~~
>gis-dre-mir-249
uaguauuucagcuccaggacugcccacuuuauugccagggccucuuucucaauggcugcguacuugga
uuccgcuguggugagcuuc
>3p-matureseq
cguacuuggauuccgcuguggugagcuuc
>5p-matureseq
uaguauuucagcuccaggacugccc
~~~~~~
>gis-dre-mir-250
uagucacauaugagugaguucuuuggucacauuggauuuauauaacuccuugaauguugacugga
>3p-matureseq
acuccuugaauguugacugga
>5p-matureseq
uagucacauaugagugaguuc
~~~~~~
>gis-dre-mir-251
uagucuuguugguaccugguggugauuguuuacccgugguauucgucguagcuagc
>3p-matureseq
acccgugguauucgucguagcuagc
>5p-matureseq
uagucuuguugguaccugguggugauuguu
~~~~~~

~~~~~~
>gis-dre-mir-252
uauaucagcuuuggugggaggcuugcguuagugucccaccagugcugauauaca
>5p-matureseq
uauaucagcuuuggugggaggcu
>3p-matureseq
ugucccaccagugcugauauaca
~~~~~~
>gis-dre-mir-253
uaucuugauaaaauauauccugcaaguugaccuuuaaaauggucuuuaacuguacucuguuuguaaga
auauu
>5p-matureseq
uaucuugauaaaauauauccugcaagu
>3p-matureseq
uguacucuguuuguaagaauauu
~~~~~~
>gis-dre-mir-254
uaugaagucugcacguguauccauauaugggucacggguccacguguaagcuucauug
>3p-matureseq
guccacguguaagcuucauug
>5p-matureseq
uaugaagucugcacguguauc
~~~~~~
>gis-dre-mir-255
uauugacgcauuccuuucguaggugacaucuaauguauaaaucuaugauagaggaugacguuuaacca
uu
>3p-matureseq
aucuaugauagaggaugacguuuaaccauu
>5p-matureseq
uauugacgcauuccuuucguaggug
~~~~~~
>gis-dre-mir-256
ucaagugucugucagcacccaggcaggaagcaagacccuggugcgucagaacacagcgg
>5p-matureseq
ucaagugucugucagcacccag
>5p-matureseq
ucaagugucugucagcacccagg
>3p-matureseq
uggugcgucagaacacagcgg
~~~~~~
>gis-dre-mir-257
ucaauaacauuuugggcucaugaagguauuuaaauaagcgcaaaauguuguugauu
>3p-matureseq
uaagcgcaaaauguuguugauu
>5p-matureseq
ucaauaacauuuugggcucaug
~~~~~~
>gis-dre-mir-258
ucacaauguagaucuauagauauguacagagcaacaaguagaauagccagugugagguc
>3p-matureseq
acaaguagaauagccagugugagguc
>5p-matureseq
ucacaauguagaucuauagauaugu
~~~~~~

>gis-dre-mir-259
ucaccagucgcucuucauuucuauauuuuuuguccauacaaugaaagcgaauggugacu
>3p-matureseq
acaaugaaagcgaauggugacu
>5p-matureseq
ucaccagucgcucuucauuucua
~~~~~~
>gis-dre-mir-260
ucacucauuuacugcuauaguaagaaaaguaaauacuaugggaguaaauaggugaga
>3p-matureseq
cuaugggaguaaauaggugaga
>5p-matureseq
ucacucauuuacugcuauagu
~~~~~~
>gis-dre-mir-261
ucagcugaucugucagugaauggcuuguauaucuacucauagacagauccucugaug
>3p-matureseq
cucauagacagauccucugaug
>5p-matureseq
ucagcugaucugucagugaaug
~~~~~~
>gis-dre-mir-262
ucaggaaucauggggguuggaccgcgguaugguccuuguucagguggaggugauggag
>5p-matureseq
ucaggaaucauggggguuggaccg
>3p-matureseq
uccuuguucagguggaggugauggag
~~~~~~
>gis-dre-mir-263
ucaggagucaggauuagacgggcugcuugauuuugauccgguuugaucccugauguccuucac
>3p-matureseq
gguuugaucccugauguccuucac
>5p-matureseq
ucaggagucaggauuagacggg
~~~~~~
>gis-dre-mir-264
ucaggcacugucagguucggaccuuuacuguaucgcccagccuucucucugccugccu
>3p-matureseq
ccagccuucucucugccugccu
>5p-matureseq
ucaggcacugucagguucggac
~~~~~~
>gis-dre-mir-265
ucagugaauucucauuggcucuuuuccggccuagcaauaagcagccaaugagauuuuaucugag
>3p-matureseq
cagccaaugagauuuuaucugag
>5p-matureseq
ucagugaauucucauuggcucu
~~~~~~
>gis-dre-mir-266
ucagugcagugguucagcugaaaucaagcauuacauuacagcugaaccacugcacugugu
>3p-matureseq
cagcugaaccacugcacugugu
>5p-matureseq
~~~~~~

ucagugcagugguucagcuga
~~~~~~
>gis-dre-mir-267
ucaguuugguacgauuuuaaggccguuuccacugucaauaagcguacugaaccaaa
>3p-matureseq
ucaauaagcguacugaaccaaa
>5p-matureseq
ucaguuugguacgauuuuaaggc
~~~~~~
>gis-dre-mir-268
ucaguuugguacgcuuuuaugaccguuuccaacuaucaaaaggcguaccaaaccaa
>3p-matureseq
ucaaaaggcguaccaaaccaa
>5p-matureseq
ucaguuugguacgcuuuuaugac
~~~~~~
>gis-dre-mir-269
ucauggcccugaugucuucggaaugaagcuuggugacuuuggucaucuugaaaugucuuggccguccg
ug
>5p-matureseq
ucauggcccugaugucuucggaaugaa
>3p-matureseq
ucuugaaaugucuuggccguccgug
~~~~~~
>gis-dre-mir-270
ucaugucagacgagggccguaauaucugcuagaaacucugacccagaucugacugacu
>5p-matureseq
ucaugucagacgagggccguaau
>3p-matureseq
ucugacccagaucugacugacu
~~~~~~
>gis-dre-mir-271
ucauguccgaggauaaucucuuugcagaggaugacgcccuccuugccaaggcuccucugagaucuccu
gccggcaccca
>5p-matureseq
ucauguccgaggauaaucucuuugcagag
>3p-matureseq
ucugagaucuccugccggcaccca
~~~~~~
>gis-dre-mir-272
uccagaacguucuugccuguguggacauauuugaauucaccgguaaagucguucuggaa
>3p-matureseq
caccgguaaagucguucuggaa
>5p-matureseq
uccagaacguucuugccugugugg
~~~~~~
>gis-dre-mir-273
uccaucaucccucugcgucagcacugucucugcucauguguucaugagaggcuguggaggu
>3p-matureseq
guucaugagaggcuguggagg
>3p-matureseq
guucaugagaggcuguggaggu
>5p-matureseq
uccaucaucccucugcgucagca
~~~~~~

```
>5p-matureseq
uccaucauccucugcgucagcac
~~~~~~~~~~~~~~~~~~~~~~~~~~~~~~~~~~~~~~~~~~~~~~~~~~~
>gis-dre-mir-274
uccaucuauuuggucuguucugagcgacucuccagcccaggccagauggaggagc
>3p-matureseq
gcccaggccagauggaggagc
>5p-matureseq
uccaucuauuuggucuguucug
~~~~~~~~~~~~~~~~~~~~~~~~~~~~~~~~~~~~~~~~~~~~~~~~~~~
>gis-dre-mir-275
uccuggugcagcuucaccacugggcucaguggaucuucuggugcagcuccaccucuggg
>5p-matureseq
uccuggugcagcuucaccacugg
>3p-matureseq
ucuggugcagcuccaccucuggg
~~~~~~~~~~~~~~~~~~~~~~~~~~~~~~~~~~~~~~~~~~~~~~~~~~~
>gis-dre-mir-276
ucgaaugccuacuccuaguccacaaacacauguggacugguugggcauacuucc
>3p-matureseq
acugguugggcauacuucc
>5p-matureseq
ucgaaugccuacuccuagucc
~~~~~~~~~~~~~~~~~~~~~~~~~~~~~~~~~~~~~~~~~~~~~~~~~~~
>gis-dre-mir-277
ucggaaugguaugcauuuauguccuuuuccacugucaaagguaccuaaaagcuaaccauucugug
>3p-matureseq
ccuaaaagcuaaccauucugug
>5p-matureseq
ucggaaugguaugcauuuaugu
~~~~~~~~~~~~~~~~~~~~~~~~~~~~~~~~~~~~~~~~~~~~~~~~~~~
>gis-dre-mir-278
ucggcaggcucugcuaucagcacaggucuuuuguauugaagugcugauaguguaucugucgga
>5p-matureseq
ucggcaggcucugcuaucagcac
>5p-matureseq
ucggcaggcucugcuaucagcaca
>3p-matureseq
ugcugauaguguaucugucgga
~~~~~~~~~~~~~~~~~~~~~~~~~~~~~~~~~~~~~~~~~~~~~~~~~~~
>gis-dre-mir-279
ucgguucgguacgcuuuuauggccguuuccacugucaauaagcguacugaaccaaa
>3p-matureseq
ucaauaagcguacugaaccaaa
>5p-matureseq
ucgguucgguacgcuuuuauggc
~~~~~~~~~~~~~~~~~~~~~~~~~~~~~~~~~~~~~~~~~~~~~~~~~~~
>gis-dre-mir-280
ucgucgacacccaucagauauuuagcaugccaaauaucugacgggugucggugacu
>3p-matureseq
uaucugacgggugucggugacu
>5p-matureseq
ucgucgacacccaucagauauu
~~~~~~~~~~~~~~~~~~~~~~~~~~~~~~~~~~~~~~~~~~~~~~~~~~~
```

>gis-dre-mir-281
ucuaggcuggucaagugaccauguugcuuuaaagugauuuauggcacuugucagccuggaaa
>5p-matureseq
ucuaggcuggucaagugaccaug
>3p-matureseq
uggcacuugucagccuggaaa
~~~~~~
>gis-dre-mir-282
ucuccagcagagagaucuccugaggcccgugcacaccaggauacuuucugcugacguuu
>3p-matureseq
aggauacuuucugcugacguuu
>5p-matureseq
ucuccagcagagagaucuccuga
~~~~~~
>gis-dre-mir-283
ucuccauaucugugggcgcacaaugcuuuccugcauagggguuccuguagauacggagacu
>3p-matureseq
gguuccuguagauacggagacu
>5p-matureseq
ucuccauaucugugggcgcaca
~~~~~~
>gis-dre-mir-284
ucugaaagcauagugccauggaccuuucuggagaccuugaauuauguauccggagu
>3p-matureseq
accuugaauuauguauccggagu
>5p-matureseq
ucugaaagcauagugccauggacc
~~~~~~
>gis-dre-mir-285
ucugaaucugauguuauugcauuagcaugccugauuuagccugucugauuuugaugugcuaauagaau
gucugauuuaaca
>3p-matureseq
aauagaaugucugauuuaaca
>5p-matureseq
ucugaaucugauguuauugcau
~~~~~~
>gis-dre-mir-286
ucugaauuuauuagguggaguuugacuucauucagccccgccucugaauucaauagg
>3p-matureseq
cccgccucugaauucaauagg
>5p-matureseq
ucugaauuuauuagguggaguu
~~~~~~
>gis-dre-mir-287
ucugacaacagggugaaugaaguaaaauaguuuuccccugugagaugagauuaauucacucacccugg
ugucagauu
>3p-matureseq
acucacccuggugucagauu
>5p-matureseq
ucugacaacagggugaaugaa
~~~~~~
>gis-dre-mir-288
ucugacaugucuuuugaugccaauguuaaaugucaauuugacaucauuuuggcaucaaggaaguuuac
au
~~~~~~

>3p-matureseq
gcaucaaggaaguuuacau
>5p-matureseq
ucugacaugucuuuugaugcca
~~~~~~
>gis-dre-mir-289
ucugaccguugacuucuauaguugcaaguuugauacuacggaggucagugguuagag
>3p-matureseq
uacggaggucagugguuagag
>5p-matureseq
ucugaccguugacuucuauagu
~~~~~~
>gis-dre-mir-290
ucugaggcgucaucguguuucuggaucuccaugcagaaacccaggaucccccagagc
>3p-matureseq
aaacccaggaucccccagagc
>5p-matureseq
ucugaggcgucaucguguuucu
~~~~~~
>gis-dre-mir-291
ucugauaguauugugcucugggccuguaaaaacaaagagcuuaaagcaagcuucaacagaaaaccagg
uauaauaauuauugcga
>3p-matureseq
aaaccagguauaauaauuauugcga
>5p-matureseq
ucugauaguauugugcucugggccu
~~~~~~
>gis-dre-mir-292
ucugcccuagggugucccuggugucaugugcacugauggauacccuuaugcucgaaca
>5p-matureseq
ucugcccuaggguguccuggug
>3p-matureseq
ugauggauacccuuaugcucgaaca
~~~~~~
>gis-dre-mir-293
ucugcuuaacgaaccauagacgccagugguuucucggguucguuaagcaauga
>3p-matureseq
ucucggguucguuaagcaauga
>5p-matureseq
ucugcuuaacgaaccauagacg
~~~~~~
>gis-dre-mir-294
ucuggcaccaacugacuuccguaguagaaauacuauggaggucaauuggugccagaaa
>3p-matureseq
ggaggucaauuggugccagaaa
>5p-matureseq
ucuggcaccaacugacuuccgu
~~~~~~
>gis-dre-mir-295
ucuguacaagauuccauuaagcacugaaaguuugucccacugcuuaaggauccguaacacauacaggu
gu
>3p-matureseq
cugcuuaaggauccguaacacauacaggugu
>5p-matureseq
~~~~~~

ucuguacaagauuccauuaagcacu
~~~~~~
>gis-dre-mir-296
ucugugauuuuggugaugugaacugaauuuacuuucacuucaccagcauuacaauaa
>3p-matureseq
cacuucaccagcauuacaauaa
>5p-matureseq
ucugugauuuuggugaugugaa
~~~~~~
>gis-dre-mir-297
ucuuacuguugcucaauggaguuggacaaugaagaaccgacaagaacugaacggcuugugagggc
>3p-matureseq
caagaacugaacggcuugugagggc
>5p-matureseq
ucuuacuguugcucaauggaguu
~~~~~~
>gis-dre-mir-298
ucuugaacgccggaaaacagaggaaugcccguuucucaucuguucuccggcgcucgagcau
>3p-matureseq
ucuguucuccggcgcucgagcau
>5p-matureseq
ucuugaacgccggaaaacaga
>5p-matureseq
ucuugaacgccggaaaacagag
~~~~~~
>gis-dre-mir-299
ugaaccagcgggccuaucuuccugaugcaucuggaauaaugggucaugaagugg
>3p-matureseq
ucuggaauaaugggucaugaagugg
>5p-matureseq
ugaaccagcgggccuaucuucc
~~~~~~
>gis-dre-mir-300
ugaacgugguacagaggagucuaacgcggguccagagaugagugaggaugacuguaaccguucugg
>3p-matureseq
gugaggaugacuguaaccguucugg
>5p-matureseq
ugaacgugguacagaggagucuaacgc
~~~~~~
>gis-dre-mir-301
ugacacccaucaacuucuauagaaggggaaaaauuacuauggaagucaaugggcucagu
>3p-matureseq
auggaagucaaugggcucagu
>5p-matureseq
ugacacccaucaacuucuauag
~~~~~~
>gis-dre-mir-302
ugacucaacucugauuggucagauggcucuacuucauucugauuggucaguugagucugcuc
>5p-matureseq
ugacucaacucugauuggucaga
>3p-matureseq
ugauuggucaguugagucugcuc
~~~~~~
>gis-dre-mir-303

ugacuugaaagggacuugcuagaccaagcuacgacuugacgugacuugaaauaagcagugacuucgcu
ugagucug
>3p-matureseq
auaagcagugacuucgcuugagucug
>5p-matureseq
ugacuugaaagggacuugcuagacc
~~~~~~
>gis-dre-mir-304
ugagagaaaagucuauggcuuggguggcuggagucgcugaugauucucuuggc
>3p-matureseq
agucgcugaugauucucuuggc
>5p-matureseq
ugagagaaaagucuauggcuug
~~~~~~
>gis-dre-mir-305
ugagagaugcgacgacguguugggagcccuauggaggaaucuauccacccguccaucuucugg
>3p-matureseq
ucuauccacccguccaucuucugg
>5p-matureseq
ugagagaugcgacgacguguugggagc
~~~~~~
>gis-dre-mir-306
ugaggcggucauagcaugagguaaaacgagcagagcuuguucgcucagccguguccgccucaccu
>3p-matureseq
ucagccguguccgccucaccu
>5p-matureseq
ugaggcggucauagcaugaggu
~~~~~~
>gis-dre-mir-307
ugaguagcaaugcgauguggcuguagcgucccaccagugaugucacaucgcacugcuucucgug
>3p-matureseq
ucacaucgcacugcuucucgug
>5p-matureseq
ugaguagcaaugcgauguggcu
>5p-matureseq
ugaguagcaaugcgauguggcug
~~~~~~
>gis-dre-mir-308
ugaguccuguuuuagcagaguuuauuuaucauuugagcuuugcugugacuggaucuga
>3p-matureseq
gcuuugcugugacuggaucuga
>5p-matureseq
ugaguccuguuuuagcagaguuu
~~~~~~
>gis-dre-mir-309
ugagucuuguacagggaauaguguucugauccaagggacuggauuggaaauauucacuguuccucugc
auaagauuc
>3p-matureseq
cuguuccucugcauaagauuc
>5p-matureseq
ugagucuuguacagggaauagu
~~~~~~
>gis-dre-mir-310
ugaugacacccauugacuuccaucguuuuucuuucuguagacaucgaugguagcaucca
~~~~~~

```
>3p-matureseq
agacaucgaugggguagcaucca
>5p-matureseq
ugaugacacccauugacuuccaucguuu
~~~~~~~~~~~~~~~~~~~~~~~~~~~~~~~~~~~~~~~~~~~~~~~~~~~
>gis-dre-mir-311
ugauuggucaaauaggucuacauugauugaucaaaugacuacuuugcuaugauuggucuggcgacucu
gcuaggauugaucaga
>3p-matureseq
cgacucugcuaggauugaucaga
>3p-matureseq
gcgacucugcuaggauugaucaga
>5p-matureseq
ugauuggucaaauaggucuac
>5p-matureseq
ugauuggucaaauaggucuaca
~~~~~~~~~~~~~~~~~~~~~~~~~~~~~~~~~~~~~~~~~~~~~~~~~~~
>gis-dre-mir-312
ugauuggucaaauaggucuacauugauugaucaaaugacuacuuugcuaugauuggucuggcgacucu
gcuaugauuggucaga
>3p-matureseq
cgacucugcuaugauuggucaga
>3p-matureseq
gcgacucugcuaugauuggucaga
>5p-matureseq
ugauuggucaaauaggucuac
>5p-matureseq
ugauuggucaaauaggucuaca
~~~~~~~~~~~~~~~~~~~~~~~~~~~~~~~~~~~~~~~~~~~~~~~~~~~
>gis-dre-mir-313
ugcaaguccaaggagaguuuggccccucaguagcuugcugguucaggguaaucuuauguggggggca
ag
>3p-matureseq
guaaucuuauguggggggcaag
>5p-matureseq
ugcaaguccaaggagaguuuggccc
~~~~~~~~~~~~~~~~~~~~~~~~~~~~~~~~~~~~~~~~~~~~~~~~~~~
>gis-dre-mir-314
ugcacuuugaaccgcucuauggcacaccucuuccaaccgggccaggaccggauaaagugagcc
>3p-matureseq
caggaccggauaaagugagcc
>5p-matureseq
ugcacuuugaaccgcucuauggc
~~~~~~~~~~~~~~~~~~~~~~~~~~~~~~~~~~~~~~~~~~~~~~~~~~~
>gis-dre-mir-315
ugcagcccuugaguuucauaguugaguuccgaaaaauacuauggaagucagggguuaccauu
>3p-matureseq
uauggaagucagggguuaccauu
>5p-matureseq
ugcagcccuugaguuucauagu
~~~~~~~~~~~~~~~~~~~~~~~~~~~~~~~~~~~~~~~~~~~~~~~~~~~
>gis-dre-mir-316
ugcauccgcgcagaaacacugaaggucaugauagcguguaucugcugcagaugaggau
>5p-matureseq
```

ugcauccgcgcagaaacacug
>3p-matureseq
uguaucugcugcagaugaggau
~~~~~~
>gis-dre-mir-317
ugcauugcucuacuugaaguuuuuguacugucagaaacuucaagcaaagcagcgcagg
>3p-matureseq
acuucaagcaaagcagcgcagg
>5p-matureseq
ugcauugcucuacuugaaguuu
~~~~~~
>gis-dre-mir-318
ugcgcagccuguggugugguaguaugcaguaauaugcgcacuacacaccacggacuuugcacu
>3p-matureseq
uacacaccacggacuuugcacu
>5p-matureseq
ugcgcagccuguggugugguagu
~~~~~~
>gis-dre-mir-319
ugcguccugauugugaugacgggauauaaaauuggcccacaauucaucuucacaaugaugacgucgu
>3p-matureseq
aucuucacaaugaugacgucgu
>5p-matureseq
ugcguccugauugugaugacgg
~~~~~~
>gis-dre-mir-320
ugcguuugaaagucgagcuuggcaauaccaaguccuccccuucgggcgcucu
>3p-matureseq
caaguccuccccuucgggcgcucu
>5p-matureseq
ugcguuugaaagucgagcuuggc
~~~~~~
>gis-dre-mir-321
ugcucaggcccguuucucagaucguuugagaagugugagcgcucugaaucaggcucaggcacg
>3p-matureseq
cucugaaucaggcucaggcacg
>3p-matureseq
ugaaucaggcucaggcacg
>5p-matureseq
ugcucaggcccguuucucaga
>5p-matureseq
ugcucaggcccguuucucagaucg
~~~~~~
>gis-dre-mir-322
ugcuguuugguuugguauggcccuuuccacugucaaaaggcauaccgaaccuuaccguacc
>3p-matureseq
ggcauaccgaaccuuaccguacc
>5p-matureseq
ugcuguuugguuugguauggccc
~~~~~~
>gis-dre-mir-323
ugcuugagaacgagggcucgccggcgcugcuugagggcgaaggcucgccggcgcugcuugagggcgaa
ggcucg
>3p-matureseq
~~~~~~

cggcgcugcuugagggcgaaggcucg
>3p-matureseq
ggcgcugcuugagggcgaaggcucg
>5p-matureseq
ugcuugagaacgagggcucgccgg
>5p-matureseq
ugcuugagaacgagggcucgccggc
~~~~~~
>gis-dre-mir-324
uggaucuacauugguuagagcacucuacugugauuggucuuuugacuuugcucugcucugauugguca
aauaggucuaca
>5p-matureseq
ggaucuacauugguuaga
>3p-matureseq
ugauuggucaaauaggucuac
>3p-matureseq
ugauuggucaaauaggucuaca
>5p-matureseq
uggaucuacauugguuaga
~~~~~~
>gis-dre-mir-325
uggcagccauugacuuccauuguaggacaacaaaauaugauggaaaucaauggaucucagu
>3p-matureseq
gauggaaaucaauggaucucagu
>5p-matureseq
uggcagccauugacuuccauugu
~~~~~~
>gis-dre-mir-326
uggcauucugcugaaguucagaguuucuuuauuacaagaaacucugaacuucagcagaaugcca
>3p-matureseq
ucugaacuucagcagaaugcca
>5p-matureseq
uggcauucugcugaaguucaga
~~~~~~
>gis-dre-mir-327
uggcauuuggaggcacgagaccacuguuugugagugaggucucgugcccccaaaagacacu
>3p-matureseq
ucucgugcccccaaaagacacu
>5p-matureseq
uggcauuuggaggcacgagacc
~~~~~~
>gis-dre-mir-328
uggcccagauguuaauaauugauaugugggccagauguaaaauguaguauuuggcccagauguuaauu
auugauaugugggccaga
>3p-matureseq
aauuauugauaugugggccaga
>5p-matureseq
uggcccagauguuaauaauuga
~~~~~~
>gis-dre-mir-329
ugggggcaggggaaguugaaguuggugaugguauacucuccuucaacuucugcccucauc
>3p-matureseq
uccuucaacuucugcccucauc
>5p-matureseq

ugggggcaggggaaguugaaguugg
~~~~~~
>gis-dre-mir-330
uguaaucuuugaccuccguaguguuuauuuuccuacuacagagggcaaugguuacaga
>3p-matureseq
uacagagggcaaugguuacaga
>5p-matureseq
uguaaucuuugaccuccguagu
~~~~~~
>gis-dre-mir-331
uguagcacugugauacuggcuguauaucgauacaguauauaggcauaucgcacugcuaugagu
>3p-matureseq
aggcauaucgcacugcuaugagu
>3p-matureseq
uaggcauaucgcacugcuauga
>5p-matureseq
uguagcacugugauacuggcugu
~~~~~~
>gis-dre-mir-332
uguagcuauugacucccauaguguuuauuuugccuacuauggaaguucguuguuacaggu
>3p-matureseq
auggaaguucguuguuacaggu
>5p-matureseq
uguagcuauugacucccauagu
~~~~~~
>gis-dre-mir-333
uguagcuauugacuuuuauagaauguuguacuacuauagauggcaauagcuaccagu
>3p-matureseq
auagauggcaauagcuaccagu
>5p-matureseq
uguagcuauugacuuuuauaga
~~~~~~
>gis-dre-mir-334
uguaucccaucuagaccucacugugcugcaggagaggguugggucuagauuggauacagc
>5p-matureseq
uguaucccaucuagaccucacu
>3p-matureseq
uugggucuagauuggauacagc
~~~~~~
>gis-dre-mir-335
uguaucccaucuggacuucauugcccuguagaagauggugaagucuagaugggauacagc
>3p-matureseq
ugaagucuagaugggauacagc
>5p-matureseq
uguaucccaucuggacuucauu
~~~~~~
>gis-dre-mir-336
ugucacacucauuucaggucagcuuacaagcuguugaucugaauuaggcguguuugg
>3p-matureseq
ugaucugaauuaggcguguuugg
>5p-matureseq
ugucacacucauuucaggucagc
~~~~~~
>gis-dre-mir-337

ugugaacacaugggaaaacguguguauuuggaacgcuuucuugugaauucacaugu
>3p-matureseq
gcuuucuugugaauucacaugu
>5p-matureseq
ugugaacacaugggaaaacgugu
~~~~~~
>gis-dre-mir-338
ugugaggucuuggguugguacuauauaagagucaaauucuagguuuucguagucuguauauucguagc
uucaguacc
>3p-matureseq
guauauucguagcuucaguacc
>5p-matureseq
ugugaggucuuggguugguacu
~~~~~~
>gis-dre-mir-339
uguggugauuagaacaggcugacuuuugcaagugugaugcggccuacuaacaccacaca
>3p-matureseq
ugcggccuacuaacaccacaca
>5p-matureseq
uguggugauuagaacaggcugacu
~~~~~~
>gis-dre-mir-340
ugugugagugcagcagugcaguagacggcagugcaugaacagcuuucuguguucugcgcuacgcacuc
gccacu
>3p-matureseq
ucugcgcuacgcacucgccacu
>5p-matureseq
ugugugagugcagcagugcaguag
~~~~~~
>gis-dre-mir-341
uguuauacacagacccggauuggccaaucgggaggaccgggagaguucccggugggccagucuguuuu
uuggccgcgag
>3p-matureseq
ccagucuguuuuuuggccgcgag
>5p-matureseq
uguuauacacagacccggauuggcc
~~~~~~
>gis-dre-mir-342
uuacuuggaaggucacaucaccaugaguaguuaagaaugguugaugaugugccucuccgaguaaa
>3p-matureseq
ugaugugccucuccgaguaaa
>5p-matureseq
uuacuuggaaggucacaucac
~~~~~~
>gis-dre-mir-343
uuagacugaaaucuguuggagccaacauuuuccccagccgaacugcggcucuucagcacgacgggagc
uaguu
>3p-matureseq
cuucagcacgacgggagcuaguu
>5p-matureseq
uuagacugaaaucuguuggagcc
~~~~~~
>gis-dre-mir-344
uuagacugaaaucuguuggagccaacauuuuccccagccgagcugcggcucuucagcacgacgggagc
~~~~~~

uaguu
>3p-matureseq
cuucagcacgacgggagcuaguu
>5p-matureseq
uuagacugaaaucuguuggagcc
~~~~~~
>gis-dre-mir-345
uuaggaucggguuuggacacccuugcucuaagccagagguguccaaacucgauccuggag
>3p-matureseq
uguccaaacucgauccuggag
>5p-matureseq
uuaggaucggguuuggacacc
~~~~~~
>gis-dre-mir-346
uuagucacauuugggcaaguucuuugaucaucuugauuaucuggaacuccuuggauguugacugga
>3p-matureseq
aacuccuuggauguugacugga
>5p-matureseq
uuagucacauuugggcaaguucu
~~~~~~
>gis-dre-mir-347
uuagucacauuugggcgaguucuuuggucaucuugaauuuauggaacuccuuggauguugacugga
>3p-matureseq
aacuccuuggauguugacugga
>5p-matureseq
uagucacauuugggcgaguucu
>5p-matureseq
uuagucacauuugggcgaguucu
~~~~~~
>gis-dre-mir-348
uuagucacauuugggcgaguucuuuggucaucuuggauuuauggaacuccuuggauguugacugga
>3p-matureseq
aacuccuuggauguugacugga
>5p-matureseq
uagucacauuugggcgaguucu
>5p-matureseq
uuagucacauuugggcgaguucu
~~~~~~
>gis-dre-mir-349
uucacuguggcggaaaugaccucgguaacauaaagaguguuauacuugccgcagugaug
>3p-matureseq
uuauacuugccgcagugaug
>5p-matureseq
uucacuguggcggaaaugaccuc
~~~~~~
>gis-dre-mir-350
uucacuguggcggaaaugaccucgguaacaucaagaguguuauacuugccgcagugaug
>3p-matureseq
uuauacuugccgcagugaug
>5p-matureseq
uucacuguggcggaaaugaccuc
~~~~~~
>gis-dre-mir-351
uucaguuugguacgcuuuuauggcuguuuccacugucaaaggcauaccaaaccgaacc
~~~~~~

```
>3p-matureseq
aaaaggcauaccaaaccgaacc
>5p-matureseq
uucaguuugguacgcuuuuaugg
~~~~~~~~~~~~~~~~~~~~~~~~~~~~~~~~~~~~~~~~~~~~~~~~~~~
>gis-dre-mir-352
uuccaaauguauuucuauguauuauuuauacaaagaaauacauuuggaca
>3p-matureseq
uacaaagaaauacauuuggaca
>5p-matureseq
uuccaaauguauuucuauguau
~~~~~~~~~~~~~~~~~~~~~~~~~~~~~~~~~~~~~~~~~~~~~~~~~~~
>gis-dre-mir-353
uucgguaaugacacggaagcugggauggcaucgcuggcugucguauuauuagcggaag
>3p-matureseq
ggcugucguauuauuagcggaag
>5p-matureseq
uucgguaaugacacggaagcug
~~~~~~~~~~~~~~~~~~~~~~~~~~~~~~~~~~~~~~~~~~~~~~~~~~~
>gis-dre-mir-354
uucucaguucagcgugucaaauguugauucgaaaugauuuggacucacugaaccgagaau
>5p-matureseq
uucucaguucagcgugucaaa
>3p-matureseq
uuggacucacugaaccgagaau
~~~~~~~~~~~~~~~~~~~~~~~~~~~~~~~~~~~~~~~~~~~~~~~~~~~
>gis-dre-mir-355
uucucuugcguccuuggaaguucagacuugcaagaacgaacucccgaggacgugaggac
>3p-matureseq
acucccgaggacgugaggac
>5p-matureseq
uucucuugcguccuuggaaguuc
~~~~~~~~~~~~~~~~~~~~~~~~~~~~~~~~~~~~~~~~~~~~~~~~~~~
>gis-dre-mir-356
uugacagcaguccuccagucaugcugcucgagcuuaucugcagcgcuggagcugcuuucagcc
>3p-matureseq
gcgcuggagcugcuuucagcc
>5p-matureseq
uugacagcaguccuccagucaug
~~~~~~~~~~~~~~~~~~~~~~~~~~~~~~~~~~~~~~~~~~~~~~~~~~~
>gis-dre-mir-357
uugaccagccuggacauagcuggucuuggcugggcucccagccagaccagcuaagaccaggcuggaau
uggc
>3p-matureseq
agcuaagaccaggcuggaauuggc
>5p-matureseq
uugaccagccuggacauagcugg
~~~~~~~~~~~~~~~~~~~~~~~~~~~~~~~~~~~~~~~~~~~~~~~~~~~
>gis-dre-mir-358
uugaccagccuggacauagcugguuuggcuggacucccagcuuggcuaggcuggucaagc
>3p-matureseq
agcuuggcuaggcuggucaagc
>5p-matureseq
uugaccagccuggacauagcugg
```

~~~~~~
>gis-dre-mir-359
uugaccagccuggacauagcugguuuuggcugggcucccagcuuggguaggcuggucaagc
>3p-matureseq
agcuuggguaggcuggucaagc
>5p-matureseq
uugaccagccuggacauagcugg
~~~~~~
>gis-dre-mir-360
uugagaaucacugcaaucauucagcuuaauauucugguggugacuuuaacuggauguuuguguguuuc
ucagag
>3p-matureseq
uguuuguguguuucucagag
>5p-matureseq
uugagaaucacugcaaucauu
~~~~~~
>gis-dre-mir-361
uugauuugguacacuuuuguggccguuuccacugucaaaggcguaccgaaccaaac
>3p-matureseq
caaaggcguaccgaaccaaac
>5p-matureseq
uugauuugguacacuuuugug
~~~~~~
>gis-dre-mir-362
uugcuuuauaguuuaacacucgcugguuauaaugagcuuguuaaacucuaaagcaca
>3p-matureseq
cuuguuaaacucuaaagcaca
>5p-matureseq
uugcuuuauaguuuaacacucgcu
~~~~~~
>gis-dre-mir-363
uuggagaaaggauuuccugaacaaaggaagauguguuuguugaggauccucucucgca
>3p-matureseq
ugaggauccucucucgca
>5p-matureseq
uuggagaaaggauuuccugaac
~~~~~~
>gis-dre-mir-364
uuggcuaaucgggcagacugggagaauucccggugggccggucugucuuuuggccgcga
>3p-matureseq
cggucugucuuuuggccgcga
>5p-matureseq
uuggcuaaucgggcagacuggg
~~~~~~
>gis-dre-mir-365
uugggggggaaauacacaggugggcugauaauuaugacuucaacucuguguuuuucucuccaaca
>3p-matureseq
acucuguguuuuucucuccaaca
>5p-matureseq
uugggggggaaauacacaggugggg
~~~~~~
>gis-dre-mir-366
uuggguaccguucagguuuuuacugguauuguacuuguaaugcggcacugguauccaucu
>3p-matureseq

aaugcggcacugguauccaucu
>5p-matureseq
uuggguaccguucagguuuuua
~~~~~~
>gis-dre-mir-367
uuguggugauuagaacaggcugacuucugcaagucugaugcggccuacuaacaccacacacaugc
>3p-matureseq
gccuacuaacaccacacacaugc
>3p-matureseq
ugcggccuacuaacaccacaca
>5p-matureseq
uguggugauuagaacaggcugacu
>5p-matureseq
uuguggugauuagaacaggcuga
~~~~~~
>gis-dre-mir-368
uuuagaggagagaugauggagcgcuguguguugaaugcucuguauucucuccucugaggac
>3p-matureseq
ucuguauucucuccucugaggac
>5p-matureseq
uuuagaggagagaugauggagcg
~~~~~~
>gis-dre-mir-369
uuuccaccccucugucccauagcagaugugugugucugggacaggcaggggcagaau
>3p-matureseq
ucugggacaggcaggggcagaau
>5p-matureseq
uuuccaccccucugucccauag
~~~~~~
>gis-dre-mir-370
uuugccaugauaugcagauauguguuuaaaacacuuuuuauaucuguauaucauggcaaaau
>3p-matureseq
uaucuguauaucauggcaaaau
>5p-matureseq
uuugccaugauaugcagauaug
~~~~~~
>gis-dre-mir-371
uuugggagcagagauugaaacagcccauuaaaaaaguuguuuuuuuuuuugccccacaac
>5p-matureseq
uuugggagcagagauugaaac
>3p-matureseq
uuuuuuuuuuugccccacaac
~~~~~~
>gis-dre-mir-372
uuuggggagacagaaaucguguccuuuccaugauuugugauaaauuugugucuccucagc
>3p-matureseq
gauaaauuugugucuccucagc
>5p-matureseq
uuuggggagacagaaaucguguc
~~~~~~
>gis-dre-mir-373
uuugguucgguacgccuuuugacaguggaaacggccauaaaagcguaccaaacagaacc
>3p-matureseq
aaaagcguaccaaacagaacc
~~~~~~

>5p-matureseq
uuugguucgguacgccuuuuga
~~~~~~
>gis-dre-mir-374
uuugguucgguacgccuuuugacaguggaaacggccauaaaagcguaccaaaccgaacc
>3p-matureseq
aaaagcguaccaaaccgaacc
>3p-matureseq
auaaaagcguaccaaaccgaacc
>3p-matureseq
uaaaagcguaccaaaccgaacc
>5p-matureseq
uuugguucgguacgccuuuuga
~~~~~~
>gis-dre-mir-375
uuugguucgguacgcuuauugacaguggaaauggacauaaaagcguaccaaaccgaacc
>3p-matureseq
aaaagcguaccaaaccgaacc
>3p-matureseq
auaaaagcguaccaaaccgaacc
>3p-matureseq
uaaaagcguaccaaaccgaacc
>5p-matureseq
uuugguucgguacgcuuauuga
~~~~~~
>gis-dre-mir-376
uuugguucgguucgcuuuugacaguggaaacggccauaaaagcguaccaaaccgaacc
>3p-matureseq
aaaagcguaccaaaccgaacc
>3p-matureseq
auaaaagcguaccaaaccgaacc
>3p-matureseq
uaaaagcguaccaaaccgaacc
>5p-matureseq
uuugguucgguucgcuuuugac
~~~~~~
>gis-dre-mir-377
uuuucggucagugacuggugccaguaauugagcuggcuuucucucacguccugagaccccgc
>3p-matureseq
gcuuucucucacguccugagaccccgc
>5p-matureseq
uuuucggucagugacuggugcc
~~~~~~
>gis-dre-mir-378
uuuuuacccucugacaccacaauggaaucaauugaacaguugcaaauuucuucaggugucucaagggg
caaaagg
>3p-matureseq
caggugucucaaggggcaaaagg
>5p-matureseq
uuuuuacccucugacaccacaa
~~~~~~
>gis-dre-mir-379
aaagauccuaauuaugccacacaucgucguuuguugcauaauuagggucucgcu
>5p-matureseq

aaagauccuaauuaugccac
>5p−matureseq
aaagauccuaauuaugccaca
>3p−matureseq
ugcauaauuagggucucgcu
>3p−matureseq
uugcauaauuagggucucgcu
~~~~~~
>gis−dre−mir−380
aaaguaacaagugucaagugacuggacaccaucgucacuugacacuuguuacuuuuc
>5p−matureseq
aaaguaacaagugucaagugac
>5p−matureseq
aaguaacaagugucaagugacu
>3p−matureseq
cacuugacacuuguuacuuuuc
>3p−matureseq
ucacuugacacuuguuacuuuu
~~~~~~
>gis−dre−mir−381
aacacggcuagugacuggucaagguuuacuuucuucuccagucaugguuguguggu
>5p−matureseq
aacacggcuagugacugguca
>5p−matureseq
aacacggcuagugacuggucaag
>3p−matureseq
cuccagucaugguuguguggu
>3p−matureseq
uccagucaugguuguguggu
~~~~~~
>gis−dre−mir−382
aacagcccugaaauucagacagcugugcagugacuucaugucugaauacacggacugcucc
>5p−matureseq
aacagcccugaaauucagaca
>5p−matureseq
acagcccugaaauucagaca
>3p−matureseq
ucugaauacacggacugcuc
>3p−matureseq
ucugaauacacggacugcucc
~~~~~~
>gis−dre−mir−383
aauggaaauacucgcugauacugcaagaauuguggcaucgguauaagccaguauuuccgaua
>5p−matureseq
aauggaaauacucgcugauacu
>3p−matureseq
guauaagccaguauuuccgaua
>3p−matureseq
uauaagccaguauuuccgaua
~~~~~~
>gis−dre−mir−384
acacgauacaauguucuagaaugcugggauggccauucuagaacauuuugucgugucu
>5p−matureseq
acacgauacaauguucuagaaug
~~~~~~

>5p-matureseq
acacgauacaauguucuagaaugcug
>5p-matureseq
cacgauacaauguucuagaaug
>3p-matureseq
uucuagaacauuuugucguguc
>3p-matureseq
uucuagaacauuuugucgugucu
~~~~~~
>gis-dre-mir-385
acaggaauuuggccacugcacucuuucagauaaguuuggugcuguggccaaauuccuguaa
>5p-matureseq
acaggaauuuggccacugca
>5p-matureseq
acaggaauuuggccacugcacu
>3p-matureseq
ugcuguggccaaauuccuguaa
~~~~~~
>gis-dre-mir-386
acugaguuuggacaccauuguucuauaaauuaugauauuguuguccaaacucggucc
>5p-matureseq
acugaguuuggacaccauuguu
>5p-matureseq
cugaguuuggacaccauuguu
>3p-matureseq
uauuguuguccaaacucgguc
>3p-matureseq
uauuguuguccaaacucggucc
~~~~~~
>gis-dre-mir-387
agacaacuugcuggagagcuuuuuuuucugugauuuuucaaugaacucuaaaaagcucuccagcgaau
ugcccua
>5p-matureseq
agacaacuugcuggagagcuu
>5p-matureseq
agacaacuugcuggagagcuuu
>3p-matureseq
agcucuccagcgaauugcccua
~~~~~~
>gis-dre-mir-388
agagagaaaagucuguggcuuggguggcuggagucacugauaauucucuuggc
>5p-matureseq
agagagaaaagucuguggcuug
>3p-matureseq
agucacugauaauucucuuggc
~~~~~~
>gis-dre-mir-389
agagagaaaugucuauggcuuggguggcuggagucauugauaauucucuuggc
>5p-matureseq
agagagaaaugucuauggcuug
>3p-matureseq
agucauugauaauucucuuggc
>3p-matureseq
gucauugauaauucucuuggc
~~~~~~

~~~~~~
>gis-dre-mir-390
agccauugacuuucuuaguaugaaaaacaacuacuaagcaagucaguggucac
>5p-matureseq
agccauugacuuucuuaguaug
>3p-matureseq
uacuaagcaagucaguggucac
~~~~~~
>gis-dre-mir-391
agcugguuaacccacaccagcagaacccaccgcuucaugcuagugugggucaaccggcgcu
>5p-matureseq
agcugguuaacccacaccagca
>3p-matureseq
cuagugugggucaaccggcgc
>3p-matureseq
cuagugugggucaaccggcgcu
>5p-matureseq
gcugguuaacccacaccagca
~~~~~~
>gis-dre-mir-392
aggagguggagucgggacacggccaggcuuguuaggaaugucaucccgaaagcaccucccucc
>5p-matureseq
aggagguggagucgggacacggcc
>5p-matureseq
ggagguggagucgggacacggcc
>5p-matureseq
ggagguggagucgggacacggcca
>3p-matureseq
ucaucccgaaagcaccucccuc
>3p-matureseq
ucaucccgaaagcaccucccucc
~~~~~~
>gis-dre-mir-393
agggccaugacggaaaucagccuguguucuuuaagguucucucugguccugucuuggcuuac
>5p-matureseq
agggccaugacggaaaucagcc
>5p-matureseq
agggccaugacggaaaucagccu
>3p-matureseq
ucugguccugucuuggcuuac
~~~~~~
>gis-dre-mir-394
aguaccugcgaaugcuuggccaugaguguuucuugcugcacuaggacuuacucaugucgcuggacugu
agaagccu
>5p-matureseq
aguaccugcgaaugcuuggccaugagugu
>5p-matureseq
guaccugcgaaugcuuggccaugaguguuu
>3p-matureseq
ucaugucgcuggacuguagaagccu
~~~~~~
>gis-dre-mir-395
aguacgguucacuuuuagauaccuuuugacaguggaaacggcccuaaaagcguaccguaacg
>5p-matureseq

aguacgguucacuuuuagauac
>3p-matureseq
gcccuaaaagcguaccguaacg
>5p-matureseq
guacgguucacuuuuagauacc
~~~~~~
>gis-dre-mir-396
agugcaaaguuuaacaugcgcgcugugugugucagcugugugcgcaugcgugaacuuuguacc
>5p-matureseq
agugcaaaguuuaacaugcgc
>5p-matureseq
agugcaaaguuuaacaugcgcg
>5p-matureseq
agugcaaaguuuaacaugcgcgc
>5p-matureseq
ugcaaaguuuaacaugcgcgc
>5p-matureseq
ugcaaaguuuaacaugcgcgcu
>5p-matureseq
ugcaaaguuuaacaugcgcgcug
>3p-matureseq
ugcgcaugcgugaacuuuguacc
~~~~~~
>gis-dre-mir-397
aguucacuuggauuggcugaucuauuuugcagugaucggccgacccagugugcugug
>5p-matureseq
aguucacuuggauuggcugauc
>3p-matureseq
ucggccgacccagugugcugu
>3p-matureseq
ucggccgacccagugugcugug
~~~~~~
>gis-dre-mir-398
auaaccaucgauguccauagguuuuauuuuuccaucuaugcaugucaaugguuaucagu
>5p-matureseq
auaaccaucgauguccauagg
>5p-matureseq
auaaccaucgauguccauaggu
>3p-matureseq
augcaugucaaugguuaucagu
>3p-matureseq
uaugcaugucaaugguuaucagu
~~~~~~
>gis-dre-mir-399
auaucagcacuggugggaggugugcguugcucuuagucuccuaccagugcugauauac
>5p-matureseq
auaucagcacuggugggaggugu
>5p-matureseq
uaucagcacuggugggaggugug
>3p-matureseq
ucuccuaccagugcugauauac
~~~~~~
>gis-dre-mir-400
auguauaucgacacuggugaguggagugccuuauugucccaccagugaugauauacagc
~~~~~~

>5p-matureseq
auguauaucgacacuggugagu
>3p-matureseq
cccaccagugaugauauacagc
>3p-matureseq
ucccaccagugaugauauacagc
~~~~~~
>gis-dre-mir-401
augucagaugugagaugagcgugaucagauuauaccgcagcacaucuccacucaucugucaug
>3p-matureseq
acaucuccacucaucugucaug
>5p-matureseq
augucagaugugagaugagcgu
>3p-matureseq
cacaucuccacucaucugucaug
>5p-matureseq
ugucagaugugagaugagcg
~~~~~~
>gis-dre-mir-402
auguuucuccagcuugcugauuuugcauuucugcucaaaucuaagugaacugagaaaaacagg
>5p-matureseq
auguuucuccagcuugcugauu
>5p-matureseq
auguuucuccagcuugcugauuu
>3p-matureseq
uaagugaacugagaaaaacagg
~~~~~~
>gis-dre-mir-403
ccaaacuuugugaccccaugacuuuccgcuagacugucaugcggacauaaaguugggc
>5p-matureseq
ccaaacuuugugaccccaugac
>3p-matureseq
ucaugcggacauaaaguugggc
~~~~~~
>gis-dre-mir-404
ccgcggacggugugaggccggugucggcccccgccccgccggggu
>5p-matureseq
ccgcggacggugugaggccgg
>5p-matureseq
ggacggugugaggccggu
>3p-matureseq
ggcccccgccccgccggggu
~~~~~~
>gis-dre-mir-405
ccuagagugucaacugcuguucaaaacuagacuaguuuaacaacaugucacucuaggcu
>3p-matureseq
aacaacaugucacucuaggcu
>5p-matureseq
ccuagagugucaacugcuguuca
~~~~~~
>gis-dre-mir-406
ccuguagccacugacuuucauaguauguguuuuuccuacuauagagaucaauggcuacaagu
>5p-matureseq
ccuguagccacugacuuucau

>5p-matureseq
cuguagccacugacuuucauagu
>3p-matureseq
uauagagaucaauggcuacaagu
~~~~~~
>gis-dre-mir-407
cgaguagcagugcgauguggcugugauuauauacagcuacauugcacuucuacgagu
>5p-matureseq
cgaguagcagugcgauguggcugu
>3p-matureseq
cuacauugcacuucuacgagu
>5p-matureseq
gaguagcagugcgauguggcug
~~~~~~
>gis-dre-mir-408
cggcacuuuggggcgaaggcucgccggcacuuuggggcgaaggcucgccggcgcugcuugagaacgag
ggcucgccggc
>5p-matureseq
cggcacuuuggggcgaaggcucg
>5p-matureseq
ggcacuuuggggcgaaggcucg
>3p-matureseq
ugcuugagaacgagggcucgccgg
>3p-matureseq
ugcuugagaacgagggcucgccggc
~~~~~~
>gis-dre-mir-409
cgguuugguaggcuuuuauggcuguuuccacugucaaaagguguaccggaccgua
>5p-matureseq
cgguuugguaggcuuuuauggcu
>3p-matureseq
ucaaaagguguaccggaccgua
~~~~~~
>gis-dre-mir-410
cguguaauuguuugugguuuauacgguguaggugaccauaugaaccacagacgauuaccgcu
>5p-matureseq
cguguaauuguuugugguuuaua
>3p-matureseq
ugaaccacagacgauuaccgcu
~~~~~~
>gis-dre-mir-411
cuagucacauucaggcgaguucuuugauuauuuugaauucaugguacuccuuggauguugacugga
>3p-matureseq
augguacuccuuggauguugac
>5p-matureseq
cacauucaggcgaguucuuuga
>5p-matureseq
cuagucacauucaggcgagu
>3p-matureseq
uacuccuuggauguugacugga
>5p-matureseq
uagucacauucaggcgaguucu
~~~~~~
>gis-dre-mir-412

cuagucacauuugggcgaguucuuuguucaucuugaauucaugguacuccuuggauguugacugga
>3p-matureseq
augguacuccuuggauguugac
>5p-matureseq
cuagucacauuugggcgaguucu
>3p-matureseq
uacuccuuggauguugacugga
>5p-matureseq
uagucacauuugggcgaguucu
~~~~~~
>gis-dre-mir-413
cuccaaccuugaucaagcagcucaauaauaauuacagacggcugauugaacaggguuggaag
>5p-matureseq
cuccaaccuugaucaagcagcu
>3p-matureseq
cugauugaacaggguuggaag
~~~~~~
>gis-dre-mir-414
cucgcucuccuucgacuuguugugacgcagacauaaaacacagggagauggagagggagg
>3p-matureseq
cacagggagauggagagggagg
>5p-matureseq
cucgcucuccuucgacuuguu
>5p-matureseq
cucgcucuccuucgacuuguugu
~~~~~~
>gis-dre-mir-415
cugguggucggguacccugcaggguuuagcuaaaaucugcaggacaccggaccuccagga
>3p-matureseq
aggacaccggaccuccagga
>5p-matureseq
cugguggucggguacccugc
>3p-matureseq
ugcaggacaccggaccuccagg
>5p-matureseq
ugguggucggguacccugcagg
~~~~~~
>gis-dre-mir-416
cuuacaauuaaaggauauuucuugcggcugaauacgagaacagaaaugucccuuaauuguuuggu
>3p-matureseq
aaaugucccuuaauuguuuggu
>3p-matureseq
agaaaugucccuuaauuguuug
>5p-matureseq
cuuacaauuaaaggauauuucu
>5p-matureseq
uuacaauuaaaggauauuucuu
~~~~~~
>gis-dre-mir-417
gacuuggucuaagcuccucagugugaugauuaaaccugaggaguuuagagcaaguaa
>5p-matureseq
gacuuggucuaagcuccucagu
>3p-matureseq
ugaggaguuuagagcaaguaa
~~~~~~

~~~~~~
>gis-dre-mir-418
gagcaguccguguauucagacaugaagucacugcacagcugucugaauuucagggcuguuccc
>5p-matureseq
gagcaguccguguauucagaca
>3p-matureseq
ucugaauuucagggcuguuccc
~~~~~~
>gis-dre-mir-419
gaggucacgcgagugguggcaggguuaccacaaacuaaccgaaucuuccaucagucacgugaccuac
>5p-matureseq
gaggucacgcgagugguggcag
>3p-matureseq
uccaucagucacgugaccuac
~~~~~~
>gis-dre-mir-420
gauggaaugggacgacaaagccaaaaaaacauacuucuuugcuuuuaucgccuuguuccauucc
>5p-matureseq
gauggaaugggacgacaaagc
>5p-matureseq
gauggaaugggacgacaaagcc
>5p-matureseq
gauggaaugggacgacaaagcca
>3p-matureseq
uuuuaucgccuuguuccauucc
~~~~~~
>gis-dre-mir-421
gauuccuauauacaguuugcuuaaauguuaacauucaagcuguauauaugaacauc
>5p-matureseq
gauuccuauauacaguuugcuu
>3p-matureseq
ucaagcuguauauaugaacauc
~~~~~~
>gis-dre-mir-422
gccgguguucugcugaguuuagcuaggguuggaccuagacucuccaguacacuggcccu
>3p-matureseq
agacucuccaguacacuggcccu
>5p-matureseq
gccgguguucugcugaguuuagcuagg
>5p-matureseq
gguguucugcugaguuuagcu
~~~~~~
>gis-dre-mir-423
gcggcgcuggaguuuccuguugaaugucccugucgagcgcaacaggaacuuccagcccgagc
>3p-matureseq
acaggaacuuccagcccgagc
>3p-matureseq
caacaggaacuuccagcccga
>5p-matureseq
gcggcgcuggaguuuccuguug
>5p-matureseq
ggcgcuggaguuuccuguugaa
~~~~~~
>gis-dre-mir-424
~~~~~~

gcuggacauuugcgcaagcaauuuuaucucuaaaauugcaugcauaaauguccggu
>3p-matureseq
auugcaugcauaaauguccggu
>5p-matureseq
gcuggacauuugcgcaagcaau
>3p-matureseq
uugcaugcauaaauguccggu
~~~~~~
>gis-dre-mir-425
gcuguggcucuucagcagggccaugagaaugugguggcccugcucaucaaccacgguacu
>3p-matureseq
ccugcucaucaaccacgguacu
>5p-matureseq
gcuguggcucuucagcagggc
~~~~~~
>gis-dre-mir-426
ggccaguugccugacauggacgagaugccuacggguuguucguggcacgcuauccuggca
>5p-matureseq
ggccaguugccugacauggac
>5p-matureseq
ggccaguugccugacauggacg
>5p-matureseq
ggccaguugccugacauggacga
>3p-matureseq
guucguggcacgcuauccuggca
~~~~~~
>gis-dre-mir-427
ggcucaaagauggagagagugcaguucauucacucccuccgucauugaauuccu
>3p-matureseq
cucccuccgucauugaauuccu
>5p-matureseq
ggcucaaagauggagagagug
>5p-matureseq
ggcucaaagauggagagagugc
>3p-matureseq
ucccuccgucauugaauuccu
~~~~~~
>gis-dre-mir-428
gguguuguguguuaaacuguauuccuaaacaacaucuauaguuugauucacagcacaag
>5p-matureseq
gguguuguguguuaaacugua
>3p-matureseq
uaguuugauucacagcacaag
~~~~~~
>gis-dre-mir-429
gguuugguucgguaugccuuuugacaguggaaacggcuauaaaggcguaccuaaccgaacugu
>3p-matureseq
aggcguaccuaaccgaacugu
>5p-matureseq
gguuugguucgguaugccuuu
>5p-matureseq
uugguucgguaugccuuuuga
>5p-matureseq
uugguucgguaugccuuuugac
~~~~~~

```
~~~~~~~~~~~~~~~~~~~~~~~~~~~~~~~~~~~~~~~~~~~~~~~~~~~
>gis-dre-mir-430
guagccauugacuucuauaguguuuguuuuuccgacuacaugagucaaugguuaccg
>5p-matureseq
guagccauugacuucuauaguguuug
>3p-matureseq
uacaugagucaaugguuaccg
~~~~~~~~~~~~~~~~~~~~~~~~~~~~~~~~~~~~~~~~~~~~~~~~~~~
>gis-dre-mir-431
guagccugaacaaaaccugauuguauucuaguucuauucaagcuuuguucaagcuaug
>5p-matureseq
guagccugaacaaaaccugauu
>5p-matureseq
uagccugaacaaaaccugauug
>3p-matureseq
uucaagcuuuguucaagcuaug
~~~~~~~~~~~~~~~~~~~~~~~~~~~~~~~~~~~~~~~~~~~~~~~~~~~
>gis-dre-mir-432
guaucccaucuagacuucacuucacugcaggagagggugaggucuagaugggauacagc
>5p-matureseq
guaucccaucuagacuucacu
>3p-matureseq
ugaggucuagaugggauacagc
~~~~~~~~~~~~~~~~~~~~~~~~~~~~~~~~~~~~~~~~~~~~~~~~~~~
>gis-dre-mir-433
guauccuaucuggacuucacagucuccugcagcauggugaacgcuagaugggauacag
>5p-matureseq
guauccuaucuggacuucaca
>3p-matureseq
ugaacgcuagaugggauacag
~~~~~~~~~~~~~~~~~~~~~~~~~~~~~~~~~~~~~~~~~~~~~~~~~~~
>gis-dre-mir-434
gugaggaucaucgugaggucaguguguguuuauaaugagcgucucuuauuaaaagcacugacuuuucu
cuggucugcaca
>5p-matureseq
gugaggaucaucgugaggucagu
>3p-matureseq
ugacuuuucucuggucugcaca
>5p-matureseq
ugaggaucaucgugaggucagu
~~~~~~~~~~~~~~~~~~~~~~~~~~~~~~~~~~~~~~~~~~~~~~~~~~~
>gis-dre-mir-435
guucgguucgguacgcuuuuauggccguuuccacugucaaaaguguaccgaaccaaaccg
>3p-matureseq
caaaaguguaccgaaccaaac
>3p-matureseq
caaaaguguaccgaaccaaacc
>3p-matureseq
caaaaguguaccgaaccaaaccg
>5p-matureseq
guucgguucgguacgcuuuuauggc
>3p-matureseq
ucaaaaguguaccgaaccaaac
>5p-matureseq
```

ucgguucgguacgcuuuuauggc
>5p−matureseq
uucgguucgguacgcuuuuaugg
>5p−matureseq
uucgguucgguacgcuuuuauggc
~~~~~~
>gis−dre−mir−436
guucgguuugguacgcuuuuaugaccauuuccauugucaaaaguguaccgaaccaaaccg
>3p−matureseq
caaaaguguaccgaaccaaac
>3p−matureseq
caaaaguguaccgaaccaaacc
>3p−matureseq
caaaaguguaccgaaccaaaccg
>5p−matureseq
guucgguuugguacgcuuuuaugac
>3p−matureseq
ucaaaaguguaccgaaccaaac
>5p−matureseq
ucgguuugguacgcuuuuauga
>5p−matureseq
uucgguuugguacgcuuuuauga
>5p−matureseq
uucgguuugguacgcuuuuaugac
~~~~~~
>gis−dre−mir−437
guucgguuugguacgcuuuuauggccguuuccacugucaaaaguguaccgaaccaaaccg
>3p−matureseq
caaaaguguaccgaaccaaac
>3p−matureseq
caaaaguguaccgaaccaaacc
>3p−matureseq
caaaaguguaccgaaccaaaccg
>5p−matureseq
gguuugguacgcuuuuauggcc
>5p−matureseq
guucgguuugguacgcuuuuauggc
>3p−matureseq
ucaaaaguguaccgaaccaaac
>5p−matureseq
ucgguuugguacgcuuuuauggc
>5p−matureseq
uucgguuugguacgcuuuuauggc
~~~~~~
>gis−dre−mir−438
guugaaaucaaagguggcuaguugggcagcccaucucuguucuaaggcaccuaguuuagc
>5p−matureseq
guugaaaucaaagguggcuaguugggca
>3p−matureseq
ucuguucuaaggcaccuaguuuagc
>5p−matureseq
ugaaaucaaagguggcuaguugggcagccc
~~~~~~
>gis−dre−mir−439

guugggggcaaucaguggagcucaugcugcugcugcuccacugcucugccuccccu
>3p-matureseq
cuccacugcucugccuccccu
>5p-matureseq
guugggggcaaucaguggagcu
>5p-matureseq
ugggggcaaucaguggagcu
~~~~~~
>gis-dre-mir-440
uaaacaaguuuagcacagacuuugaugucagcaaagucugugcuaaacuuguuuaga
>5p-matureseq
uaaacaaguuuagcacagacu
>3p-matureseq
ucugugcuaaacuuguuuaga
~~~~~~
>gis-dre-mir-441
uaaacaaguuuagcacagacuuugcugacaucaaagucugugcuaaacuuguuuaga
>5p-matureseq
uaaacaaguuuagcacagacu
>3p-matureseq
ucugugcuaaacuuguuuaga
~~~~~~
>gis-dre-mir-442
uaacguuucgagcccacugacugucuuauaaagagccguggacuugaaacguug
>3p-matureseq
gagccguggacuugaaacguug
>5p-matureseq
uaacguuucgagcccacugacu
>5p-matureseq
uaacguuucgagcccacugacug
~~~~~~
>gis-dre-mir-443
uaccgacucacggucucucuccucgcucacaagcgccauuuuggagacagagagcugcgagucggcu
>3p-matureseq
agacagagagcugcgagucggcu
>5p-matureseq
uaccgacucacggucucucucc
~~~~~~
>gis-dre-mir-444
uacuugcucuaaacucccucagguuuaaucaucacacugaggagcuuagaccaagucaa
>5p-matureseq
uacuugcucuaaacucccucagg
>3p-matureseq
ugaggagcuuagaccaaguca
>3p-matureseq
ugaggagcuuagaccaagucaa
~~~~~~
>gis-dre-mir-445
uagucacauucaggugaguucuuugauuaucuugaauucaugguacuccuuggauguugacugga
>3p-matureseq
augguacuccuuggauguugac
>5p-matureseq
cacauucaggugaguucuuuga
>3p-matureseq

```
uacuccuuggauguugacugga
>5p-matureseq
uagucacauucaggugaguucu
>5p-matureseq
ucacauucaggugaguucuuug
>5p-matureseq
ucacauucaggugaguucuuuga
~~~~~~~~~~~~~~~~~~~~~~~~~~~~~~~~~~~~~~~~~~~~~~~~~~~
>gis-dre-mir-446
ucauagcagugcgauuguggcuguaaauacauccacgucacacugcuacgag
>3p-matureseq
auccacgucacacugcuacgag
>5p-matureseq
ucauagcagugcgauuguggcu
>5p-matureseq
ucauagcagugcgauuguggcug
>5p-matureseq
ucauagcagugcgauuguggcugua
>3p-matureseq
uccacgucacacugcuacgag
~~~~~~~~~~~~~~~~~~~~~~~~~~~~~~~~~~~~~~~~~~~~~~~~~~~
>gis-dre-mir-447
ucaucccucugcucuauccccuucaaagggacccuccaaagggauuagggcauaaggaug
>3p-matureseq
agggauuagggcauaaggaug
>5p-matureseq
ucaucccucugcucuaucccccu
~~~~~~~~~~~~~~~~~~~~~~~~~~~~~~~~~~~~~~~~~~~~~~~~~~~
>gis-dre-mir-448
ucugaaagcauagugccauggaccuuucuggagaccuugaauuauguaaccggagu
>3p-matureseq
accuugaauuauguaaccggagu
>5p-matureseq
ucugaaagcauagugccauggacc
~~~~~~~~~~~~~~~~~~~~~~~~~~~~~~~~~~~~~~~~~~~~~~~~~~~
>gis-dre-mir-449
ucugcauccaguguacauucagagaacggagacaugguguacgcuggagaacagacu
>3p-matureseq
guguacgcuggagaacagacu
>5p-matureseq
ucugcauccaguguacauuca
>5p-matureseq
ucugcauccaguguacauucag
~~~~~~~~~~~~~~~~~~~~~~~~~~~~~~~~~~~~~~~~~~~~~~~~~~~
>gis-dre-mir-450
ucuuggagaauaaauguuggaacggcuugccauauagcgugcucacaauauuuuuucucugaca
>3p-matureseq
ucacaauauuuuuucucugaca
>5p-matureseq
ucuuggagaauaaauguuggaac
~~~~~~~~~~~~~~~~~~~~~~~~~~~~~~~~~~~~~~~~~~~~~~~~~~~
>gis-dre-mir-451
ugagccacugauguccauaguaggaacaaaaaaauacuauggaagucuguggcugcuu
>3p-matureseq
```

acuauggaagucuguggcugcuu
>3p-matureseq
cuauggaagucuguggcugcuu
>5p-matureseq
ugagccacugauguccauagu
~~~~~~
>gis-dre-mir-452
ugagucuucugguucuugagucguucguucaucacgugacagaaucggaggacucgag
>5p-matureseq
agucuucugguucuugagucguuc
>5p-matureseq
gagucuucugguucuugagucguu
>3p-matureseq
ugacagaaucggaggacucga
>3p-matureseq
ugacagaaucggaggacucgag
>5p-matureseq
ugagucuucugguucuugagucguu
~~~~~~
>gis-dre-mir-453
uguaccaugcugguagccaguaugaaauagggcuugcugguaaccagcguugugcccc
>3p-matureseq
cugguaaccagcguugugccc
>3p-matureseq
cugguaaccagcguugugcccc
>3p-matureseq
ugguaaccagcguugugcccc
>5p-matureseq
uguaccaugcugguagccagu
>5p-matureseq
uguaccaugcugguagccagua
~~~~~~
>gis-dre-mir-454
uguagaucaguggauuagcagagcugaugugugcuguuacacagaucuacagc
>3p-matureseq
ugcuguuacacagaucuacagc
>5p-matureseq
uguagaucaguggauuagcaga
~~~~~~
>gis-dre-mir-455
ugugugauguaugugcauacuuauuugcaugcaugucugcaccuguauacacacaca
>3p-matureseq
ucugcaccuguauacacacaca
>5p-matureseq
ugugugauguaugugcauacu
~~~~~~
>gis-dre-mir-456
uguugcuauugaccuccauagugugguuuuuuuuuucuacgauagaagucauuaguuacggg
>3p-matureseq
gauagaagucauuaguuacggg
>5p-matureseq
uguugcuauugaccuccauagu
~~~~~~
>gis-dre-mir-457

```
uguuugacacuugaagggauuugcuugauuuuugucagcgauucaacuggugucagaguu
>3p-matureseq
cgauucaacuggugucagaguu
>5p-matureseq
uguuugacacuugaagggauuug
>5p-matureseq
uguuugacacuugaagggauuugcu
~~~~~~~~~~~~~~~~~~~~~~~~~~~~~~~~~~~~~~~~~~~~~~~~~~~
>gis-dre-mir-458
uugaccaucuucagacgugacuauauaucaguuugaccaucuucagacgugacuauauaucgguuuga
ugaucuucagac
>3p-matureseq
aucgguuugaugaucuucagac
>5p-matureseq
uugaccaucuucagacgugacua
>3p-matureseq
uuugaccaucuucagacguga
~~~~~~~~~~~~~~~~~~~~~~~~~~~~~~~~~~~~~~~~~~~~~~~~~~~
>gis-dre-mir-459
uuuauccuaucuagacuuuacccucuccugcagcauggugaagucuagaugggauucagc
>3p-matureseq
ugaagucuagaugggauucagc
>5p-matureseq
uuuauccuaucuagacuuuac
>5p-matureseq
uuuauccuaucuagacuuuacc
~~~~~~~~~~~~~~~~~~~~~~~~~~~~~~~~~~~~~~~~~~~~~~~~~~~
```
