# Supplementary material for: Deep sequencing of small RNA facilitates tissue and sex associated microRNA discovery in zebrafish
Source: BMC Genomics. 2015 Nov 16;16:950. doi: 10.1186/s12864-015-2135-7 (PMC4647824; doi:10.1186/s12864-015-2135-7)
Supplement: Additional file 10: — Figure showing the schematic representation of obtaining specific novel pre-miRNAs for embryo. The coloured circles represent the set of predicted novel pre-miRNAs for each tissue sample. The 78 predicted novel pre-miRNAs of embryo were compared with the set of predicted novel pre-miRNAs of other tissues to find the ones that matched. The total set of matched novel pre-miRNAs were 59. Therefore the unmatched set of 19 was considered as specific novel pre-miRNAs for embryo. This procedure was followed for the other tissue samples to obtain the novel pre-miRNAs specific to them. (PDF 30 kb) [file 12864_2015_2135_MOESM10_ESM.pdf]

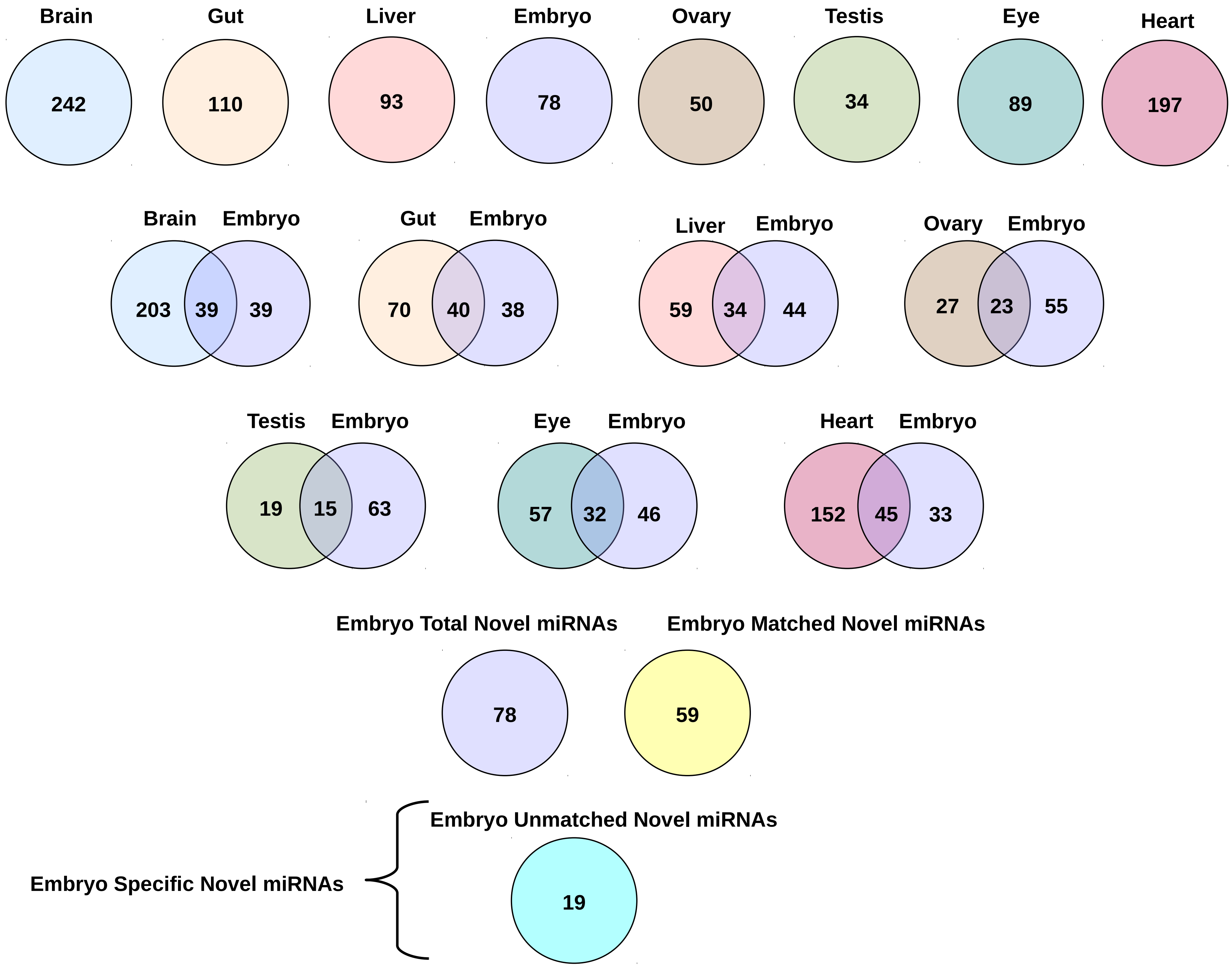

Brain
242
Gut
110
Liver
93
Embryo
78
Ovary
50
Testis
34
Eye
89
Heart
197
Brain
Embryo
203
39
39
Gut
Embryo
70
40
38
Liver
Embryo
59
34
44
Ovary
Embryo
27
23
55
Testis
Embryo
19
15
63
Eye
Embryo
57
32
46
Heart
Embryo
152
45
33
Embryo Total Novel miRNAs
78
Embryo Matched Novel miRNAs
59
Embryo Specific Novel miRNAs
Embryo Unmatched Novel miRNAs
19
