# Supplementary material for: Deep sequencing of small RNA facilitates tissue and sex associated microRNA discovery in zebrafish
Source: BMC Genomics. 2015 Nov 16;16:950. doi: 10.1186/s12864-015-2135-7 (PMC4647824; doi:10.1186/s12864-015-2135-7)
Supplement: Additional file 12: — Comprises of additional details of the methods, including the options and parameters of the tools used for processing of the reads, known miRNA expression profile generation, normalization and clustering and novel miRNA prediction pipeline. (PDF 1468 kb) [file 12864_2015_2135_MOESM12_ESM.pdf]

Processing of the reads

The reads were first subjected to adapter removal through the cutadapt program. The trimmed reads were then collapsed to remove redundancy and to obtain a unique sequence fasta file through the mapper module of miRDeep2 package.

The cutadapt command line used:

./cutadapt -b TGGAATTCTCGGGTGCCAAGG -g GTTCAGAGTTCTACAGTCCGACGATC <untrimmed reads fastq file> > <trimmed reads fastq file>

-b = adapter found anywhere (5' or 3' or middle); -g = adapter found at the 5' end

The mapper.pl command line used:

perl mapper.pl <trimmed reads fastq file> -e -h -i -j -l 18 -m -s <unique reads fasta file> -v

-e = input file is fastq format; -h = parse to fasta format; -i = convert rna to dna alphabet (to map against genome); -j = remove all entries that have a sequence that contains letters other than a,c,g,t,u,n,A,C,G,T,U,N -l int = discard reads shorter than int nts, default = 18; -m = collapse reads; -s file = print processed reads to this file; -v = outputs progress report

The headers of the unique fasta file comprise of a running number and the frequency of the particular trimmed read.

Known miRNA expression profile generation, normalization and clustering

The known mature miRNA expression profile was generated by using the quantifier module of the miRDeep2 package that gives the read counts for the known miRNAs.

The quantifier.pl command line used:

perl quantifier.pl -p <zebrafish precursor miRNA fasta file> -m <zebrafish mature miRNA fasta file> -r <unique reads fasta file> -t Zebrafish

-p precursor.fa = miRNA precursor sequences from miRBase; -m mature.fa = miRNA sequences from miRBase; -r reads.fa = user's read sequences; -t = species

The raw reads expression profile generated for all the replicates of the samples were subjected to Trimmed Mean of M-values (TMM) normalisation using the bioconductor package edgeR. The normalized expression profiles for all the replicates of all the samples were then subjected to

hierarchical clustering as well as PCA clustering for quality control purpose as well as to look at the similarity among the samples.

Novel miRNA prediction pipeline

The un-annotated sequences left after the elimination pipeline, were used for the novel miRNA prediction. For this purpose, the un-annotated sequence files of the replicates of a particular tissue were combined and first mapped to the Zebrafish genome using mapper.pl module and then subjected to the miRDeep2.pl module to obtain the novel miRNAs.

The mapper.pl command line used:

perl mapper.pl config.txt -c -d -i -j -l 18 -m -p <zebrafish genome bowtie index file> -q -s <Sample.fa> -t <Sample.arf >-v

config.txt = contains the names and three letter codes of the files that need to be combined

The Sample.fa file is a normal fasta file that contains non-redundant read sequences. The sequence headers that serve as unique identifiers are made up of a three letter code prefix, a running number in its middle part, and the frequency of that sequence at the end.

The Sample.arf file contains information on the alignments of the sample reads to the reference genome and is further used as input for the miRDeep2 module. The arf format is a standard file format of miRDeep2.

-c = input file is fasta format; -d = input file is a config file ; -i = convert rna to dna alphabet (to map against genome); -j = remove all entries that have a sequence that contains letters other than a,c,g,t,u,n,A,C,G,T,U,N -l int = discard reads shorter than int nts, default = 18; -m = collapse reads; -p genome = map to genome (must be indexed by bowtie-build); -q = map with one mismatch in the seed -s file = print processed reads to this file; -t file = print read mappings to this file; -v = outputs progress report

The miRDeep2.pl command line used:

perl miRDeep2.pl <Sample.fa> <zebrafish genome.fa> <Sample.arf> <zebrafish mature miRNA fasta file> <related species mature miRNA fasta file> <zebrafish precursor miRNA fasta file> -t Zebrafish -P 2>report.log

-t species = species being analyzed - this is used to link to the appropriate UCSC browser entry

-P = use this switch if mature_ref_miRNAs contain miRBase v18 identifiers (5p and 3p) instead of previous ids from v17
